# Metallodrugs are unique: opportunities and challenges of discovery and development†

**DOI:** 10.1039/d0sc04082g

**Published:** 2020-11-12

**Authors:** Elizabeth J. Anthony, Elizabeth M. Bolitho, Hannah E. Bridgewater, Oliver W. L. Carter, Jane M. Donnelly, Cinzia Imberti, Edward C. Lant, Frederik Lermyte, Russell J. Needham, Marta Palau, Peter J. Sadler, Huayun Shi, Fang-Xin Wang, Wen-Ying Zhang, Zijin Zhang

**Affiliations:** Department of Chemistry, University of Warwick Gibbet Hill Road Coventry CV4 7AL UK Cinzia.Imberti@Warwick.ac.uk P.J.Sadler@Warwick.ac.uk; Department of Chemistry, Technical University of Darmstadt Alarich-Weiss-Strasse 4 64287 Darmstadt Germany

## Abstract

Metals play vital roles in nutrients and medicines and provide chemical functionalities that are not accessible to purely organic compounds. At least 10 metals are essential for human life and about 46 other non-essential metals (including radionuclides) are also used in drug therapies and diagnostic agents. These include platinum drugs (in 50% of cancer chemotherapies), lithium (bipolar disorders), silver (antimicrobials), and bismuth (broad-spectrum antibiotics). While the quest for novel and better drugs is now as urgent as ever, drug discovery and development pipelines established for organic drugs and based on target identification and high-throughput screening of compound libraries are less effective when applied to metallodrugs. Metallodrugs are often prodrugs which undergo activation by ligand substitution or redox reactions, and are multi-targeting, all of which need to be considered when establishing structure–activity relationships. We focus on early-stage *in vitro* drug discovery, highlighting the challenges of evaluating anticancer, antimicrobial and antiviral metallo-pharmacophores in cultured cells, and identifying their targets. We highlight advances in the application of metal-specific techniques that can assist the preclinical development, including synchrotron X-ray spectro(micro)scopy, luminescence, and mass spectrometry-based methods, combined with proteomic and genomic (metallomic) approaches. A deeper understanding of the behavior of metals and metallodrugs in biological systems is not only key to the design of novel agents with unique mechanisms of action, but also to new understanding of clinically-established drugs.

## Abstract 2

The vital roles of metals in nutrients and medicines are not accessible to purely organic compounds.

## Graphical abstract

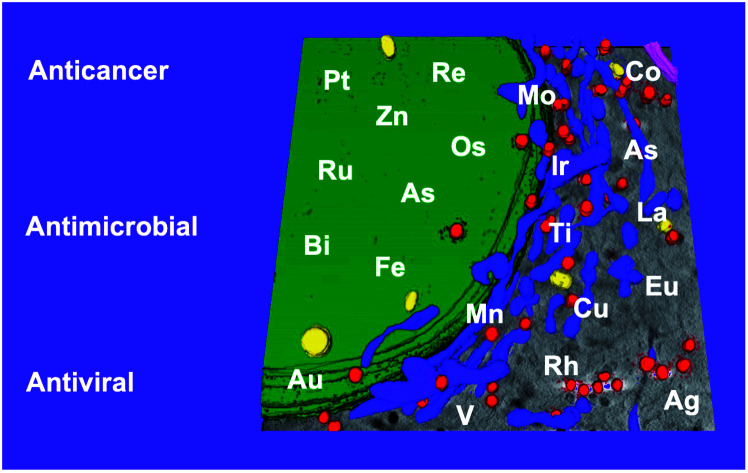

## 1. Introduction

Metallodrugs have been used for centuries, but only now are methods and techniques becoming available to characterise the drugs precisely, identify their target sites, and elucidate their unique mechanisms of action.

**Fig. 1:**
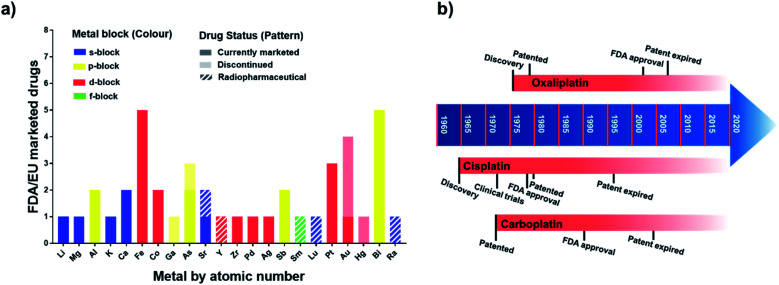
(a) Metallodrugs approved in US and/or EU countries classified by metal centre. Different formulations of the same active ingredient are not included, nor are pharmaceuticals where the metal represents only the counter ion. Imaging/diagnostic agents, food supplements, agents used as anaesthetics and in implants are also excluded. A comprehensive list of the agents included is in Table S1.† Remarkably, the total number of clinically-approved metallodrugs is about half of that for kinase inhibitors, a single class of organic drugs (see https://www.ppu.mrc.ac.uk/list-clinically-approved-kinase-inhibitors). (b) Timeline describing the development of anticancer drugs cisplatin (**Pt1**), carboplatin (**Pt2**) and oxaliplatin (**Pt3**).

The mixture of 3-amino-4-hydroxyphenyl-As^III^ compounds known as Salvarsan (**As1**), containing acyclic As_3_ and As_5_ species,^1^ was introduced by Ehrlich in the early 20^th^ century. Salvarsan was marketed by Hoechst as a treatment for syphilis and heralded the beginning of modern chemotherapy. More than half a century later, FDA approval in 1978 of *cis*-[Pt^II^Cl_2_(NH_3_)_2_] (cisplatin, **Pt1**) for the treatment of testicular cancer generated a huge surge of clinical interest in metallodrugs, and the birth of medicinal inorganic chemistry. Since then, a plethora of metallodrugs have been investigated as anticancer agents, antimicrobials, antivirals, and for many other indications.^2^ However, very few complexes have been successfully translated into the clinic. A histogram showing the number of metal and metalloid active ingredients in therapeutic drugs approved in the US and/or in Europe is reported in Fig. 1 together with a timeline of platinum drug development. The list of active ingredients is in Table S1.†

Metal-based agents are also less investigated in preclinical screenings and, consequently, in clinical trials, suggesting that their potential remains largely untapped. An international antibiotic screening centre recently noted that although metal-containing agents represent a minority of compounds submitted, they display a 10× higher hit-rate towards ESKAPE pathogens than purely organic molecules.^3^ Metal compounds often do not follow the guidelines for drug-like properties of organic molecules. For example, Lipinski's rule of 5 predicts oral bioavailability of new agents,^4^ but includes a requirement of a MW < 500 Da, problematic for many third-row transition metal (TM) compounds,^5^*e.g.* the gold drug auranofin (**Au1**) (MW 678 Da). Molecular volume (MV) rather than MW has been proposed as an alternative for metallodrugs and utilised to build a library of metallofragments for drug discovery.^6^

Here we highlight some of the major challenges in translating metallodrugs into preclinical development. We will begin with the chemistry of metallodrugs, describing modes of activation, which often generate active pharmacophores, then move to *in vitro* screening, describing examples of complexes investigated for different indications and discussing the types of assay used to assess their potential for clinical translation. The identification of intracellular targets for metallodrugs is important for elucidating molecular mechanisms of action, but is a major challenge. While early research in the field has focused almost exclusively on DNA as a target for metallodrugs, we show here how, for example, new omics techniques allow identification of different target sites and hence mechanisms of action.

**Fig. 2:**
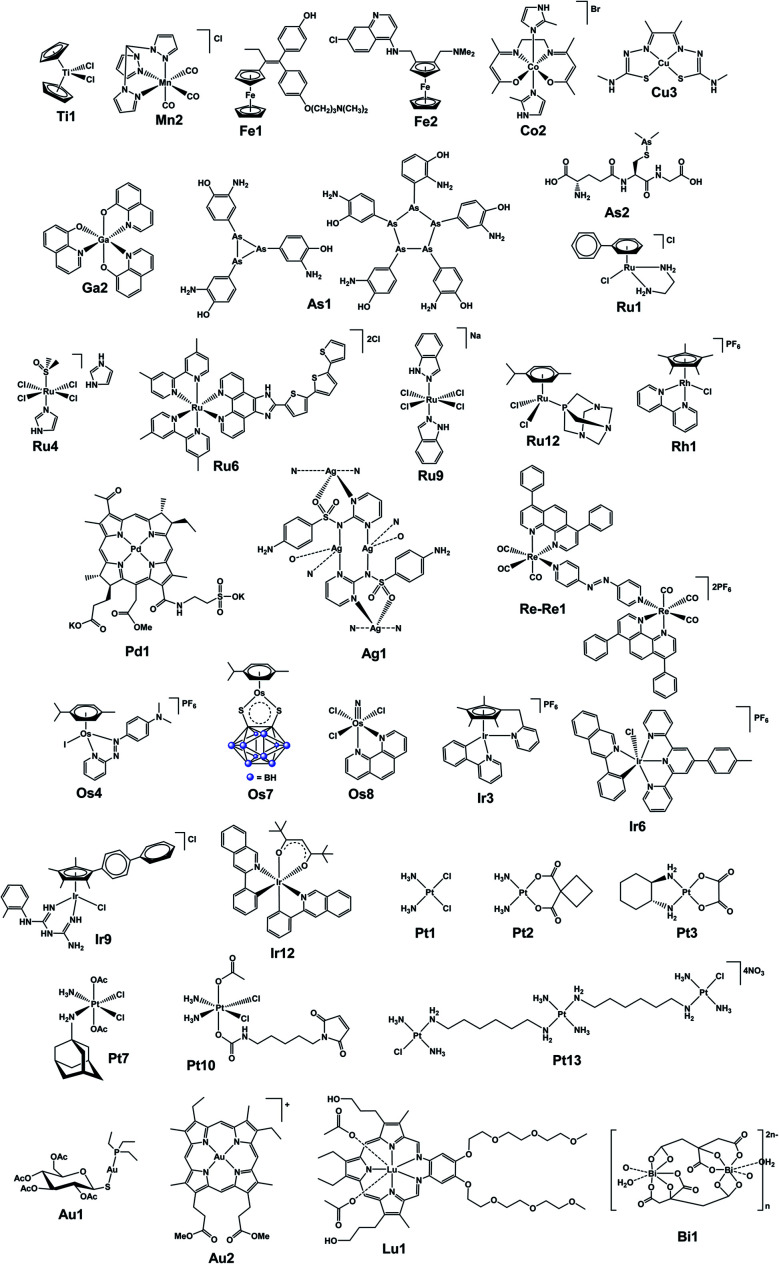
Structures of selected (candidate) metallodrugs. Others labelled in the text are in the ESI, Section 6.†

In the final section, we focus on analytical techniques, which can reveal metal speciation in solution and biological systems and track the distribution of metallodrugs in cells and *in vivo*, exploiting the cutting-edge metal-specific techniques now available. The chemical structures of selected metallodrugs are in Fig. 2, others (those marked with †) are given in Section 6 of the ESI.†

While this perspective focusses on metallodrugs and their interactions with biological molecules to produce a therapeutic effect, the targeting of metal-containing proteins, such as metalloenzymes, represents another important and complementary area of research. The role of metalloenzymes as medicinal targets and the use of metal-binding pharmacophores (MBPs) to inhibit them has recently been reviewed and will not be discussed here.^7^ Metallodrugs can also target metalloenzymes, either *via* incorporation of MBPs in their structure (*e.g.* as an axial ligand in Pt^IV^ agents)^8^ or directly through their metal centre (*e.g.* by replacing a metal ion in the active site).^9^ A few examples of metalloenzyme-targeted metallodrugs will be described.

## 2. Activation mechanisms

Metal-based therapeutics offer versatile electronic and structural features, including a range of oxidation states, coordination geometries, type and number of ligands. They offer novel chemistry, including different types of ligand substitution, metal- and ligand-based redox processes, and catalytic cycles. Unlike organic drugs, they are often ‘prodrugs’ which undergo activation *en route* to or at the target site. This typically involves the dissociation or displacement of one or more labile ligands, chelate ring-opening, or a change in oxidation state (or energetic state) of the metal and/or ligand(s). Alternatively, an external stimulus (*e.g.* light, radiation, sound, heat) can selectively activate metallodrugs at the target site.

### 2.1 Activation *via* hydrolysis

Hydrolysis is a common activation mechanism for TM drugs, involving the displacement of weakly bound σ-donor ligands by H_2_O. Marked differences in lability of ligands for different metal ions is illustrated by their aqua–ligand exchange rates, with time scales spanning 20 orders of magnitude (nanoseconds to years) from alkali metal ions to low-spin heavy TM ions.^10^ Such inertness of heavier TMs can be exploited in drug design. Importantly, an appropriate choice of ligands can modulate the ‘inertness’ of metal ions.

#### 2.1.1 Square-planar Pt^II^ complexes

**Fig. 3:**
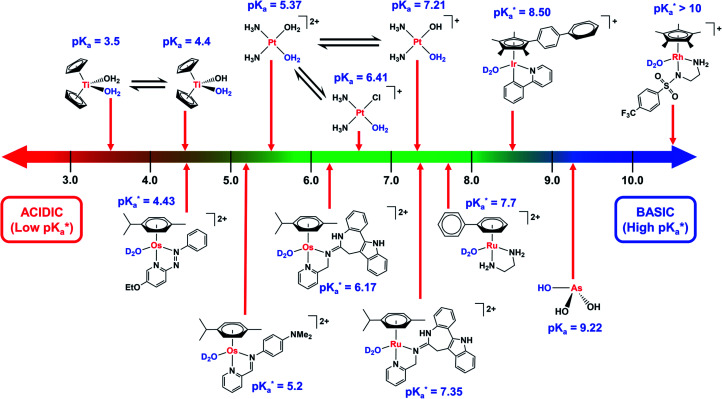
Comparison of the p*K*_a_ values of aquated species of metal-based complexes. 
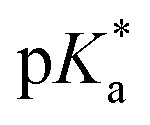
 values refer to M–OD_2_ complexes based on pH* (pH meter reading not corrected for the effect of deuterium on the electrode).

Cisplatin (**Pt1**, *cis*-[Pt^II^Cl_2_(NH_3_)_2_]) undergoes activation *via* hydrolysis (Fig. 3).^11^ Extracellularly, where [Cl^−^] > 100 mM, hydrolysis is suppressed. Inside cells, where [Cl^−^] is lower (*ca.* 23 mM in cytoplasm and ∼4 mM in the nucleus),^12^ aquation occurs more readily, resulting in more reactive mono-aquated species, [Pt^II^(OH_2_)Cl(NH_3_)_2_]^+^ and di-aquated [Pt^II^(NH_3_)_2_(OH_2_)_2_]^2+^, which readily bind to DNA bases G and A, and also less reactive hydroxido species are formed (p*K*_a_ values of [Pt^II^(OH_2_)Cl(NH_3_)_2_]^+^ and [Pt^II^(NH_3_)_2_(OH_2_)_2_]^2+^*ca.* 6.4, and 5.4/7.2, respectively).^13^ Only *ca.* 1% of intracellular cisplatin reacts with DNA,^14^ where it causes cell cycle arrest and apoptosis.^15^ ‘Soft’ Pt^II^ binds strongly to ‘soft’ cysteine thiolate groups in proteins.^16^ The tripeptide l-glutathione (γ-l-Glu-l-Cys-Gly, GSH, 2–10 mM in cells) can detoxify Pt^II^, especially in resistant cancers.^17^ Such reactions can also result in off-target side-effects.^11^ Since hydrolysis rates depend on the ligands, a chelated dicarboxylate, relatively inert towards hydrolysis, was introduced in the second-generation drug carboplatin (**Pt2**), which displays reduced nephrotoxicity.^18^

#### 2.1.2 Ti^IV^ dichloride complexes

[Ti^IV^Cp_2_Cl_2_] (titanocene dichloride, **Ti1**) was in clinical anticancer trials for about 10 years,^19^ but its progression into phase II clinical trials was hampered by its strong tendency to hydrolyse,^20^ and pH-dependent interactions with DNA.^21^ Not only are the Cl^−^ ligands labile but also Cp is displaced in reactions with serum transferrin, where Ti^IV^ binds to the Fe^III^ sites, providing a delivery system for Ti^IV^ into cancer cells.^22^ To reduce hydrolysis rates, later titanocene derivatives included the addition of bulky aryl substituents, tethering Cp rings together, and even tethering Cp rings to labile groups to reduce ligand displacement (**Ti2**†).^23^

#### 2.1.3 Ru^II^ and Os^II^ half-sandwich complexes

Similarly to cisplatin, half-sandwich pseudo-octahedral Ru^II^ and Os^II^ η^6^-arene diamine anticancer complexes, [Ru^II^/Os^II^(η^6^-arene)(N,N)Cl]^+^, such as RM175 (**Ru1**) also hydrolyse and bind to DNA, but monofunctionally as they have only one labile monodentate ligand.^24^ The choice of ligands plays a vital role in tuning the hydrolysis rate and reactivity. For Ru^II^, hydrolysis rate decreases with the monodentate ligand in the order Cl ≈ Br > I > N_3_.^25^ Hydrolysis rate tends to increase with the electron donating ability of the arene, and the bidentate ligand has a strong effect on hydrolysis, increasing drastically when changing from neutral N- to anionic O-donors.^12^ Whereas aqua ligands are labile, hydroxido ligands are less so and prone to formation of bridged species. This can happen in culture media, giving inert hydroxo-bridged species in the case of [Os^II^(η^6^-*p*-cym)(acac)Cl] (**Os1**†), which are inactive towards cancer cells.^26^ Os^II^ analogues of Ru^II^ arene chlorido complexes can hydrolyse up to 100× more slowly (*e.g.***Os2**†*vs.***Ru1**),^26^ and the aqua adducts of Os^II^ arene paullone-based bidentate ligand complexes (*e.g.***Os3**†) are 1.2 p*K*_a_ units more acidic than their Ru^II^ counterparts (**Ru2**†).^27^

**Fig. 4:**
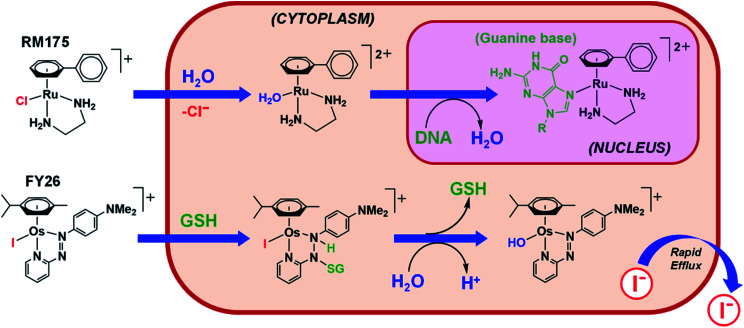
Hydrolytic activation of two types of half-sandwich complex; RM175 (**Ru1**), bearing a σ-donor bidentate ligand (en), for which hydrolysis is activated by reduced [Cl^−^] levels in cells; FY26 (**Os4**), bearing a strong π-acceptor ligand (azpy), for which hydrolysis is activated by GSH attack on the azo-bond.^24,29^

Strong σ-donor ligands (*e.g.* en, acac and pico) tend to promote fast hydrolysis and produce basic aqua adducts (p*K*_a_ > 7). π-Acceptor ligands promote slower hydrolysis and more acidic aqua complexes (Fig. 3). Os^II^ azopyridine (azpy) chlorido complexes can even be stable towards hydrolysis when heated under reflux with AgNO_3_.^28^ Complex FY26, [Os^II^(η^6^-*p*-cym)(azpy-NMe_2_)I]PF_6_ (**Os4**), is extremely stable and hydrolysis of the Os–I bond is negligible in aqueous media over 24 h.^28^^131^I-radiolabelling studies revealed that rapid cleavage of the Os–I bond is promoted in cancer cells.^29^ The mechanism appears to involve attack on the azo-bond by GSH (Fig. 4).^29^ This is accompanied by the generation of ROS.^30^ Iminopyridine (impy) ligands are similar in character to azpy, exhibiting weaker π-acceptor ability, and aqua adducts possess both DNA-binding and ROS-generating capabilities.^31^ Ang *et al.* screened 442 Ru^II^ arene impy complexes for their anticancer activity against A2780 ovarian cancer cells.^32^ All but one of the six lead complexes were stable towards hydrolysis and incorporate bulky substituents on their arene ligands and fused aromatic groups on their impy ligands (*e.g.***Ru3**†).

#### 2.1.4 Rh^III^ and Ir^III^ Cp complexes

The residence times for water molecules in the first coordination shell of aqua Rh^III^ and Ir^III^ are extremely long (10^8^ to 10^10^ s).^33^ The Ir^III^ analogue of NAMI-A (**Ir1**†) is inert towards hydrolysis and exhibits poor antiproliferative activity.^34^ However, introduction of Cp* onto [Ir^III^(H_2_O)_6_]^3+^ dramatically increases hydrolysis rates by 14 orders of magnitude.^35^ As a consequence, a range of Rh^III^ and Ir^III^ Cp^X^ chlorido anticancer complexes with various *N*,*N*-, *N*,*O*-, *O*,*O*- and *C*,*N*-coordinated ligands all hydrolyse rapidly (minutes).^36^ Similarly to Ru^II^ and Os^II^ arene complexes, Rh^III^ exhibits higher p*K*_a_ values for its aqua species than its heavier congener Ir^III^. For example, for [Rh^III^/Ir^III^(η^5^-Cp*)(en)H_2_O]^2+^ the Rh^III^ complex is 1.7 p*K*_a_ units less acidic.^37,38^ Interestingly, deprotonation of Cp* in complexes such as [Rh^III^(η^5^-Cp*)(bipy)(OH)]^+^ (aqua species of **Rh1**) can occur under physiological conditions to give Rh^I^-fulvene species, which can undergo [4+2] cycloaddition Diels–Alder reactions with biologically relevant dienes (*e.g.* isoprene and (9*Z*,11*E*)-linoleic acid).^39^ Many chlorido complexes of Ir^III^ Cp* with bipy, phpy, en, pico and acac bidentate ligands, hydrolyse rapidly and are anticancer inactive. However, introduction of extended arenes to Cp* (Cp^Xphen^ and Cp^Xbip^) can reduce the hydrolysis rate, aqua-complex p*K*_a_, and switch on activity.^40^ Similarly, azpy ligands can also render Ir^III^ Cp* complexes inert to hydrolysis, switching on their activity and promoting catalysis of GSH oxidation.^41^ Switching the monodentate ligand from Cl^−^ to a neutral pyridine (py) significantly slows hydrolysis and the py ligand in [Ir^III^(Cp^xbiph^)(phpy)(py)]^+^ (**Ir2**†) increases anticancer activity 3-fold.^42^ Moreover, Ir^III^ complexes with a Cp* ligand tethered to a ‘labile’ pyridine ligand (*e.g.***Ir3**) are extremely potent and interestingly, appear to be activated *via* chelate ring opening in DMSO^43^ – a commonly used solvent in biological assays.

### 2.2 Redox activation

Oxygen is essential for energy production by eukaryotic organisms, but can also be lethal through generation of ROS.^44^ The disturbance of the redox balance is an effective anticancer strategy, owing to the distinct redox vulnerability of cancer cells, including hypoxia.^45–47^ Metal complexes can alter the cellular redox balance directly through reduction or oxidation at metal or ligand centres, or indirectly through interaction with biomolecules in redox pathways.^47^ Ideally, redox activation of metal complexes results in the formation of excess cytotoxic species selectively in cancer cells, thereby reducing side effects.^45^

#### 2.2.1 Metal reduction

Hypoxia can facilitate reduction and activation of inert metal complexes, *e.g.* Pt^IV^, Ru^III^, Co^III^, selectively in tumours.^45^ In normal, oxygenated cells, these prodrugs are reduced but rapidly re-oxidised and deactivated, whilst in hypoxic cancer cells, re-oxidation occurs slowly.^48^ Octahedral low-spin 5d^6^ Pt^IV^ complexes are more stable than classical Pt^II^ agents in biological media, and can be reduced by bio-reductants such as GSH, ascorbate, NAD(P)H, and cysteine-containing proteins, releasing active ligands and DNA-binding Pt^II^ species.^45,49^ Four Pt^IV^ complexes have entered clinical trials (*i.e.* tetraplatin (**Pt4**†), iproplatin (**Pt5**†), satraplatin (**Pt6**†) and LA-12 (**Pt7**†)), although none have been approved due to side effects and low efficacy.^45,50^ A Ru^II^–Pt^IV^ conjugate (**Ru-Pt1**†) bridged by 1,6-diaminohexane undergoes Pt^IV^ reduction to release cytotoxic Pt^II^, histone deacetylase inhibitor phenylbutyrate, and photosensitive Ru^II^ (480 or 595 nm irradiation), thus exerting a multi-targeting and multi-action effect.^51^ Ru^III^ complexes NAMI-A (**Ru4**) and KP1019 (**Ru5**†) passed phase I clinical trials (NAMI-A was terminated in phase II due to low activity).^52,53^ The Ru^II^ complexes formed upon reduction retain an octahedral structure, and can bind to DNA and proteins.^52^ In contrast, for Co^III^ prodrugs cytotoxicity is often attributable to the ligands (*e.g.* nitrogen mustards) released upon reduction, rather than the metal itself.^53^

#### 2.2.2 Ligand reduction

Piano-stool Ru^II^ arene complexes [Ru(η^6^-bip)(azpy-NMe_2_)X]^+^ (X = I, OH) are activated by reductive addition of GSH to give GSSG, and elevate the ROS levels in cancer cells.^54^ The *meso*-carbon in the porphyrin ring in AuMesoIX (**Au2**) can undergo nucleophilic aromatic substitution with thiols, and modify cysteine thiols of cancer-associated proteins.^55^

#### 2.2.3 Metal oxidation

Ferrocene (Fc) derivatives can undergo Fenton-type reactions in cells, exploit the overproduction of H_2_O_2_ in cancer cells and generate HO˙ radicals which cleave DNA.^56^ Ferrocifen (**Fe1**), the ferrocenyl analogue of hydroxytamoxifen, is highly active towards breast cancer and independent of hormone expression.^57^ With the Fc^+^/Fc redox couple, **Fe1** readily loses 2e^−^ and 2H^+^ to form a quinone methide intermediate that is stable and cytotoxic. ROS are generated in cells treated with ferrocifens, but not hydroxytamoxifen derivatives. The anti-malarial agent Ferroquine (**Fe2**) also exerts cytotoxicity by formation of Fe^III^ species and HO˙ radicals.^58^ Ferrocene N-heterocycle-linked Ru^II^*p*-cym complexes (*e.g.***Ru-Fe1**†) show a correlation between the cytotoxicity and the Fc^+/0^ reduction potentials, consistent with facile oxidation to give ferrocenium, and subsequent ROS generation.^59^

#### 2.2.4 Ligand oxidation

The hydrolysis product [Ru^II^(η^6^-bip)(en)H_2_O]^2+^ of the anticancer complex [Ru^II^(η^6^-bip)(en)Cl]^+^ (**Ru1**) reacts initially with GSH to form [Ru^II^(η^6^-bip)(en)(SG)]^+^.^24^ However, oxidation to the more labile sulfenato species, [Ru^II^(η^6^-bip)(en)(OSG)]^+^, can occur even under hypoxic conditions, leading to facile displacement of the sulfenate by N7-cGMP. Such Ru-induced thiol oxidation (to sulfonate) can also occur in glutathione *S*-transferase, an enzyme overexpressed in solid tumours.^60^ The iodide ligand in photoactive *trans*-[Pt^IV^(en)(OH)_2_I_2_] can be attacked by cellular bioreductants (*e.g.* GSH to generate GSSG) and liberate I^−^, with reduction of Pt^IV^ to Pt^II^.^61^

### 2.3 Photoactivation

Metallodrugs can be selectively activated with high spatial resolution in cancer cells in (i) photodynamic therapy (PDT), (ii) photothermal therapy (PTT), and (iii) photoactivated chemotherapy (PACT).

PDT is clinically-approved, minimally invasive, and employs a photosensitiser (PS) and spatially-controlled light (typically red 600–800 nm, the “therapeutic window”) to kill cancer cells in a catalytic manner in the presence of oxygen.^62^ Longer wavelength light penetrates more deeply and is less toxic to cells. Metal complexes provide an alternative to organic PDT treatments, currently dominated by porphyrins and their analogues.^63^ The absorption of a photon promotes an electron from the singlet ground state to a singlet excited state which can decay non-radiatively *via* intersystem crossing to a low-lying triplet state. From this state, an electron can be transferred to biological substrates to form radicals that react with oxygen to generate ROS, causing oxidative stress, leading to cell death (type I). There can also be direct energy transfer from the PS excited triplet state to ground state triplet oxygen (^3^O_2_) to generate highly reactive singlet oxygen (^1^O_2_), with a diffusion distance <300 nm and lifetime <3 μs in cell nuclei (type II).^64^ Type II processes are dominant in tissues and clinical trials of a ruthenium-based PS TLD-1433 (**Ru6**), which mainly relies on type II processes, are ongoing. However, the only approved TM-based PS, TOOKAD®-soluble (palladium bacteriopheophorbide monolysine taurine, **Pd1**), exclusively undergoes type I photoreactions to generate hydroxyl and superoxide radicals.^65^ Selected metal-based photosensitisers are in Table 1. For compounds in clinical trials, their National Clinical Trial (NCT) identifier and details of the trials are freely accessible from the database https://www.clinicaltrials.gov/.

**Table 1:** Selected photoactivatable metal anticancer drugs and drug candidates classified according to their development phase

M	Agent	Activation	Development stage	Indication	Ref.
**Clinically approved**					
Al^III^	Photosens® (**Al1**†)	PDT	Marketed in Russia	Stomach, lung, oesophagus and other cancers	66
Pd^II^	TOOKAD®-soluble (**Pd1**)	PDT	Marketed in Israel, Mexico, EU and EEA	Localised prostate cancer	67

**Clinical trials** a					
Ru^II^	TLD-1433 (**Ru6**)	PDT	Phase II	Non-Muscle Invasive Bladder Cancer (NMIBC) Refractory to BCG	67
			NCT03945162		
Lu^III^	Lutrin® (**Lu1**)	PDT	Phase I	Locally recurrent prostate cancer	66
			NCT00005067^b^		
Au^0^	AuroLase™	PTT	Multi-centre study	Prostate cancer	68
	Au nanoshell, SiO_2_ core, PEG coat		NCT04240639		

**Pre-clinical**					
Re^I^	Re-PLPG-NLS (**Re1**†)	PACT	—	Cervical (HeLa), prostate (PC-3) cancer cell lines	69
Os^II^	TLD-1829 (**Os5**†)	PDT	—	Bladder (HT1376), brain (U87) cancer cell lines	70
Ir^III^	Ir-HSA (**Ir4**†)	PDT	—	Lung (A549), liver (HepG2) cancer cell lines	71
Pt^II^	Pt-dithienylcyclopentenes (**Pt-Pt1**†)	PACT	—	Lung (A549), melanoma (A375), breast (MCF7), colon (SW620), ovarian (SKOV3) cancer cell lines	72

a NCT retrieved from https://www.clinicaltrials.gov/

b Not currently in clinical trials.

In PTT, metallodrugs convert photons to heat, leading to cell death. PTT agents are typically nanomaterials, including graphene oxide sheets and carbon nanotubes, as well as Cu^II^ nanocrystals, Bi^III^ nanorods, and Au^0^ nanoparticles (AuNPs).^73,74^ AuroLase® is currently in clinical trials for neoplasms of the prostate (NCT04240639). AuNPs are irradiated with near-infrared radiation (750–1400 nm), causing localised surface plasmon resonance which loses energy *via* radiative and non-radiative processes, resulting in hyperthermic cell death.^74^ Tumour damage occurs at >41 °C, however temperatures exceeding 50 °C are required for effective ablation.^73^

In PACT, light activation of an inert prodrug leads to the formation of photoproducts, which contribute to the therapeutic effects.^75,76^ For example, Pt^IV^ azido complexes [Pt^IV^L_1_L_2_(N_3_)_2_(OH)_2_] (L_1_, L_2_ = am(m)ine ligands) are reduced to cytotoxic Pt^II^ species upon visible light irradiation with concomitant release of azidyl radicals.^77^ A dithienylcyclopentene-Pt^II^ complex **Pt-Pt1**† exhibits increased cytotoxicity towards melanoma and colorectal cancer cells after photoswitching from its open to closed form.^72^ The oxygen-independent mechanism of PACT agents offers an advantage over PDT in hypoxic tumour environments. However, PACT agents are not catalytic, unlike PDT photosensitisers. This has led to the development of prodrugs which combine PDT with PACT, such as chlorin e6 conjugated Pt^IV^ micelles loaded on upconversion nanoparticles capable of producing oxygen upon photodecomposition, which is then self-utilised for PDT.^78^ Further examples of PACT agents are listed in Table 1. As yet, no metal-based PACT agents have entered clinical trials.

In principle, metal complexes which require activation by short wavelengths (*e.g.* blue light) can be activated using two photons of longer wavelength that penetrate more deeply (*e.g.* red/near-infrared light). This treatment can be highly specific (μm accuracy), however, the problem of rapid application to volumes as large as tumours has yet to be solved.^79^

### 2.4 Ionising radiation, sonodynamic and thermal activation

Ionising radiation (*e.g.* X-rays, γ-rays) can also activate or enhance the potency of metallodrugs. A radiosensitiser is a compound whose combination with ionising radiation generates a biological response greater than the sum of their single effects. This synergy was noted early in the clinical use of platinum drugs, which are now frequently combined with external beam radiotherapy.^80^ The mechanisms of radiosensitisation include enhancement of damage generated by X-ray radiation or inhibition of repair and resistance mechanisms.^80^ Several TM-based radiosensitisers (mainly groups 6–8) have been investigated preclinically (Table 2). Most recently, a cyclometallated Ir^III^ compound (**Ir5**†) was reported to localise in mitochondria and to produce increased radiosensitisation in cancer cells compared to cisplatin, attributable to increased ROS production upon X-ray irradiation.^81^

**Table 2:** Selected examples of metal-based agents activated by external stimuli

M	Type	Activation	Ref.
Ru^II^	Arene Ru^II^ complex (**Ru7**†)	Radiosensitiser	92
Ir^III^	Cyclometallated Ir^III^ complex (**Ir5**†)	Radiosensitiser	81
Co^II^	Co^II^ porphyrin (**Co1**†)	XPDT	93
Cu	Copper cysteamine nanoparticles	Scintillation XPDT	84
Ti^IV^	Hydrophilised TiO_2_ nanoparticles	Sonodynamic	94
Ga^III^	Gallium porphyrin-antiCEA antibody (**Ga1**†)	Sonodynamic	95
Ru^II^	Arene Ru^II^ complex with perfluorinated ligand (**Ru8**†)	Hyperthermia	89

Synchrotron stereotactic radiotherapy (SSR) is a type of external-beam irradiation, which exploits high-fluence monochromatic X-ray beams to treat cancer. The presence of heavy elements can enhance the therapeutic effect if the X-ray beam is tuned to the energy of the K-electrons of the sensitiser. Promising results were obtained for brain tumours in rats with SSR and cisplatin, although a similar effect was achieved with traditional linear accelerators.^82^

X-ray PDT (XPDT), resulting in the excitation of a photosensitiser and generation of singlet oxygen, can be achieved by direct PS excitation with X-rays or indirectly, by X-ray excitation of a luminescent material (scintillator), which in turn can activate the PS.^83^ Metalloporphyrins have been investigated for the direct approach,^83^ while inorganic-based nanoparticles have been explored for the indirect approach.^84^ The main advantage of XPDT is the ability to overcome the limited penetration depth of visible light.^85^

Moving from electromagnetic to mechanical waves, *sonodynamic therapy* was initially developed as a proxy for PDT to increase tissue penetration depth. Sonoluminescence, obtained by concentrating large amounts of energy from ultrasound, can excite a PS.^86^ Porphyrins and metalloporphyrins have shown promising results *in vitro*, and metal-based nanoparticles and metal–organic frameworks have been explored (Table 2).^87,88^

Metallodrugs can be selectively activated by heat, *e.g.* by conjugation to *thermoactivatable* fragments. For example, the solubility and cytotoxicity of RAPTA-derivatives containing perfluorinated ligands (**Ru8**†) are enhanced by heating (Table 2).^89^ Metalloenediynes (*e.g.***Pt8**†) are thermally-activated sources of the enediyne function, whose spontaneous cyclisation produces extremely potent 1,4-benzenoid diradical species.^90,91^

### 2.5 Catalytic metallodrugs

TM catalysts might offer low-dose agents that can carry out biocatalytic reactions on endogenous or exogenous substrates.^96^ Potential catalytic reactions include transfer hydrogenation, C–C bond cross-coupling, bond cleavage by hydrolysis or oxidation, azide–alkyne cycloaddition, and allyl carbamate cleavage.^97^ Successful catalysts in living systems require high conversion rates under physiological conditions, good enantioselectivity, and tolerance towards aqueous environments and nucleophilic poisoning.

#### 2.5.1 Reduction *via* transfer hydrogenation

Reduction of biomolecules in cancer cells by external catalysts can disrupt metabolic cellular processes crucial for cell survival. Nicotinamide adenine dinucleotide (NAD^+^) and reduced NADH are key cofactors for many reactions in cells involving redox homeostasis.^98^ A lower ratio of NAD^+^/NADH in the cytosol is observed in cancer cells compared to normal cells.^99^ Transfer hydrogenation reactions of NAD^+^/NADH have been achieved in cells using “piano-stool” metal complexes including Ir^III^ Cp* and Rh^III^ Cp* complexes, as well as Ru^II^ arene complexes.^100–102^ Formate can act as a hydride source at non-toxic concentrations.^96^ The catalytic mechanism involves initial formation of M-formate adducts, transfer of hydride to the metal and release of CO_2_ followed by hydride transfer to NAD^+^.^101^ Noyori-type Ru^II^ arene complexes with sulfonamido ethylenediamines as ligands show a 50-fold increase in cytotoxicity towards human ovarian cancer cells in the presence of formate. This type of catalysis increases the reductant pool and induces non-apoptotic cell death.^103^

In hypoxic cancer cells, pyruvate is reduced to lactate by lactate dehydrogenase and NADH. Chiral catalytic 16-electron Os^II^ complex **Os6**† can convert pyruvate to unnatural d-lactate enantioselectively in the presence of formate, and selectively for cancer cells over healthy cells.^104^

#### 2.5.2 Oxidation of NADH and thiols

Metal complexes can also catalyse the oxidation of NADH to NAD^+^, causing oxidative stress and apoptosis in cells.^42,105^ For example, half-sandwich Rh^III^ and Ir^III^ Cp* complexes reduce ketones in the presence of NADH as a cofactor.^105^ Ir^III^ complexes can transfer hydride to molecular oxygen, generating H_2_O_2,_ an ROS.^42^ The Ir^III^ photocatalyst, [Ir(ttpy)(pq)Cl]PF_6_ (**Ir6**) can oxidise NADH to generate NAD˙ radicals upon irradiation. The Ir–Cl bond in **Ir6** is resistant to hydrolysis and highly stable under 463 nm irradiation.^106^

The balance between reduced GSH and oxidised glutathione (GSSG) (cellular GSH : GSSG ranges from 30 : 1 to 100 : 1) also plays a critical role in mediating cellular redox processes.^107^ Arene Ru^II^ azpy complexes can catalyse GSH oxidation to GSSG,^54^ the first one-electron step being reduction of the azo-bond by GSH. Micromolar concentration of Ru^II^ catalyst oxidises millimolar GSH to GSSG. Ir^III^ Cp^xbiph^ complexes with hydrosulfide ligands achieve oxidation of GSH to GSSG without forming Ir–SG adducts and without hydrolytic mediation.^108^ Iodido Ir^III^ Cp* azpy anticancer complexes are inert but the catalyse oxidation of GSH to GSSG *via* azo-bond attack, and generate superoxide when O_2_ is present as an electron acceptor.^41^

#### 2.5.3 Degradation and cleavage of biomacromolecules

Strong Lewis acidic metal complexes can be designed for hydrolytic and oxidative cleavage. Metal complexes with tetra-*N*-methylated cyclam (TMC) ligands can cleave 36–43 amino acid Aβ peptides involved in Alzheimer's disease, with catalytic efficacy: Co^II^ > Zn^II^ > Cu^II^ > Ni^II^.^109^ Amino-terminal copper/nickel (ATCUN) peptide binding motifs such as Cu^II^-GGHK-R (**Cu1**†, where R is a target recognition sequence) can catalyse the cleavage of viral RNA and G-quadruplex telomeric DNA,^110,111^ in which the redox-active Cu^II^/Cu^III^ generates ROS to cleave bonds.

## 3. *In vitro* screening

Nowadays, discovery of new organic drugs typically begins with the choice of a protein or enzyme target, followed by target validation, assay development, high throughput screening, hit identification, lead optimisation, and selection of a candidate for clinical development. Rational design of metal complexes as enzyme inhibitors is feasible, as elegantly demonstrated by the work of Meggers, designing kinase inhibitors based on inert octahedral Ru(ii) scaffold.^112,113^

However, metal complexes are usually prodrugs transformed into active species by ligand exchange or redox reactions, and are likely to be multi-targeting. Hence, *phenotypic screening* (the ability of a molecule to alter a cell's phenotype -an observable characteristic) is usually the method of choice for discovery of metallodrugs. Phenotypic screening offers the potential advantage of revealing truly novel mechanisms of action.^114^

We discuss some promising metallodrug candidates under investigation for the treatment of cancer, inflammation and infections. For each indication we describe the main methods used for *in vitro* screening and highlight specific challenges. Nearly all the assays described below involve the culture of cells in a growth medium. Knowledge of the chemical composition of biological media is crucial for understanding the speciation of test metal complexes in screening assays. Metallo-prodrugs may be converted into different species before they interact with the cells during the screening.

### 3.1 Metallodrug speciation in biological assays

#### 3.1.1 Solubilising agents

Use of dimethylsulfoxide (DMSO) for solubilising drugs for cell testing is universal, but poses particular problems for TM complexes. DMSO can act as a *S*- or *O*-donor ligand, resulting in ligand substitution reactions.^115^ Even 0.5% v/v DMSO in culture medium is equivalent to 70 mM DMSO. Reactions with DMSO are temperature and time-dependent and the concentration of DMSO may vary between wells in a multi-well culture plate as the stock solution is diluted. For example, DMSO can have a significant effect on the cytotoxicity of platinum anticancer complexes such as cisplatin (*vide infra*). *S*-Bound DMSO with its high *trans* effect readily induces chloride and ammonia loss.^116^

#### 3.1.2 Culture media

Growth media contain many components (Tables S2–S4†), which can react with metal complexes (ligand substitution, redox) and transform them into new species (Tables S2–S4†).

Chemically-defined media include RPMI-1640, DMEM, MEM, and many others. RPMI-1640 (Table S2†) typically contains 20 amino acids (at concentrations ranging from 25–318 μM), all strong metal chelators, either bidentate, or tridentate *e.g.* 150 μM l-Asp, 136 μM l-Glu, 97 μM l-His, and 101 μM l-Met, a thioether. Other soft donors include the only thiol GSH (3.25 μM); Cys is present as the disulfide cystine.

These defined media are usually supplemented with *e.g.* 10% v/v foetal calf/bovine serum (FCS/FBS) or similar products (*e.g.* beef extracts for microbial culture), together with antibiotics such as penicillin (a thioether, typically at *ca.* 170 μM), which further complicates metal speciation. The compositions of these animal-derived products will differ between different batches, suppliers, and regions of the world. Typically, FBS contains *ca.* 0.3 mM bovine serum albumin (BSA) and 1 mM gamma globulin (Table S3†). Albumin has a range of drug-binding sites and may deactivate drugs, but it also may deliver metallodrugs to cells, even crossing cell membranes.^71,115^ The thiol content of albumin decreases with age (oxidation of Cys34).^117^ Albumin contains Cu^II^ and Zn^II^ binding sites. The FBS concentration of transferrin, which binds Fe^III^ and a variety of other metal ions strongly, is not always specified.^115,118^

Detailed metal speciation in these media is very complicated and depends not only on the type of medium used, but also on pH, aeration (CO_2_/O_2_), temperature and time. Reactions at low concentrations (*e.g.* micromolar), may be very slow. However, cell screens typically last from hours to 4 days at 37 °C, sometimes with constant exposure to the metal complex.

#### 3.1.3 Analytical techniques for speciation

**Fig. 5:**
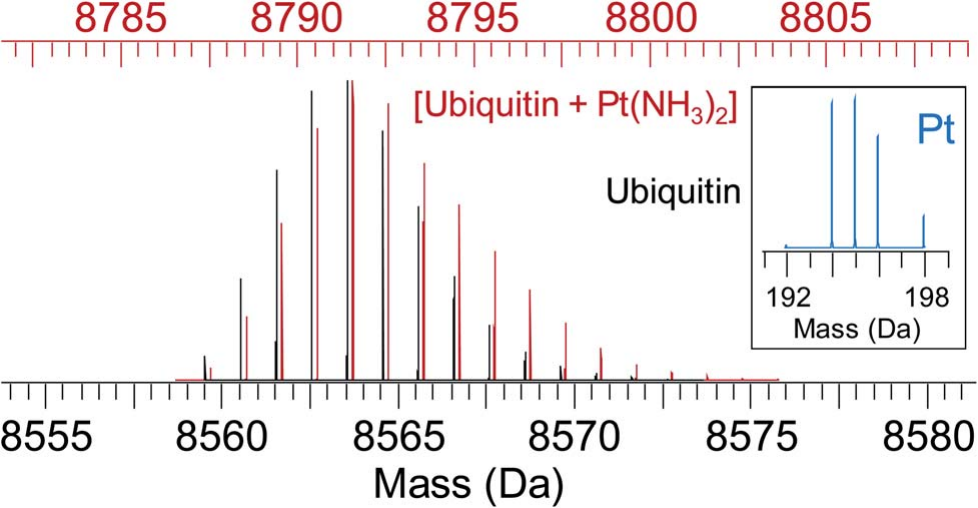
Simulated isotope pattern for 8.6 kDa protein ubiquitin (black) and [ubiquitin + Pt(NH_3_)_2_] (red, shifted by 228 Da, monoisotopic mass of Pt(NH_3_)_2_, for ease of comparison). Due to the characteristic isotope pattern of Pt (inset), the isotopic distribution of the protein–metal complex is broader and shifted to higher mass compared to the apo-protein. Automated peak-picking procedures can identify such platinated species in complex mixtures (*e.g.* cell lysates) providing insight into the binding sites for metallodrugs on proteins.^119^

Some analytical techniques have metal-specific features useful for characterising metallodrugs and their metabolites. Examples and the challenges associated with them are summarised in Table S5.† Combined application of several techniques is usually necessary. NMR, EPR, vibrational, UV-vis and CD spectroscopy, as well as MS are discussed briefly in the ESI.† An example of how protein platination can be characterised by MS is shown in Fig. 5.^119^ Such MS studies often allow identification of specific binding sites and the nature of binding to the protein, including the coordination sphere of platinum which will influence the subsequent biological behaviour of the platinated protein.

#### 3.1.4 HPLC

Reversed-phase HPLC, including nano-HPLC, can separate a variety of metal species present in biological media. However, care is needed with UV-vis quantification since extinction coefficients change with species and wavelength.^24^ Fluorescence and gamma/beta ray detection can be very sensitive while LC-MS (*vide infra*) allows direct identification of chemical species. RP-HPLC analyses often require use of acidic ion-pairing agents (*e.g.* trifluoroacetic acid) to improve peak sharpness for ionic complexes. Nonetheless, such additives lower the pH of the mobile phase, and may alter the speciation of metallodrug metabolites. For example, organo-Os^II^ hydroxido complexes (Fig. 3) stable at physiological pH exist predominantly as more labile Os–OH_2_ species under acidic HPLC conditions.^29^ Furthermore, adducts with trifluoroacetate may form, *e.g.* for various Ru^II^ and Pt^IV^ anticancer complexes.^120,121^

Size exclusion chromatography-inductively coupled plasma-mass spectrometry (SEC-ICP-MS), combines the metabolite-separating ability of SEC with element quantification by ICP-MS,^122^*e.g.* for time-dependent binding studies of Ru^II^ and Os^II^ anticancer agents to serum proteins *in vivo*.^123^

### 3.2 Anticancer agents

The platinum anticancer drugs cisplatin (**Pt1**) (FDA approval 1978), carboplatin (**Pt2**) (1986) and oxaliplatin (**Pt3**) (1996) are now used in *ca.* 50% of cancer chemotherapies. Current emphasis is on new complexes which might increase the spectrum of treatable cancers, reduce side-effects, and overcome platinum resistance.^124^ Comprehensive reviews on metallo-anticancer drugs are available.^125–128^ Selected examples of metallodrugs in clinical trials as anticancer agents are in Table 3. Although the number and variety of compounds in clinical evaluation well reflects the growing interest in this field, it must be noted that the success rate of such trials is generally quite low (*ca.* 10%) and even lower for oncology.^129^

**Table 3:** Examples of metal-based anticancer drugs that have entered clinical trials, with their stage in the clinical pipeline, administration route, treatment indication and clinical trial identifier. For structures see Fig. 2 and Section 6 of the ESI

M	Agent	Stage	Administration	Treatment	Trial**a**	Ref.
Zn/Cu	DpC (thiosemicarbazone)^b^ (Zn1†, Zn2†)	Phase I	Oral	Advanced solid tumours	NCT02688101	130
As	Darinaparsin (**As2**)	Phase I	Oral	Advanced solid tumours	NCT01139346	131
Ru	NKP1339 (**Ru9**)	Phase I	Intravenous	Colorectal carcinoma, non-small cell lung cancer and gastrointestinal neuroendocrine tumours	NCT01415297	132
Ru	TLD1433 (**Ru6**)	Phase II	Intravescical photodynamic therapy (PDT)	Photodynamic therapy for non-muscle invasive bladder cancer	NCT03945162	133
Pt	Satraplatin (**Pt6**†)	Phase I	Oral (CDMS)^c^	Prostate cancer	NCT03258320	124
				Refractory solid tumours including brain tumours	NCT01259479	134
Pt	Nanoplatin™ (NC6004)	Phase II	Infusion therapy (+pembrolizumab)	Recurrent or metastatic squamous cell carcinoma of the head and neck	NCT03771820	124 and 135
		Phase I/II	(+Gemcitabine)	Pancreatic cancer	NCT00910741	
Pt	Picoplatin (**Pt9**†)	Phase I/II	Infusion therapy (+leucovorin and 5-fluorouracil)	Colorectal cancer	NCT00478946	
		Phase I	Oral and intravenous	Solid tumours	NCT00465725	124 and 135
Pt	BTP-114 (cisplatin pro-drug) (**Pt10**)	Phase I	Intravenous	Pancreatic, ovarian, breast and prostate neoplasms (BRCA mutations)	NCT02950064	135
Pt	PT-112 (**Pt11**†)	Phase I	Intravenous	Relapsed or refractory multiple myeloma	NCT03288480	
		Phase I		Advanced solid tumours	NCT02266745	135
Pt	ProLindac™ (**Pt12**†)	Phase I	Intravenous	Unresectable and metastatic head and neck cancer	NCT00415298	135
		Phase II		Advanced ovarian cancer	EudraCT: 2010-020030-25^d^	136
Pt	Triplatin (**Pt13**)	Phase II	Intravenous	Inoperable small cell lung cancer, Advanced/metastatic adenocarcinoma of the pancreas	NCT00014547, NCT00024362	137
Au	Auranofin (**Au1**)	Phase I/II (+sirolimus)^e^	Oral	Advanced or recurrent non-small cell lung cancer or small cell lung cancer	NCT01737502	
		Phase II		Chronic lymphocytic leukaemia, small lymphocytic/prolymphocytic lymphoma	NCT01419691	135

a NCT retrieved from https://www.clinicaltrials.gov/.

b Administered as a free ligand. Active as metal complex (Zn/Cu). Complex transmetalated with Cu in lysosomes.^138^

c Combination: cabazitaxel, docetaxel, mitoxantrone or satraplatin (CDMS) plus surgery.

d From the European Union Drug Regulating Authorities Clinical Trials Database.

e Immunosuppressive.

#### 3.2.1 Cytotoxicity assays

*In vitro* evaluation relies on the assessment of the viability of 2D monolayer cultures of human cancer cells, generating dose-response curves to estimate cytotoxicity in terms of half-inhibitory concentrations (IC_50_). Some of the most commonly used cytotoxicity assays for metallodrugs are summarised in the following paragraphs. However, it should be noted that a low IC_50_ value is not necessarily predictive of successful preclinical and clinical translation of a metallodrug. Other factors to consider are off-target effects as well as bioavailability/pharmacokinetics in animals, as briefly described in Section 3.7. Importantly, novel *in vitro* models (*e.g.* spheroids and organoids) are also becoming available to better mimic *in vivo* conditions, and will be described in Section 3.2.2.

#### 

The colorimetric 3-(4,5-dimethyl-2-thiazolyl)-2,5-diphenyl-2*H*-tetrazolium bromide (MTT) assay is a gold standard. It measures the enzymatic reduction of tetrazolium to insoluble formazan crystals by dehydrogenases in cell organelles including mitochondria and endoplasmic reticulum. The assay is sensitive and can be miniaturised for high-throughput screening. However, it requires formazan solubilisation by DMSO, and formazan crystal conversion is dependent on metabolic function.^139^ It is also important to note that the reduction to formazan is catalysed by mitochondrial enzymes, potentially distorting the results of the MTT assay for complexes targeting mitochondria. For these types of complexes, including polypyridyl Ru complexes and the organo-Os^II^ azopyridines, alternative cytotoxicity tests are required.^140–142^

The Cell Counting Kit-8 tetrazolium-8-[2-(2-methoxy-4-nitrophenyl)-3-(4-nitrophenyl)-5-(2,4-disulfophenyl)-2*H*-tetrazolium] monosodium salt (CCK-8) is a colorimetric assay similar to MTT, that converts highly water-soluble tetrazolium salt WST-8 to a water-soluble orange-coloured formazan, not requiring DMSO. A limitation of colorimetric assays, is that spectroscopic interference needs to be considered when using coloured drugs.^143^

#### 

The Sulforhodamine B (SRB) assay determines protein content by binding stoichiometrically to proteins under acidic conditions, presenting no compound interference. This assay requires several wash steps and is less sensitive for poorly adherent cells.^139^ Neutral red uptake (NRU) assay determines cell viability by incorporating neutral red dye into lysosomes. For example, the NRU assay was used to determine the cytotoxic activity of two α-amino acid Schiff base-derived Ru and Pt complexes, altering lysosomal function in HepG2 cells, in accordance with MTT data.^144^ The lactate dehydrogenase (LDH) leakage assay quantifies LDH activity within the extracellular medium. NRU and MTT assays have increased cytotoxic sensitivity compared to the LDH leakage assay for hepatoma cells exposed to CdCl_2_.^145^ In particular, the MTT assay detected toxicity before the NRU and LDH assays because CdCl_2_ is likely to have an impact on the mitochondria in HepG2 cells, followed by lysosomal damage and only then LDH leakage.^145^ The resazurin reduction assay (RES) converts resazurin to fluorescent resorufin typically within the mitochondria, thus measuring metabolic activity.^139^ Similar to MTT, RES can produce false responses due to interfering chemical functional groups (*e.g.* thiols, carboxylic acids). However, MTT displayed a higher number of interferences compared to RES when tested with different concentrations of 19 potentially interfering substances (such as GSH).^146^

Clonogenic assays are useful to evaluate the long-term effects of a cytotoxic agent, by determining the number of cells able to undergo cell division and form a colony of at least 50 cells after treatment. Most commonly, crystal violet is used to stain the cells prior to counting of colonies.^147^

The type of assay used, the time of drug incubation, and the extension of cell recovery time after drug removal, can all significantly influence the results of the cytotoxicity screening. Such screens are usually carried out on unsynchronised cells, although the stage of the cell cycle can have an important effect (*vide infra*).

**Fig. 6:**
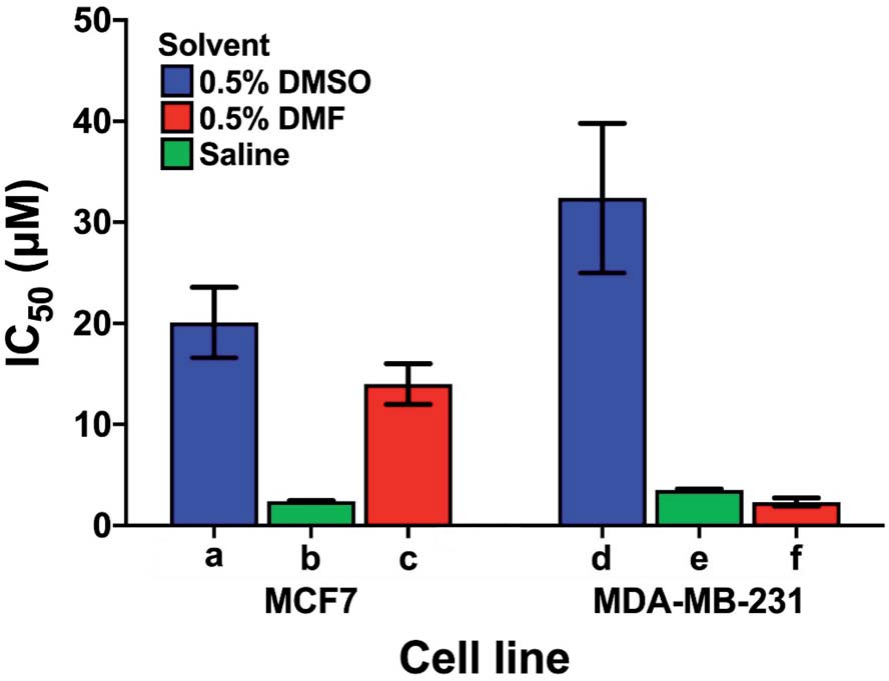
Examples of the large differences in IC_50_ values for cisplatin reported by 3 different labs (bars a, d; b, e and c, f) for the same cell lines (MCF7, human breast cancer, and MDA-MB-231, epithelial human breast cancer). The effect of the solvent used to solubilise cisplatin is highlighted; other differences in the screening conditions including type of assay, time of treatment, and composition of culture media are listed in Table S6.†

Care has to be taken when comparing cytotoxicity data reported in the literature even for the same complex in the same cell lines, as illustrated in Fig. 6. We compare the contrasting cytotoxicity data reported by different labs for the same complex (cisplatin) in the same two cell lines. The evident differences in cytotoxicity are particularly striking as cisplatin is often used as a positive control in assessment of the potency of metallodrugs. In this case, the differences are likely related to the differences in the various screening conditions as noted in Table S6† (solvent for compound dissolution, culture medium, treatment time, type of assay).

Longer treatment time usually leads to a lower IC_50_ value. The main influence on the IC_50_ values is probably due to the use of DMSO for solubilisation of cisplatin with the lowest IC_50_ values in Fig. 6 resulting from DMSO-free testing. DMSO is used as a standard solvent to ensure solubilisation of compounds for testing (even when not needed). Such effects of DMSO have been previously described by Hall *et al.*^116^ who, already 5 years ago, concluded that “practice of dissolving platinum drugs in DMSO must cease, and if the solvent is to be utilised, new platinum agents must demonstrate a lack of interaction with DMSO”.

In general, these data highlight the need for a standardised protocol to compare results from different laboratories, especially for positive controls, which ensure reliability of the data by confirming the identity and proliferation ability of cells. Such protocols should be relevant to clinical translation, which generally involves shorter drug exposure and clearance before a period without the presence of the drug. A possible standardisation that could be implemented across the field for the cisplatin control might be: solubilisation in saline, 24 h cisplatin treatment, and 72 h recovery in cisplatin-free medium prior to the chosen cytotoxicity assay.

The National Cancer Institute screen (NCI-60) determines the effect of compounds on cell viability in a panel of 60 cancer cell lines (breast, prostate, renal, ovarian, melanoma, CNS, colon, non-small lung, leukaemia). The COMPARE programme can classify unknown mechanisms of action (MoAs) for compounds by assessing the similarity between mean graph patterns of IC_50_ values.^148^

The Cancer Genome Project in the Wellcome Sanger Institute provides screening similar to NCI-60, but covers *ca.* 900 cell lines, all with known genomic DNA sequences. It produces GI_50_ values (concentration of drug for 50% of maximal inhibition of cell proliferation) to evaluate cellular drug sensitivities. Combined with the “Catalogue Of Somatic Mutations In Cancer” (COSMIC), genomic data of cancer mutations in different cell lines, it can be used to elucidate the MoAs of anticancer drugs. For example, the Sanger screen of half-sandwich organo-Ir^III^ complex ZL109 (**Ir7**†) revealed higher anticancer potency than cisplatin, and similarity to osmium azopyridine complexes and natural anticancer product piperlongumine.^149^ This suggested that the MoA involves rapid generation of ROS.^149^

#### 3.2.2 Beyond 2D cell cultures

Promising antiproliferative activity in 2D cell cultures *in vitro* often does not translate effectively into *in vivo* models. 3D multicellular tumour spheroids – spherical self-assembled aggregates of cancer cells, which allow signalling between cells in close contact - represent a more relevant model.^150^ The activity of Pt (cisplatin: **Pt1**, oxaliplatin: **Pt3** and KP1537: **Pt14**†), Ru (NKP1339: **Ru9**), Ga (KP46: **Ga2**) and La (KP772: **La1**†) compounds has been compared in 3D spheroids and 2D monolayers, plus invasion and metastasis models. The IC_50_ values of **Ga2** in all three tested cell lines were in the low micromolar range, whereas, for spheroids, the cytotoxicity was reduced by *ca.* 300-fold.^151^ This lower cytotoxicity is likely a consequence of multicellular interactions, reduced drug penetration, and possibly hypoxia in the spheroids. Overall, such data highlight the need for a combination of models for screening of compounds.^151^

*Organoids*, miniaturised organs produced from stem cells *in vitro,* are available as 3D culture models. However, there are limitations such as reproducibility as well as inefficient waste and nutrient transport. *Microfluidic organs-on-a-chip* are potential alternatives. They replicate the features of human organs by providing fine control over the microenvironment and transport *via* fluid flow, with the potential to be scaled up for high-throughput screening.^152^

### 3.3 Anti-inflammatory agents

The Au^I^ phosphine anti-inflammatory drug auranofin (**Au1**) is used in the treatment of rheumatoid arthritis. Thioredoxin reductase, with its thiol-disulfide catalytic site and selenocysteine residue, is a cellular target for auranofin which inhibits the oxidoreductase pathway implicated in inflammatory responses through the production of pro-inflammatory oxidants.^153^ However, due to severe side effects with long-term use, auranofin is not used as a first-line treatment.

Non-steroidal anti-inflammatory drugs complexed as ligands to metals may provide alternative treatments, including anticancer. This can be rationalised as inflammation is a hallmark of cancer. Examples are Cu^II^-acemetacin and Zn^II^-indomethacin which inhibit acute arterial inflammation in *in vivo* rabbit studies analysing VCAM-1 and ICAM-1 expression.^154^ Half-sandwich electron-deficient Os^II^, Ir^III^ and Ru^II^ carborane complexes (**Os7**, **Ir8**†, **Ru10**†) inhibit lipopolysaccharide-induced nitric oxide production, a known anti-inflammatory response, without cytotoxicity.^155^

*In vitro* screening techniques can be used to assess anti-inflammatory activity. Whole human blood can be stimulated with lipopolysaccharide to induce inflammation and inflammatory responses (*e.g.* INFγ, TNFα, interleukins) monitored.^156^ Assays using purified human peripheral blood mononuclear cells can also be used to screen test compounds with the same inflammatory response readout, but offer a protein-free model.^156^

### 3.4 Antimicrobial agents

The ancient Greeks and Romans discovered the antimicrobial properties of silver and used silver containers to preserve food and water.^157^ More recently silver sulfadiazine (**Ag1**) was approved as a topical cream to prevent bacterial infections in burns.^3^ Some bismuth compounds are effective in combination treatment of *Helicobacter pylori* infections.^3,158^ Tribromophenate, bismuth (Xeroform®) is used as an antibiotic in dressings, but appears to prevent bacteria entering wounds rather than have antimicrobial activity. Metallodrugs are frequently overlooked as potential antibiotics, with none currently in clinical trials. A recent study found that the metal complexes have a significantly higher hit-rate against critical bacterial and fungal pathogens compared to organic compounds.^3^**Ir9** was one of the most active compounds in the study across three strains of Gram-negative and one Gram-positive bacteria, as well as two fungi.^3^

Many important antibiotics such as penicillins, cephalosporins, and cephamycins are based on β-lactams, which can be degraded by zinc metallo-β-lactamases (MBLs) – enzymes that cause multi-drug resistance. However, bismuth (from colloidal bismuth subcitrate) can displace zinc in New Delhi metallo-β-lactamases (NDMs, a subclass of MBLs), thereby inactivating them and improving *in vivo* efficacy of β-lactams.^9^ In addition, a recent screening of a metallofragment library against selected enzyme targets showed that some ferrocene-based compounds effectively inhibit NDM-1 and warrant further exploration as scaffolds for new NDM inhibitors.^6^

Metals containing bioactive organic ligands can also act as antimicrobial agents. This approach can enhance the activity of antimicrobial ligands, especially towards drug-resistant strains. For example, Cu^II^, Co^II^ and Ni^II^ complexes containing *S*-benzyldithiocarbazate Schiff-base ligands (**M1**†) display remarkable antifungal activity against a wide spectrum of fluconazole-susceptible and -resistant *Candida albicans* isolates.^159^ Notably, these complexes are up to 1000× more active than their corresponding Schiff-base ligands and cause apoptosis *via* inhibition of ergosterol biosynthesis and membrane disruption. However, more mechanistic studies are required with these ligands to allow more detailed comparisons of their mechanisms of action.^160^ Metal-quinoline antibiotics can exhibit good antifungal activity, and 1,10-phenanthroline conjugated to Mn^II^, Ag^I^ and Cu^II^ inhibit growth of drug-resistant biofilms and planktonic fungi species that lead to the *Candida haemulonii* complex. Their mechanisms of action may include DNA and protein cleavage, mitochondrial disruption and interference with protein synthesis.^161,162^

*In vitro* screening of antibacterial and antifungal metallodrugs often follows the same methods used for the assessment of organic compounds; such as disk diffusion, gradient diffusion and broth dilution methods, and flow cytometry has been used to study cell membrane integrity, distinguishing between dead, viable and damaged cells.^163^ Some of these techniques have been subject to standardisation by the CLSI and EUCAST.^163^ However, interference caused by absorption or fluorescence of metal complexes can cause problems in these assays.

Unlike eukaryotic cytotoxicity screening, the end point of antimicrobial assays is often visual and evaluated as minimum inhibitory concentration (MIC), the lowest concentration needed to prevent visible bacterial growth, and minimum bacteriocidal concentration (MBC), the lowest concentration to achieve a biocidal effect, *e.g.* 99.5% over 18 h as colony-forming units CFU mL^−1^. Both are reported in μg mL^−1^, requiring conversion of molar units for direct comparisons.

*In vitro* screening of antiparasitic compounds is not well standardised, except in some cases, *e.g.* malaria.^164^ Screening strategies have been reviewed.^165^ Ferroquine, a ferrocene–chloroquine conjugate (**Fe2**), is active at nanomolar concentrations against chloroquine-susceptible and resistant strains of *P. falciparum*.^166^**Fe2** has recently completed a phase II clinical trial in combination with artefenomel (trial NCT03660839). Auranofin (**Au1**) has also been repurposed as an antiparasitic and is in phase IIa clinical trials for the treatment of amoebic dysentery (amoebiasis) and giardiasis, caused by *E. histolytica* and *G. lamblia*, respectively (NCT02736968). Auranofin was the most active in a high throughput screen of 910 compounds, with an EC_50_ 10-fold lower than the currently used metronidazole.^167^ Research on metal-based antiparasitics has been reviewed recently.^168^

In general, medicinal inorganic chemistry has the potential to overcome some of the major challenges in antimicrobial research including antibiotic resistance. However, research on novel metal-based antimicrobial agents does not typically extend beyond their synthesis plus MICs, MBCs and haemolysis assay evaluation and, if these are promising, the design of analogues. Whilst this research is necessary, more follow-up studies should be conducted to unravel the mechanisms of action of these metallodrugs and test their efficacy/toxicology *in vivo*. Only a more translationally-focussed approach can determine whether such compounds are indeed worthy of preclinical development, and if so, move them down the pipeline towards clinical use.

### 3.5 Antiviral agents

There is increasing interest in the antiviral activity of metal compounds, although no metallodrugs are currently used clinically. Antiviral assays fall into two distinct groups: (i) direct detection of virus population, and (ii) assessment of cell survival as a result of viral infection. Direct detection methods include plaque assays, enzyme-linked immunosorbent assays (ELISA) and quantitative real-time polymerase chain reaction (qRT-PCR). A more detailed description is in the ESI.† Methods for cell viability are discussed above (*e.g.* MTT, SRB). Selected examples of antiviral metal compounds are summarised in Table 4.

**Table 4:** Examples of antiviral metal compounds

Compound	Virus	Assay	Ref.
Polyoxometalates, Cs_2_K_4_Na[SiW_9_Nb_3_O_40_], Cs_2_K_4_Na[SiW_9_Nb_3_O_40_] (POM93)	Influenza A, influenza B, HSV-1, HSV-2, HIV-1, HBV	MTT	170
Co^III^ Schiff base complexes, CTC-96 (**Co2**, Doxovir™)	HSV-1, HSV-2	Plaque-forming units	169
Zn^II^-pyrithione (PT)	SARS-CoV-1	MTT	178
Zn^II^-cyclams	HIV-1, HIV-2	MTT	180
Pt^II^-(phen)(acyclovir/penciclovir, **Pt15**†)	HSV, CMV	MTT	171
Auranofin (**Au1**)	SARS-CoV-2	qRT-PCR, plaque-forming units	181
Ranitidine Bi^III^ citrate (based on **Bi1**)	SARS-CoV-1	qRT-PCR	173

The Schiff base complex [Co^III^(bisacetylacetatonato-ethylenediimine)(2-methylimidazole)_2_]^+^ (**Co2**, Doxovir™)^169^ reached phase II trials in 2011 for treatment of *Herpes simplex labialis* (causative agent of cold sores) and viral eye infections. **Co2** appears to prevent the entry of herpes simplex virus type 1 (HSV-1) into cells by inhibiting membrane fusion events as well as cell-to-cell spread and syncytium formation.^169^ The antiviral activity of polyoxometallate 3D frameworks has long attracted attention. For example, Cs_2_K_4_Na[SiW_9_Nb_3_O_40_] (POM93) has broad and potent *in vitro* antiviral activity against influenza A/influenza B, herpes simplex virus (HSV; Vero cells), human immunodeficiency virus (HIV-1; MT4-4 cells), and hepatitis B and C (HBV, HCV; HepG2 cells).^170^ X-ray nanotomography shows that POM93 locates on the cell surface and prevents virus entry into the cell. Pt^II^ compounds containing an aromatic diimine and antiviral guanosine-type ligands acyclovir or penciclovir (**Pt15**†)^171^ exhibit activity towards HSV and cytomegalovirus.^171^

The efficacy of Bi^III^ complexes against SARS-CoV-1 discovered in the aftermath of the 2003 outbreak of severe acute respiratory syndrome coronavirus (SARS-CoV-1), suggested that ranitidine bismuth citrate (based on **Bi1** and **L1**†)^172^ is a strong inhibitor of the ATPase activity of the viral helicase protein (IC_50_ = 0.3 μM).^173^ Förster Resonance Energy Transfer (FRET)-based assays showed that **Bi1** inhibits the DNA duplex unwinding activity (IC_50_ = 0.6 μM). Bi^III^ binds to the cysteine-rich region of the N-terminal zinc binding domain of the helicase protein. In cell culture, **Bi1** effectively inhibited virus reproduction with an EC_50_ of 5.9 μM, and a (low) cytotoxicity CC_50_ of 5 mM.^173^ qRT-PCR studies showed that **Bi1** inhibits the replicative cycle of SARS-CoV-1.^173^ These data suggest that the efficacy of **Bi1** may be due to inhibition of SARS-CoV-1 helicase, which possesses >99.5% sequence similarity to the helicase of SARS-CoV-2.^174^ Potassium bismuth citrate is currently on clinical trial for treatment of COVID-19 (Trial ChiCTR2000030398 in Wuhan), as well as the zinc ionophore hydroxychloroquine (IRCT20100228003449N28; IRCT20100228003449N29).^175^

Zinc is particularly interesting for both pathology and treatment of viral diseases. The Zn metallopeptidase angiotensin-converting enzyme 2 (ACE2) serves as the cellular entry point for both SARS-CoV-1 and SARS-CoV-2.^176,177^ The combination of Zn^II^ and the ionophore, pyrithione, can efficiently impair the replication of SARS-CoV-1 in Vero-E6 cells.^178^ Zn^II^ inhibits the RNA-synthesising activity of the multiprotein replication and transcription complex in SARS-CoV-1, and activity of viral RNA polymerase during the elongation phase of RNA synthesis.^178^ Ionophores such as pyrithione or chloroquine might facilitate cellular uptake of Zn^II^, thereby promoting this type of inhibition *in vivo*.

Human coronavirus 229E is inactivated on brass and copper-nickel surfaces,^179^ with Cu^I^ and Cu^II^ being essential for the inactivation. Superoxide and hydroxyl radical generation may play an important role in the inactivation of coronaviruses on copper alloys, whereas on the pure copper surfaces, the direct effect of copper ions is key.^179^

### 3.6 Adjuvants

Metal-based agents are also used as adjuvants to elicit an immune response. These are mainly used *in vivo*, but sometimes investigated *in vitro* for CD4^+^ T cell priming. Aluminium adjuvants have been incorporated into vaccines for over 90 years, in billions of doses administered to millions of people annually. Aluminium adjuvants are the “Gold Standard” of all adjuvants. Typically they are Al(O)OH (*e.g.* Alhydrogel®, PI 11.4) and Al(OH)_*x*_(PO_4_)_*y*_ (*e.g.* Adju-Phos®, PI 5), containing aggregates of 10–50 nm-sized particles. The mechanism of action of these adjuvants is not understood, but probably involves adsorption/binding of antigens to the surface of the nanoparticles, induction of limited necrosis, and dendritic cell activation.^182^ Adjuvants for cancer immunotherapy are being explored, including the hierarchically porous, and Cu- and Zn-containing γ-Al(O)OH mesostrands that enhance anti-tumour immunity.^183^

### 3.7 Clinical translation: ADME and safety screening

Drug candidates need to display good pharmacokinetics and bioavailability as defined by their ADME (absorption, distribution, metabolism and excretion) profiles. Only minor adverse drug reactions are deemed safe for human use. Interactions with proteins/receptors different from the desired target can result in serious effects, *e.g.* blocking of the hERG potassium channel can cause fatal arrhythmias.^184^ Pharmaceutical companies have recently proposed a panel of targets (GPCRs, ion channels, nuclear receptors and enzymes), against which new compounds should be tested.^185^ Table S7† shows examples for the different target classes. As yet, such data are scarce for most clinically approved metallodrugs (although TOOKAD® was tested for hERG inhibition as part of its EMA assessment).^186^

Genotoxicity (damage to DNA determined by *e.g.* comet and chromosome aberration assays) and mutagenicity (induced mutations, *e.g.* Ames' test, mammalian cell HPRT gene mutation assay) also need to be assessed for anticancer agents, and can be confirmed by whole-genome sequencing. Mutagenicity of cisplatin was observed in early studies^187^ and confirmed in a wide range of cell types.^188^ Cisplatin induces both base substitution mutations as well as short insertion and deletion mutations around the intrastrand crosslink sites.^189^ Notably, oxaliplatin has a different mutational profile compared to cisplatin and carboplatin.^190,191^

More information and protocols for *in vitro* assays to evaluate health-effects of drug candidates are described in Section 4 of the OECD guidelines for the testing of chemicals.^192^

## 4. Cellular mechanisms of action

Through this section we present some approaches for metallodrug target identification and study of their mechanisms of action, discussing cell death mechanisms and highlighting techniques we can use to identify cellular targets. This information is becoming increasingly important to undergo clinical translation.

### 4.1 Cell death mechanisms

Determination of death mechanisms induced by metallodrugs can sometimes indicate direct or indirect target sites. A few common cell death mechanisms are described here, and examples of specific inhibitors commonly used in screening are in Table S8.†

Apoptosis (programmed cell death) is the most common cell death mechanism for metallodrugs. Caspase proteases are cleaved and activated during apoptosis. Cleavage of caspase-3 is used as a marker. Of the two main apoptotic pathways, the extrinsic pathway is activated by a death ligand binding to a death receptor on the surface of the cell, activating initiator caspase-8. The intrinsic pathway is activated by toxins, radiation, and some chemotherapeutics which cause mitochondrial changes, such as decrease in mitochondrial membrane potential, and activate initiator caspase-9.^193^

It is generally accepted that cisplatin-induced cell death is through apoptosis, with both intrinsic and extrinsic pathways activated.^194^ However, caspase-independent cell death mechanisms can also occur in cisplatin treated cells. Carboplatin also induces apoptosis, and examples of cell death pathways induced by other metal complexes are in Table 5.

**Table 5:** Examples of metallodrug-induced cell death pathways and identification methods

M	Complex	Pathway	Method	Ref.
Ru	NKP1339 (**Ru9**)	ICD	Calreticulin cell surface expression (flow cytometry), HMGB1 release to extracellular space (ELISA)	195
Pt	Oxaliplatin (**Pt3**)	ICD	HMGB1 release to extracellular space (ELISA)	196
			Calreticulin surface expression (flow cytometry), HMGB1 motility (western blot)	197
Fe	Dinitrosyl Fe complex (**Fe3**†)	Apoptosis	Annexin-V/PI apoptosis assay (FACS)	198
			PARP, caspases 3 and 9 (immunohistochemistry) from *in vivo* tumour tissue	
Cu	Cu-TSC (**Cu2**†)	Apoptosis	Annexin V-FITC/PI apoptosis detection, caspase-3 assay, caspases 8 and 9 (qPCR)	199
Ru	RAPTA-C (**Ru12**)	Apoptosis	Cytochrome c release, procaspase-9 (western blot)	200
			Annexin-V/PI apoptosis assay (FACS)	
Pd	[Pd(bipy)(O,O′-dkt)]^+^ (**Pd2**†)	Apoptosis	Annexin-V/PI apoptosis assay (FACS), mitochondrial membrane potential using JC-1 dye (FACS)	201
Os	FY26 (**Os4**)	Apoptosis	FlowCellect Cytochrome c Kit (FACS)	202
			MitoCapture Apoptosis Detection Kit (confocal)	
Ir	Ir-TEMPO1 (**Ir10**†)	Apoptosis	Mitochondrial membrane potential using JC-10 dye (FACS)	203
Pt	Cisplatin (**Pt1**)	Apoptosis	Annexin-V/PI staining (FACS), mitochondrial membrane potential (JC-1), caspase-3	204
Pt	Carboplatin (**Pt2**)	Apoptosis	Cytochrome c release, PARP cleavage/caspases (western blot)	205
Au	[Au(L2b)PPh_3_] (**Au3**†)	Apoptosis	Mitochondrial membrane potential and caspase-3 activity (FACS)	206
Mn	Adpa-Mn (**Mn1**†)	Apoptosis and ACD	PARP-cleavage/LC-3/ATG7 expression (western blot)	207
			Annexin-V/PI apoptosis assay (FACS)	
As	As_2_O_3_ (**As3**† in aqueous solution)	ACD	MEK/ERK pathways (autophagy inhibitor studies, western blot)	208
Pt	Mono-Pt (**Pt16**†)	ACD	Anti-LC3 antibody (immunohistochemistry)	209
Ru	[(*p*-Cym)Ru(TsEn)Cl] (**Ru11**†)	Necrosis	Annexin-V/PI assay (FACS)	103
Ru	Cyclometallated Ru	Necroptosis	*p*-RIPK1/*p*-RIPK3 expression (western blot)	210

Autophagy-induced cell death (ACD) is cell death preceded by autophagy, a normal process in healthy cells for catabolism and recycling of cytoplasmic components. Large numbers of autophagic vacuoles are formed to breakdown the cytoplasm. Many components of the ACD pathway can be screened, *e.g.* formation of autophagosomes during autophagy by formation of proteins LC3-I and lipid-modified LC3-II.^211^ Some Pt complexes induce this pathway, which represents a novel mechanism compared to cisplatin.^209^ Arsenic compounds, As_2_O_3_ and NaAsO_2_ induce ACD.^212^ Autophagy activation can also occur prior to apoptosis; for example, Adpa-Mn (Mn1†) induces PARP cleavage (apoptosis) and LC-3/ATG7 (autophagy).^207^

Necrosis involves loss of the membrane integrity through self-lysis of the cell, leading to ATP depletion. It was thought to be unregulated, however, death domain receptors and toll-like receptors may activate necrosis, when caspases are absent.^213^ This more regulated form of necrosis is also known as necroptosis. The catalyst [(*p*-cym)Ru(TsEn)Cl] (**Ru11**†) allows propidium iodide (PI) to enter cells through membrane disruption without the externalisation of phosphatidylserine indicating non-apoptotic cell death.^103^ Notably, cell death by necrosis can cause a host inflammatory response.

Immunogenic cell death (ICD) is aided by the immune system. The damaged cell releases and expresses damage-associated molecular patterns (DAMPs) which recruit immune cells to the damaged cell and cause cell death through T cell activation. HMGB1 and ATP release and calreticulin relocation are key components of this pathway, examples of DAMPs which can be screened to detect ICD.^214^ Oxaliplatin (**Pt3**) induces ICD detectable by HMGB1 in patient serum and released from cell lines.^196,197^ NKP1339 (**Ru9**) induces ICD with calreticulin relocation and HMGB1 release.^195^ Importantly, the anticancer activity of oxaliplatin and related Pt^IV^ complexes in *in vivo* models often requires an intact immune system.^215^ Effectively some metal complexes may act as vaccines against cancer recurrence. The role of metal-based drugs in the anticancer immune response has been reviewed recently.^216^

### 4.2 Effect of cell cycle

Unabated deregulation of the cell cycle is a hallmark for cancer. Metallodrugs halt progression of the cell cycle at different stages, allowing time for cells to repair DNA damage.^130^ Cell cycle arrest is determined experimentally by Fluorescence-activated Cell Sorting (FACS), and PI staining identifies the cell DNA content by intercalating into DNA. Cell cycle analysis can indicate the MoA of a metal complex, whether this involves DNA binding or not.

**Fig. 7:**
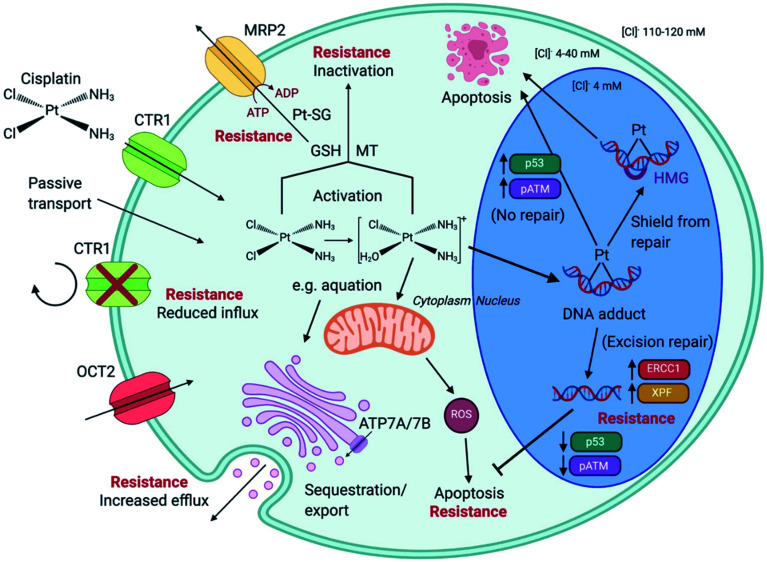
Cisplatin (CDDP) resistance in cancer cells. CDDP enters cells *via* active transport (*e.g. via* transporters CTR1 and OCT2) or by passive transport. Reduced influx and elevated efflux are forms of CDDP resistance. The ATPases ATP7A/B are located at the Golgi apparatus, in the trans-Golgi network, facilitating the sequestration and export of cisplatin within membrane vesicles that are released. Intracellular CDDP activation occurs by aquation (first stage shown). Activated CDDP can react with glutathione (GSH) to form platinum-GSH conjugates (Pt-SG) and leave the cell *via* MRP2, or interact with metallothionein (MT), contributing to CDDP tolerance. CDDP exposure generates mitochondria-dependent reactive oxygen species (ROS). CDDP enters the nucleus and forms platinum–DNA adducts. Shielding from repair, by high mobility group (HMG) protein protection, or no repair leads to apoptosis. Excision-repair and high expression of proteins such as ERCC1 and XPF, subsequently inhibit apoptosis. Augmented expression of p53 and pATM is associated G_2_/M arrest of the cell cycle and apoptosis, in contrast to lowered expression, linked to CDDP resistance. Created with https://www.BioRender.com.

**Fig. 8:**
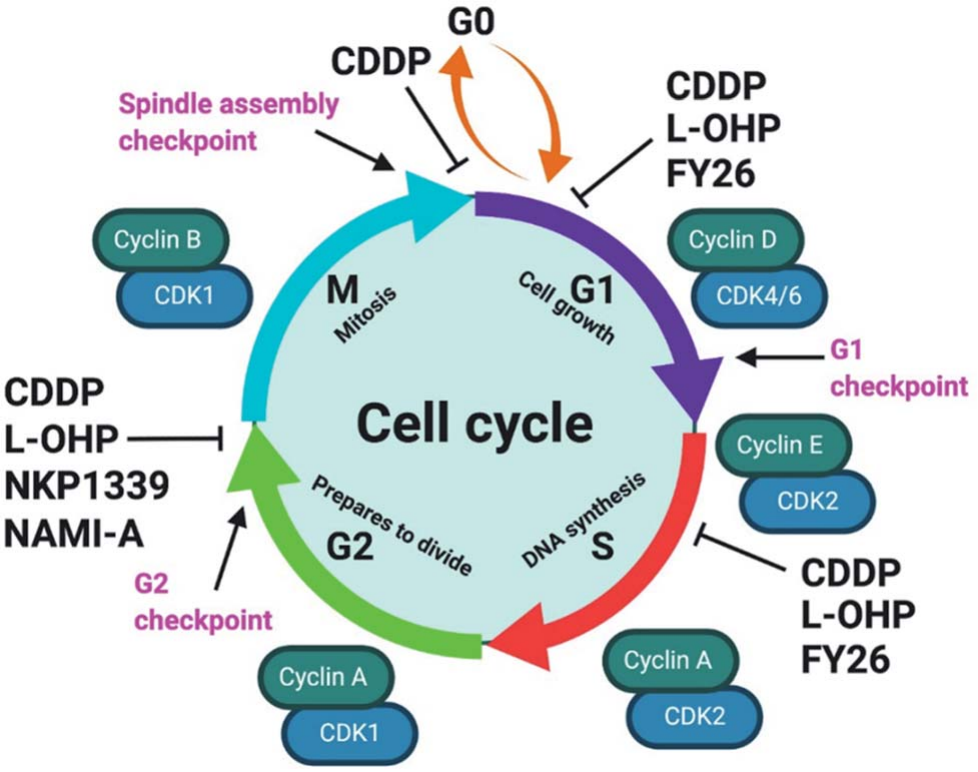
Examples of cell cycle arrest by metallodrugs. Abrogation of the cell cycle enables the initiation of apoptosis to control cell proliferation. Metal complexes can inhibit cell cycle progression at different stages which vary between different cell lines, doses, and exposure times. The cell cycle checkpoints (pink) determine progression to the next stage, and cell cycle regulators include cyclins (green) and CDKs (cyclin-dependent kinases, blue). Subpopulations of cells enter a quiescent state at the G0 phase. CDDP (cisplatin, **Pt1**); l-OHP (oxaliplatin, **Pt3**); NAMI-A (**Ru4**); NKP1339 (**Ru9**); FY26 (**Os4**). Created with https://www.BioRender.com.

In HL-60 leukaemia cells, at lower doses, cisplatin-induced DNA adduct formation arrests cells at the sub-G_1_, S or G_2_ phases. At higher doses and treatment times, cell accumulation increases within the sub-G_1_ phase, indicative of apoptosis and necrosis.^217^ Innate and acquired cisplatin resistance involves various proteins and pathways (Fig. 7). In A549 lung cancer cells, it is associated with the loss of cisplatin-induced G_2_/M phase cell cycle arrest and reduced apoptosis. Loss of activation of p53 and pATM (major responders to Pt-DNA adducts) are likely essential for cisplatin resistance.^218^ The impact of metallodrugs on cell cycle distribution differs according to the exposure time and cell line (Fig. 8). Increasing the exposure time of oxaliplatin to 72 h induces G_2_/M arrest in HeLa, MCF7 and HT29 cell lines, but A549 cells exhibit elevated G_0_/G_1_ populations.^219^

The organo-Ir^III^ complex ZL109 (**Ir7**†) stimulates cell cycle arrest in A2780 ovarian cancer cells after 24 h exposure either during replication (S-phase) or after the doubling of DNA content (G_2_-phase).^149^ Organo-Os^II^ complex FY26 (**Os4**) inhibits cell cycle progression at the G_1_-phase, prior to DNA replication, consistent with its proposed redox modulation (ROS) and not a DNA-focussed MoA.^220^ ROS can also cause DNA damage indirectly. Ru^II^ polypyridyl complexes [Ru(dmb)_2_(NMIP)](ClO_4_)_2_ (**Ru13**†) and [Ru(phen)_2_(NMIP)](ClO_4_)_2_ (**Ru14**†) arrest the cell cycle at different stages in BEL-7402 hepatocellular carcinoma cells, at G_0_/G_1_ and S phases, respectively (Table S9†).^221^ Cellular targets for Ru complexes (Table S9†) are not focussed solely on DNA interactions, and include chromatin and histones, disruption of protein–protein interactions, redox modulation, and enzyme inhibition.^222^

Diastereomeric helical “flexicate” [Fe_2_L_3_^1a^]Cl (**Fe4**†) complexes arrest HCT-116 p53^+/+^ cells at G_2_/M. In contrast, [Fe_2_L_3_^2a–b^]Cl_4_ (**Fe5**†) complexes show no significant G_2_/M population increase, indicating an alternative binding target and MoA.^223^

### 4.3 Chronopharmacology

The circadian timing system (CTS) rhythmically controls cellular and physiological functions, such as xenobiotic detoxification and metabolism, over a 24 hour period. The CTS regulates apoptosis, DNA repair and transitions of the cell cycle. The optimal circadian timing of anticancer drug administration can be determined by the CTS.^224^

GSH plays a critical role in cisplatin toxicity. Buthionine sulfoximine, which inhibits GSH biosynthesis and induces a 12 h rather than 24 h rhythm, results in potentiated cisplatin toxicity useful for clinical optimisation.^225^ Wild-type mice have better tolerance towards cisplatin in the evening than in the morning, while *Per1/2*^*−/−*^ (clock gene) mice show no clear difference. Nucleotide excision repair appears to remove cisplatin-DNA adducts in a clock-controlled manner.^226^

The circadian expression of organic cation transporter 2 (OCT2, encoded by *SLC22A2*), involved in cisplatin renal excretion, is closely associated with time-dependent alterations in cisplatin-induced nephrotoxicity.^227^ Cisplatin-resistant cells upregulate ATF4 (direct target of clock) and the expression of clock correlates with cisplatin sensitivity. The clock and ATF4 transcription system could mediate multidrug resistance *via* the GSH-dependent redox system.^228^

The overexpression of *Bmal1* (clock gene) leads to an increased sensitivity (inhibition of proliferation, apoptosis and cell cycle arrest) towards oxaliplatin both *in vitro* and *in vivo*.^229^

**Fig. 9:**
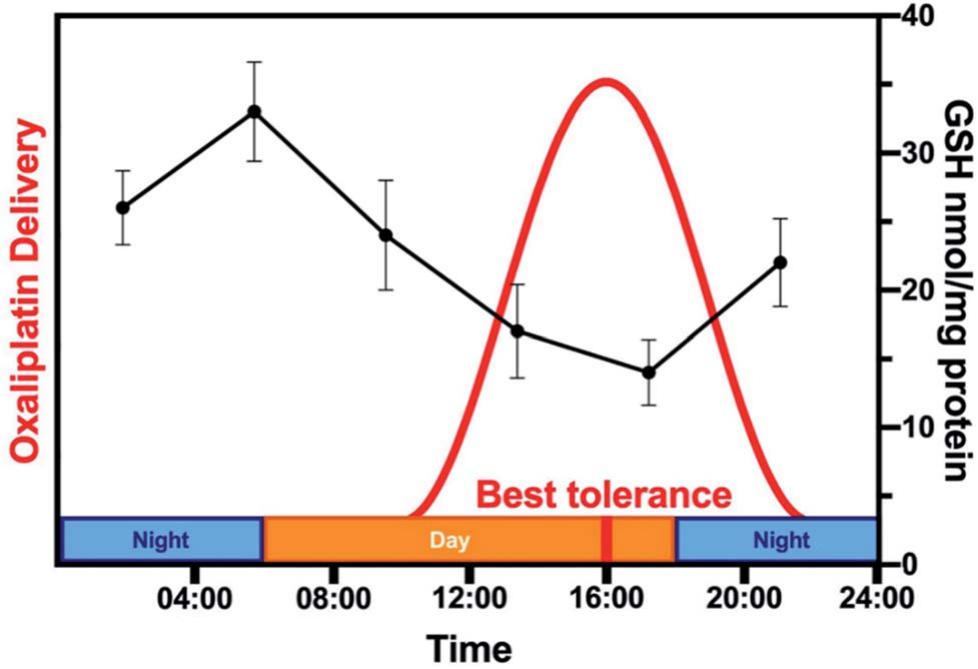
Relationship between circadian rhythm and chemotherapy. The rhythm of the GSH content of mouse liver (black, based on data from ref. 225) is compared to the schedule of administration of oxaliplatin (**Pt3**) in a typical chronomodulated chemotherapy. Patients show best tolerance at 16:00 (red). Oxaliplatin is detoxified by conjugation to glutathione, hence, tolerance is linked directly to GSH levels (note the 12 h phase-shift between the highest GSH levels in mice and best tolerance in humans due to nocturnality of mice).

Analysing all chronopharmacological aspects of a novel compound is costly and time-consuming, but potentially important for clinical use of metallodrugs. Two randomised phase III trials reported that 278 metastatic colorectal cancer patients receiving chrono-modulated delivery of oxaliplatin exhibited decreased treatment side effects with a 5-fold difference and twice the anti-tumour efficacy.^224^ The relationship between circadian rhythm and chemotherapy is illustrated for oxaliplatin in Fig. 9.^225^ Furthermore, organo-osmium complex FY26 (**Os4**) exhibits temperature rhythm-driven *in vitro* and *in vivo* chronotolerance. The tolerability of **Os4** is highest near the lowest point of the circadian temperature cycle.^230^

### 4.4 Target identification

Identification of intracellular molecules and biochemical pathways as targets for new therapeutic agents is a key step in medicinal chemistry. However, this aspect has been somewhat neglected in the development of metallodrugs for which DNA is often assumed as the natural target, based on cisplatin (**Pt1**) activity.^15^ The techniques now available, briefly reviewed in the following paragraphs, allow new and different targets to be discovered for both novel and clinically established metallodrugs.

#### 4.4.1 CRISPR screening

Genome-wide CRISPR screening techniques include options to knockout, silence, or activate genes. Current applications to metallodrugs focus on the original Cas9 version, which causes DNA double-strand breaks and result in frame-shift mutations to knockout a gene.

Limitations of genome-wide CRISPR screening include high levels of off-target effects and large background signals using a pooled screen.^231^ The problem with large background signals is amplified by multi-targeted metal complexes, which necessitates use of a high concentration of complex and multiple replicates. There are only 4 reported genome-wide CRISPR studies of resistance to cisplatin or any metal complex. The role of DNA repair protein MSH2 in cisplatin-mediated cell death in bladder cancer cells has been identified,^232^ and the transcription factor ZNF587B, and extracellular sulfatase SULF1, were found to contribute to cisplatin-mediated cell death in two ovarian carcinoma cell lines.^233^ Both negative and positive selection genome-wide screens have identified pathways involved in cisplatin resistance and cisplatin sensitivity in human melanoma cells.^234^ A combination study with cisplatin and the folate antimetabolite pemetrexed demonstrated that loss of WEE1 sensitises cells to this treatment regime and is a potential therapeutic target.^235^

#### 4.4.2 RNAi screening

RNA interference (RNAi) is a natural cellular mechanism, which protects genomes by inhibiting gene expression or translation. RNAi causes the degradation of mRNA, so preventing the production of the protein, resulting in knocking down gene expression. Drugs with similar mechanisms of action are likely to elicit similar shRNA-dependent responses. Though different techniques can be used (*e.g.* siRNA, shRNA or miRNA), the principle remains the same. A piece of selected dsRNA is constructed and cut by the enzyme Dicer to create siRNA, which then becomes part of an RNA-induced silencing complex that binds to the target mRNA and degrades it.^236^ RNAi-mediated suppression of cell death regulators in mammalian cells can affect the cellular response to drugs. In this way, the effect of genes known or predicted to have roles in cell death can be investigated. Examples of reported RNAi studies for cisplatin are in Table S10.†

Eight such genes have been investigated by Bruno *et al.* for their role in the cytotoxicity of cisplatin, carboplatin and oxaliplatin.^237^ Unlike cisplatin and carboplatin, oxaliplatin (at an LD_80_ dose) did not induce a DNA-damage response, but instead induces ribosome biogenesis stress. Further work is needed to determine whether specific proteins are targeted by oxaliplatin. Using RNAi signatures for cancer cells, an Os^VI^ nitrido complex **Os8** showed a pattern between DNA cross-linkers *e.g.* cisplatin, and ER stress inducers, *e.g.* tunicamycin, indicating that it could initiate both DNA damage and ER stress.^238^ Silencing the *ERCC-1* gene in cisplatin-resistant SGC-7901 human gastric cancer cells induces apoptosis and lowers the IC_50_ after cisplatin treatment for 48 h. ERCC-1 plays a critical role in the DNA repair pathway, removing cisplatin–DNA adducts.^239^

High throughput RNAi screening can provide a relatively rapid screening method for genome-scale functional studies.^240^ An RNAi screen for 2400 genes identified genes engaged in DNA repair-enhanced sensitivity towards cisplatin and other anticancer drugs. BRCA pathway genes (*BRCA1*, *BARD1*, *BRCA2,* and *RAD51*) were the most prominent in enhancing sensitivity towards cisplatin.^241^ Interference in selected genes may result in an improved outcome through a synergic effect alongside chemotherapy. For example, silencing E6 or E7 protein of human papillomavirus, which degrades tumour-suppressor p53 or retinoblastoma proteins in combination with cisplatin results in induced apoptosis *in vitro* and inhibition of tumour growth *in vivo*.^242^ Silencing survivin and bcl-2 in cisplatin-resistant A549 cells results in increased sensitivity towards cisplatin, *in vitro* and *in vivo*.^243^

### 4.5 Pathway analysis by omics techniques

Metallomics provides a powerful systems biology approach for elucidating the biomolecules, processes, and pathways involved in the dynamic speciation of metal ions and their quantitative distribution in cells and tissues.^244^Table 6 gives examples of metallodrugs investigated by multi-omics techniques. Genomics and transcriptomics speed up the study of DNA and RNA (especially mRNA), and can provide insights into biological processes including DNA replication, nucleotide excision repair and transcription. Microarrays are widely applied to fast screening, without complicated sample preparation. Time-series whole-transcriptome sequencing shows that FY26 (**Os4**) rapidly activates oxidative stress reponse pathways and induces a shift of cellular energetics from glycolysis to oxidative phosphorylation in A2780 ovarian cancer cells.^220^

**Table 6:** Examples of metallodrugs investigated by multi-omics techniques

M	Complex	Method	Mechanism of action	Ref.
As	ZIO-101 (**As2**)	Metalloproteomics by GE-ICP-MS	Binds to and disrupts dimerisation of H3.3	248
Ru	NAMI-A (**Ru4**)	Proteomics by MALDI-TOF or LC-ESI/MS–MS	NAMI-A and RAPTA-T similar mechanism; NIT2, TMK, HINT1 and PFD3 up-regulated, POLE3 down-regulated	250
Ru	Plecstatin-1 (**Ru15**†)	Proteomics by LC-MS/MS	Targets plectin	251
Ru	RAPTA-C (**Ru12**)	Proteomics by shotgun and pull-down	Influences cytokines midkine, pleiotrophin, fibroblast growth factor-binding protein 3, FAM32A	252
Os	FY26 (**Os4**)	RNA transcriptomics and proteomics by reverse-phase protein arrays	Shifts cellular metabolism to oxidative phosphorylation, up-regulates ATM, p53 and p21	220
Pt	Cisplatin (**Pt1**)	Genomics by damage-seq, XR-seq, RNA-seq	Per1 upregulated. Transcription-coupled repair and global repair high in liver	253
		Proteomics by nanoLC-MS/MS	HMGB1 binds to cisplatin cross-linked ODN specifically	254
		Metabolomics by GC-MS, LC-MS	27 potential biomarkers defined related to cisplatin-induced nephrotoxicity	255
Au	[Au(TPP)]Cl (**Au4**†)	Proteomics by MALDI-TOF-MS	Targets Hsp60	256
		Affinity-based proteome profiling SILAC		246

UPLC and high-resolution MS have improved sensitivity and selectivity in proteomics. Besides the species and expression level, proteomics can reveal post-translational modifications, *e.g.* ubiquitination, phosphorylation, and glycosylation. Proteomics based on LC-MS/MS identified cisplatin cross-linking at the domain I/II interface zinc binding site (His67 and His247).^245^ Chemical proteomics with click reactions and photo-crosslinking identified heat-shock protein 60 (Hsp60) as the target of gold anticancer complex [Au(TPP)]Cl (**Au4**†).^246^ Hsp60 was confirmed as a target by quantitative proteomics using stable isotope labeling with amino acids in cell culture (SILAC), as well as the cellular thermal-shift assay (CETSA).^246^

Small metabolites can be analysed by NMR, or MS with sample preseparation by GC or LC/HPLC giving orders of magnitude increase in sensitivity and selectivity. Metabolomic GC-MS profiling shows that mitochondria-targeting [Re_2_(CO)_6_(4,7-diphenyl-1,10-phenanthroline)_2_(L)](PF_6_)_2_, where L is 4,4′-azopyridine (**Re-Re1**) or 4,4′-dithiodipyridine (**Re-Re2**†), induces oxidative stress by down-regulating 5-oxoproline to (20–31%) and decreases GSH levels to (34–40%) affecting glutathione metabolic pathways in HeLa cells.^247^ GE-ICP-MS revealed Cys in histone H3.3 as a target for anticancer drug *S*-dimethylarsino-glutathione (**As2**), but not for the drug As_2_O_3_ (**As3**† in aqueous solution, Trisenox) in leukaemic cells, disrupting dimer formation and facilitating denaturation of nucleosomes.^248^

A new deep learning bioinformatics approach based on database integration has revealed disease-associated metal-relevant site mutations in the first and second coordination spheres of metalloproteins.^249^ For example, the mutation Arg174Gly in the second coordination sphere of the Zn(Cys)_3_His site in tumour suppressor p53 is a disease-associated mutation.^249^

## 5. *In situ* analysis and imaging of metallodrugs

**Fig. 10:**
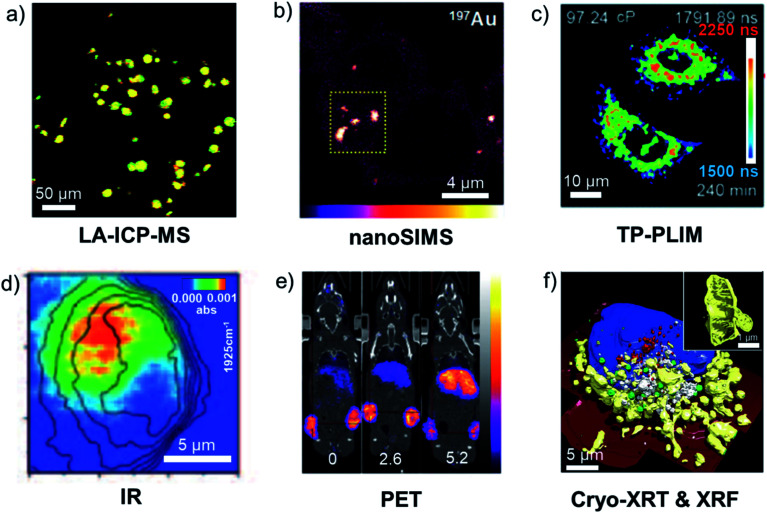
Examples of metallodrug mapping by the indicated techniques. (a) LA-ICP-MS image of ^193^Ir (red) and ^238^U (green) in HeLa cells (overlay = yellow, scale bar 50 μm).^258^ (b) nanoSIMS image of ^197^Au in A2780 cells treated with AuMesoIX (**Au2**, scale bar: 4 μm).^55^ (c) Mitochondrial viscosity in **Ir3**-treated A549 cells determined by two-photon phosphorescence lifetime imaging (scale bar: 10 μm).^259^ (d) IR image of a Re–CO complex at 1925 cm^−1^ in MDA-MB-231 cells (scale bar 5 μm).^260^ (e) Biodistribution in a mouse of positron-emitting ^89^Zr-antibody conjugated to a drug *via* a Pt complex at different drug : antibody ratios (0, 2.6 and 5.2).^261^ (f) 3D-reconstructed image of **Ir3**-treated MCF7 cells, illustrating morphology of suborganelle structures (scale bars: 5 μm and 1 μm) blue: nucleus, red: dense vesicles, green: lipid droplets, white: vacuoles, yellow: mitochondria, dark red: cell membrane.^262^ Illustrations are adapted from the references given.

In this section we discuss some of the methods commonly used to detect and visualise metallodrugs in cells and tissues and the specific advantages and challenges of each technique. A graphical overview of the techniques discussed is presented in Fig. 10.^257^

A major challenge when analysing metallodrugs in cells and tissues is preserving their distribution in organelles, and speciation (oxidation state and coordination environment). Metal speciation is often dynamic in nature and can change with *e.g.* time and temperature in both intact and preserved samples. Therefore, the possible effects of all handling procedures on the metallodrug and its metabolites need to be considered.

*Cell preservation*. Methods include cryopreservation, chemical fixation, dehydration, resin-embedding, and sectioning (extended reading, S3 ESI†). Common chemical fixatives include aldehydes (*e.g.* glutaraldehyde) or alcohols (*e.g.* ethanol) which rapidly preserve cells by crosslinking or precipitation of proteins. Cryopreservation avoids chemical treatment by rapidly preserving cells at low temperatures (*e.g.* 103 K, liquid ethane) in a near-native state. However, great care has to be taken to vitrify rather than crystallise water during freezing by extremely rapid cooling. With care and temperature control, freeze-drying can be used to dehydrate cells after cryopreservation. Fixed samples embedded in paraffin wax or frozen samples embedded in “optimal cutting temperature compound” (OCT, polymeric mixture) can be sectioned on a microtome, although thin sections can be difficult to handle.

### 5.1 Inductively coupled plasma (ICP) methods

ICP-MS can quantitate a wide range of elements in biological solutions down to ppb range, *ca.* 100-fold more sensitive than Optical Emission Spectroscopy (ICP-OES).^263^ It is a destructive technique, and only provides information on elemental content. This limitation can be partially addressed by ‘hyphenated’ experiments such as SEC-ICP-MS (*vide supra*).^123^ The preparation of cell and tissue samples for ICP-MS analysis requires thermal digestion and the use of concentrated acids/bases to ensure complete sample homogenisation.^263^ Tougher tissues often require H_2_O_2_ and/or the use of pressurised microwave heating.^264^ A compatible solvent matrix for the analyte must be selected to avoid interferences and loss of elements (*e.g.* Os as volatile OsO_4_ under acidic conditions).^265^

Fractionation methods can be used to analyse distinct cellular components (*e.g.* membranes, nuclei, cytoplasm, mitochondria), extracted by lysis of cells followed by ultracentrifugation to separate components that differ in size/weight.^266^

#### 5.1.1 Single-cell ICP-MS

Whereas cell digestion ICP-MS studies provide only an average measurement of an element in a sample of cells, single-cell ICP-MS can determine the time-resolved elemental composition of individual cells amongst a population. For example, the uptake of Pt by A2780 cisplatin-sensitive ovarian cancer cells treated with 10 μM cisplatin (*ca.* 4 × 10^6^ Pt atoms per cell) was found to be greater than for resistant cells (*ca.* 3 × 10^6^ Pt atoms per cell).^267^

### 5.2 Imaging mass spectrometry

Mass spectrometry combined with ablation techniques can provide information on the spatial distribution of metals and fragments of metallodrugs.

The spatial distribution of Ru in the liver, kidneys, muscles and tumours of CT26 tumour-bearing mice treated with anticancer organoruthenium plecstatin-1 (**Ru15**†) determined by laser ablation-ICP-MS (LA-ICP-MS) is similar to its isosteric Os analogue, in good agreement with ICP-MS data after digestion.^268^

Nanoscale secondary ion mass spectrometry (nanoSIMS) offers spatial resolution (down to *ca.* 50 nm) an order of magnitude better than photon-based ablation.^269^ Ablation and ionisation in SIMS occur in the same process, and molecular information is typically not preserved. The energy of the primary ion beam is equivalent to X-ray wavelengths (0.06–8 nm). Molecular information can be better preserved in SIMS by use of larger primary ions, *e.g.* large Ar clusters.^270^ High ion doses allow depth profiling.^270^

NanoSIMS studies of 300 nm-thick sections of resin-embedded colon cancer cells treated with ^15^N-labelled cisplatin (10–150 μM) suggest partial dissociation of Pt–N bonds in at least the nucleoli, based on the Pt/N stoichiometry, and co-localisation of Pt with S and P (detection of ^194^Pt, ^34^S and ^15^N signals), consistent with the affinity of Pt^II^ for S-rich proteins and DNA.^271^

Similarly, correlative nanoSIMS and electron microscopy imaging of Au^III^ mesoporphyrin IX dimethyl ester (**Au2**) in A2780 cells suggests that this anticancer complex attacks sulfur-rich proteins in mitochondria (^197^Au in regions with high ^32^S/^12^C^14^N ratios).^55^ There is much scope for isotopic labelling of both metals and ligands in such studies.

### 5.3 Luminescence imaging

The cellular localisation of luminescent metal complexes with triplet metal-to-ligand charge-transfer (^3^MLCT) excited states can be monitored by fluorescence microscopy.^272^ Such emissions are often tuneable and intense, and have large Stokes shifts, long emission lifetimes, high signal-to-noise ratios, and high photostability.^272,273^ For example, Ru^II^ complex TLD-1433 (**Ru6**) exhibits red luminescence (580–680 nm) from an emissive ^3^MLCT state and generates ROS with a large ^1^O_2_ quantum yield (*Φ*(Δ) ∼ 0.99 in acetonitrile) from a intraligand ^3^IL state when excited by green light (530 nm).^274^

Weakly- or non-luminescent metal complexes can be conjugated to fluorescent organic dyes or more-strongly luminescent metal complexes to allow their cellular imaging.^78,275^ The non-emissive dinuclear (bpy)_2_Ru^II^-Eu^III^(cyclen) (**Eu-Ru1**†) complex releases DNA-targeting Ru^II^ and an emissive Eu^III^(cyclen) species upon irradiation at 488 nm.^276^ The red emission of Eu^III^(cyclen) species can be detected in cancer cells with 350 nm one-photon or 700 nm two-photon near-infrared excitation.

Two-photon imaging provides increased light penetration depth and nano-localisation.^277,278^ Mitochondria-targeting Ru^II^ polypyridyl PS **Ru6**† for example, showed cellular fluorescence with both one- and two-photon excitation, but the deeper spheroid penetration achieved with two-photon excitation highlights the increased depth advantage of the two-photon method.^279^

The problem of background autofluorescence of cells can be overcome using fluorescence/phosphorescence lifetime imaging (FLIM/PLIM), improving signal-to-noise ratios and providing quantitative information.^272^ Cyclometalated Ir^III^ anticancer complex **Ir11**† exhibits a specific response to the local viscosity, allowing mitochondrial viscosity and heterogeneity to be monitored in real-time using two-photon-PLIM.^259^

#### 5.3.1 Theranostic luminescent complexes

These can serve the dual purpose of *in situ* prodrug activation (therapy) and activation-induced luminescence for imaging of cellular localisation (diagnosis). Metal centres can be designed to quench fluorescence after redox activation, usually by the conversion of a diamagnetic to a paramagnetic metal (*e.g.* Ru^II^ to Ru^III^).^280^ When theranostic Pt^IV^ prodrugs with a conjugated aggregation-induced emission (AIE) group are reduced to active Pt^II^ drugs, the AIE group is released and fluoresces, allowing direct measurement of metallodrug activation and oxidation state.^281^

### 5.4 Vibrational spectroscopy

Carbonyl stretches for metal carbonyl complexes inside cells are readily observable in the 1800–2200 cm^−1^ region, which is relatively free of peaks from biomolecules. For example, the uptake and distribution of [(Cp)Re(CO)_3_] conjugated to a hydroxytamoxifen-like structure (**Re2**†) in breast cancer cells has been determined by photothermally-induced resonance (PTIR, 20–50 nm resolution) with an AFM coupled to a tuneable pulsed infrared laser. **Re2**† was found to be localised in the nucleus of whole cells at a 10 μM dose.^260^ Photoinduced CO releasing molecules (PhotoCORMs) and photoinduced NO releasing molecules (PhotoNORMs) are of therapeutic interest.^282,283^ 3D Raman microscopy of living HT29 colon cancer cells treated with 2 mM of the photoactivated anticancer complex [Mn(tpm)(CO)_3_]Cl (**Mn2**) for 3 h, revealed accumulation in the nuclear membrane and nucleus.^284^

### 5.5 Radionuclide labelling

Introduction of a radioactive label into a pharmaceutical agent should cause minimal change to its molecular structure. Radiometals are often attached to a targeting vector *via* a bifunctional metal chelator. By changing a therapeutic (*e.g.* α or β emitter) to an imaging (*e.g.* γ or positron emitter for SPECT and PET imaging, respectively) radiometal, the pharmacokinetics of a metal-based radiotherapeutic can be imaged.

A “theranostic pair” consists of a combination of two agents with the same/similar structure, one therapeutic and the other diagnostic. For example, the PET agent ^68^Ga-DOTA-PSMA (**M2**†) has been used to monitor the response of the beta emitter ^177^Lu-DOTA-PSMA and the alpha emitter ^225^Ac-DOTA-PSMA in prostate cancer patients.^285^

Non-radioactive metallodrugs can also be radiolabelled, either at the ligand or at the metal centre, to understand their mechanism of action or biodistribution. For example, cancer cells treated with half-sandwich osmium complex FY26 (**Os4**) radiolabelled with ^131^I release the iodide ligand, probably after reaction with intracellular GSH.^29^

An example of metal radiolabelling is [^195m^Pt]cisplatin, the biodistribution of which was investigated in humans in the early 1970s.^286^ [^195m^Pt]carboplatin distribution has also been investigated in preclinical models.^287^ Recent improvements in imaging equipment and production of ^195m^Pt radiolabelled complexes have seen a resurgence of such studies with a focus on preclinical imaging.^261,288^

[Cu(ATSM)] (**Cu3**) was developed first as a hypoxia imaging agent, [^64^Cu][Cu(ATSM)],^289^ but later recognised as a SOD-1 agonist.^290^ [Cu(ATSM)] is now in clinical trials for motor neuron diseases such as Parkinson's disease (completed, GDCT0292870) and Amyotrophic Lateral Sclerosis (ongoing, GDCT0344437).

Many other radiometals could be used to elucidate the fate of metallodrugs *in vitro* and *in vivo*. The chemistries of ^68^Ga and ^89^Zr are well developed and should be considered for monitoring the biodistribution of drug candidates containing such metals. In addition, other radiometals, including ^52^Mn and ^62^Zn/^63^Zn, are becoming increasingly available for preclinical investigations.

### 5.6 Synchrotron X-rays

Synchrotron radiation covers a wide range of the electromagnetic spectrum: from infrared (1.24 meV), to soft and hard (>*ca.* 10 keV) X-rays. This broad range enables X-ray Fluorescence (XRF); X-ray Absorption Spectroscopy (XAS), X-ray Tomography (XRT) and Infrared Microspectroscopy.

**Fig. 11:**
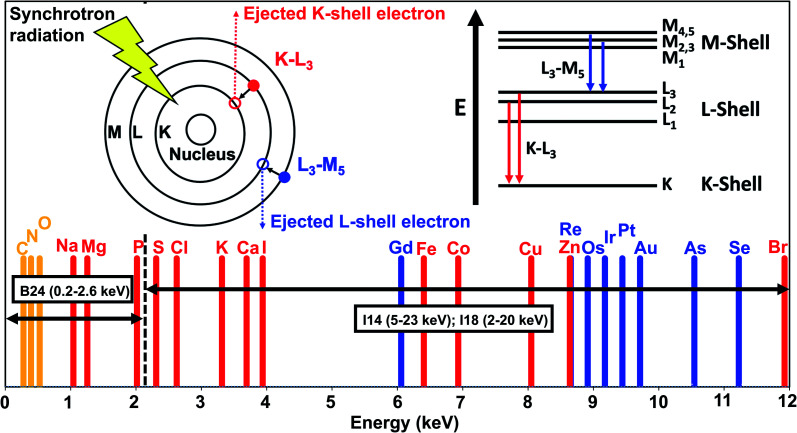
Characteristic K-L_3_ (2p_3/2_^−1^ → 1 s^−1^) and L_3_-M_5_ (3d_5/2_^−1^ → 2p_3/2_^−1^) X-ray fluorescence emissions. K-L_3_ for elements exploited in cryo-soft-X-ray tomography are in yellow, abundant endogenous elements in red; L_3_-M_5_ of exogenous elements are blue. Additional elements are reported in Table S11† B24 and I14 refer to beamlines at the Diamond synchrotron (www.diamond.ac.uk).

Absorption of a high-energy X-ray photon by an atom causes ejection of an inner-shell electron. When an electron in a higher-energy orbital subsequently drops down to occupy this hole, a photon is emitted, resulting in XRF. The energy of the emitted photons is characteristic of specific elements, allowing simultaneous detection and quantification at trace levels as low as 100 ppb, down to 50 nm resolution.^291,292^ Endogenous elements such as Zn, P, or S exhibit K-L_3_ emissions (2p_3/2_^−1^ → 1s^−1^) in the energy range 2–10 keV. In contrast, the K-L_3_ emissions of exogenous heavy elements (*e.g.* Pt, Ir, Os) are significantly higher in energy (>60 keV); thus, typically their L_3_-M_5_ emissions (3d_5/2_^−1^ → 2p_3/2_^−1^) are monitored (Fig. 11).

Synchrotron-XRF can provide insights into the biological distribution and localisation of metallodrugs, both *in vitro* (chemically-fixed, sectioned, or freeze-dried cells) and *ex vivo* (tumour spheroids and xenograft models).^292,293^ 3D XRF-computed tomography has been used to investigate the penetration of Pt complexes (**Pt17**†, **Pt18**†) in tumour spheroids, demonstrating >150 μm depth.^294^ The localisation of Os in small elliptical cytoplasmic compartments of A2780 cancer cells treated with FY26 (**Os4**) implicated mitochondria as cellular targets.^295^ Non-metal exogenous elements (*e.g.* Br or I, low natural abundance) can also be employed to monitor the intracellular fate of metallodrugs by XRF, as demonstrated for Br-labeled Pt agents (**Pt19**†, **Pt20**†) and I-labelled rhenium (**Re3**†) complexes *in vitro*,^296,297^ through a dual-mapping approach.

#### 5.6.1 X-ray Absorption Spectroscopy (XAS)

Absorption of an X-ray photon induces the transition of a core electron (typically 1s, 2s or 2p) into a vacant state according to Fermi's Golden Rule. X-ray Absorption Near Edge Structure (XANES) focuses on the region within 50–100 eV of the absorption edge.^292^ It is highly sensitive to the absorbing environment of the metal, and considers multiple scattering contributions. XANES can inform on the oxidation state, electronic structure and the coordination environment, but is typically limited to concentrated samples, and can be destructive and time-consuming.^292^ XANES studies can provide insights into the speciation and stability of metallodrugs in biological media. Pt XANES (L_3_ = 11.57 keV) has demonstrated the stability of Pt^IV^ prodrugs in human blood serum but rapid in-cell activation.^298^ Similarly, Ru XANES (K = 22.1 keV) has revealed potential interactions between Ru^III^ drugs **Ru4** (NAMI-A) and **Ru5** (KP1019)† and the sulfur, amine and carboxylate groups of proteins in biological media.^299^

Extended X-ray Absorption Fine Structure (EXAFS) is observed *ca.* 50 to 1000 eV above the absorption edge, providing a unique fingerprint region of the XAS spectrum. EXAFS usually considers only single scattering contributions and is highly sensitive to neighbouring environments, thus providing information on metal–ligand distances (up to *ca.* 4 Å) and the number and type of neighbouring atoms.^292^ However, EXAFS requires relatively high concentrations, which hinders its use for *in cellula*, *ex vivo* or *in vivo* analysis. Instead, EXAFS is commonly used to study metallodrug interactions with relevant biomolecules, *e.g.* to demonstrate the catalytic activation of **Os4***via* the formation of GSH adducts.^300^

#### 5.6.2 Soft X-ray Tomography (XRT)

‘Soft’ X-rays are less penetrating than hard X-rays and more strongly absorbed, providing greater absorption contrast for lighter, biological materials (C, N, O). Also, a soft X-ray beam is easier to focus to spot sizes down to a few tens of nm.^301^ At this extremely high spatial resolution, depth of field is often <1 μm, requiring very thin samples and careful optimisation of sample position. Measurements are performed *in vacuo* since soft X-rays are efficiently absorbed by air and water. Conversely, attenuation of hard X-rays by air is negligible. An important consideration with X-ray spectroscopy is beam damage. The photon dose needs to be carefully controlled, using known standards and/or repeat measurements.^302^

Cryo-XRT allows 3D morphological and ultrastructural analysis of intact frozen-hydrated cells or tissues,^303^ using energies between the K-edges of C (284 eV) and O (543 eV). Organic materials absorb strongly in this energy range, the ‘water window’, whilst the X-ray beam is minimally attenuated by ice. Consequently, carbon-dense materials (*e.g.* mitochondria or lipid droplets), provide natural contrast against water from vitrified ice on the samples and in the cytoplasm down to a spatial resolution of 30–40 nm.^304^ Cryo-XRT has been used to probe the effectiveness of cisplatin in the presence of chelating tricine, which causes significant cell damage and numerous apoptotic bodies.^305^ Similarly, cryo-XRT revealed significant mitochondrial damage in photoirradiated cancer cells treated with a cyclometallated Ir^III^ photosensitiser **Ir12**.^306^ Cryo-XRT can be coupled to other techniques such as structured illumination microscopy (cryo-SIM)^304^ or hard-XRF, which enables the direct detection of metal complexes overlaid with the 3D subcellular morphology. Notably, cryo-XRT and cryo-XRF of (near-native) cancer cells treated with a potent half-sandwich Ir^III^ complex (**Ir3**) have revealed the unambiguous mitochondrial localisation of Ir (L_3_-M_5_ = 9.18 keV).^262^ The capability of obtaining native-state “snapshots” of biological systems emphasizes the notable advantage of cryo-XRT over conventional microscopy techniques.

#### 5.6.3 Synchrotron-IR microspectroscopy

Live, single-cell analysis can be carried out, providing morphological and biochemical insights of biological specimens in their native states,^307^ for example, live fibroblasts.^308^ Synchrotron-IR is also invaluable for monitoring metallodrugs, by either directly probing the metallodrug modality, or the drug-induced cellular changes.^309^

## 6. Conclusions and future outlook

This perspective is being written during the COVID-19 pandemic, which has emphasised more than ever the urgent need for new medicines. Medicinal inorganic chemistry can play a major role in such development, providing tools complementary to those of organic medicinal chemistry. Inorganic compounds and metals in particular, can introduce novel, unique mechanisms of action, which cannot be duplicated by organic compounds.

A comprehensive survey of current literature on the development of metal complexes as antiviral drugs by Bergamini *et al.* has just been published, asking the question “What is holding back the development of antiviral metallodrugs”.^310^

SARS-CoV-2 enters cells *via* the ACE2 receptor.^177^ ACE2 is a zinc carboxypeptidase (which cleaves C-terminal Phe from angiotensin II, DRVYIHPF).^176^ Dexamethasone, a corticosteroid used for treating critically ill COVID-19 patients, induces the Cys-rich protein metallothionein which binds clusters of Zn^II^ and Cu^I^ ions.^311^ Potassium Bi^III^ citrate is in phase III clinical trials (ChiCTR2000030398) for treating the disease. Bismuth can displace Zn^II^ in proteins vital for the life cycle of coronaviruses.^173^ Importantly, Sun *et al.* have now shown that the antiulcer drug ranitidine bismuth citrate (RBC) exhibits low cytotoxicity and protects SARS-CoV-2-infected cells with a high selectivity and suppresses SARS-CoV-2 replication, leading to decreased viral loads in both upper and lower respiratory tracts, and relieves virus-associated pneumonia in a golden Syrian hamster model.^312^

Moreover, a high selenium status appears to improve the cure rate for COVID-19 patients.^313^ Vaccines for SARS-CoV-2 are being developed. The potency of many vaccines is enhanced by Al^III^ adjuvants, but the molecular basis for this is not understood.

In the field of antimicrobials, the hit-rate for metal compounds in some screens is 10× higher towards ESKAPE pathogens than that for organic compounds.^3^ It has long been known that metal surfaces (*e.g.* Cu and its alloys, Ag) are potently antiseptic, but the molecular basis is poorly understood. High-throughput screening has revealed the potent antiparasitic activity of the antiarthritic gold drug auranofin.^167^

The success of platinum complexes, the drugs used in about 50% of all cancer chemotherapies, raises the question as to why more metallodrugs are not being approved for clinical use. It is remarkable that 71 kinase inhibitors, a single class of organic drugs, are clinically approved (with >100 others in late-stage development),^314^ about twice as many as the total number of currently approved metallodrugs (Fig. 1).

We have focussed on *in vitro* screens since they are a convenient discovery platform for medicinal chemists, and on the need to identify metallodrug target sites, one of the key areas for advancement towards more clinical approvals. However, new procedures are needed for metallodrugs compared to screens for organic drugs. Metallodrugs are usually prodrugs. They can undergo ligand exchange and redox reactions on their way to or at target sites. Furthermore, they are often multitargeting. Cell culture media contain a complicated mixture of components, sometimes ill-defined and variable, which can influence the behaviour of metallodrugs. The synthesised metal complex may be readily transformed into new species in the culture media, or even in the DMSO often used as a universal solubilising medium. However, with modern physical techniques, the nature of the species in culture media and even in cells can now be investigated. In addition, the instability of metallodrugs can be turned to an advantage if their activation (*e.g.* by hydrolysis, metal or ligand-based redox reactions, external forces such as photo-irradiation) can be controlled.

The need to select carefully the screening procedure for metallodrugs is highlighted by the anticancer activity of oxaliplatin and some other Pt^IV^ complexes in solid tumour models being dependent on an intact immune system. Moreover, the efficacy and toxicity of oxaliplatin is dependent on stages of the cell cycle and the body clock (day or night administration).

The unique properties of alpha-, beta- or gamma-emitting radioactive metals, their increased availability for therapy, diagnosis and tracing, and reliable methods for their chelation and conjugation to vectors delivering them to receptors and other targets is an exciting area of current research. Omics (genomics, proteomics, and metallomics) technologies now allow exploration of the multiple pathways of metallodrug actions. They have revealed, for example, that despite the structural similarity of the clinical drugs cisplatin, carboplatin and oxaliplatin, the latter targets ribosome biogenesis and not genomic DNA.^237^ The combined application of hyphenated chromatography-mass spectrometry methods can reveal specific target sites for metallodrugs on proteins and other targets. Use of the wide range of energies of electromagnetic radiation from gamma and X-rays, to UV-vis and IR to micro- and radio-waves, allows the characteristic properties of metal complexes to be used for detection and imaging of metallodrugs and their metabolites in intact biological samples, including frozen cells.

The uniqueness of the roles of metals in drugs has been illustrated here with examples especially from anticancer and antimicrobial agents. Although not discussed here, salts of essential metals such as Na, K, Mg and Ca (*e.g.* see Table 2 in ref. 2) are used daily on a massive scale in a variety of medicines and clearly can also not be replaced by organic compounds. In addition to metallodrugs, metal ions may be involved in the mechanisms of action for organic drugs, since these often contain up to 10 H-bond acceptors (a Lipinski rule) which will also act as metal-binding sites.

Metallodrugs are challenging to design and develop. However, their different and unique properties provide invaluable opportunities for the discovery of drugs with novel mechanisms of action, able to address the ever-complex medical challenges that our world is facing.

## Conflicts of interest

There are no conflicts to declare.

## Supplementary Material

SC-011-D0SC04082G-s001
