# Supplementary material for: Metallodrugs are unique: opportunities and challenges of discovery and development
Source: Chem Sci. 2020 Nov 12;11(48):12888–917. doi: 10.1039/d0sc04082g (PMC8163330; doi:10.1039/d0sc04082g)
Supplement: SC-011-D0SC04082G-s001 [file SC-011-D0SC04082G-s001.pdf]

Electronic Supplementary Material (ESI) for Chemical Science.
This journal is © The Royal Society of Chemistry 2020

Supplementary information

Metallodrugs are unique: opportunities and challenges of discovery and development

The authors

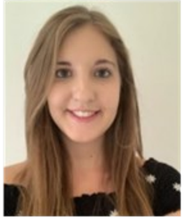
Elizabeth Anthony

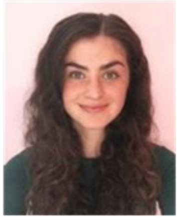
Elizabeth Bolitho

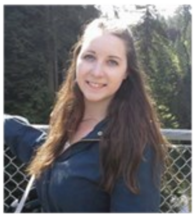
Hannah Bridgewater

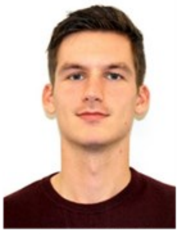
Oliver Carter

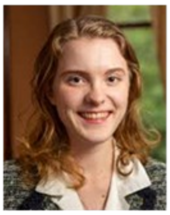
Jane Donnelly

Elizabeth Anthony *graduated from the University of Warwick with an MBio in Biological Sciences in 2019. She is a PhD student at the University of Birmingham on a placement at Warwick working on clock control of metallodrug transport in cancer cells.*

Elizabeth Bolitho *graduated from King's College London in 2017 with a BSc in Chemistry with Biomedicine. She has just completed her PhD on elucidation of the mechanisms of action of metallodrugs using (Diamond) synchrotron techniques.*

Hannah Bridgewater *obtained a BSc in Biochemistry from the University of Kent in 2016. She completed her PhD at the University of Warwick on the application of CRISPR techniques to study metallodrug activity in 2020. She is now a research fellow at Warwick studying viral-associated cancers.*

Oliver Carter *obtained a MChem in 2018 from the University of Warwick. He is an EPSRC Molecular and Analytical Sciences CDT PhD CASE student at the University of Warwick collaborating on metallodrug design with the Golden Keys High-tech Materials Co., Ltd (Guian, China).*

Jane Donnelly *graduated from Georgetown University in 2019 with a B.S. in Biochemistry. She is a Fulbright scholar in the Department of Engineering at the University of Warwick studying the role of transition metals in Alzheimer's Disease.*

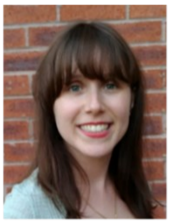
Cinzia Imberti

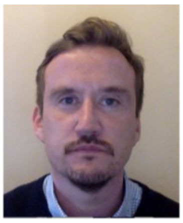
Edward Lant

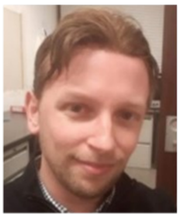
Frederik Lermyte

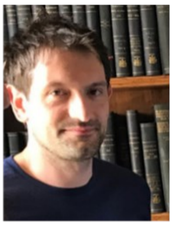
Russell Needham

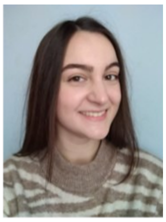
Marta Palau

Cinzia Imberti *obtained her MSci at Padua University in 2012, followed by an MRes and PhD in radionuclide imaging at King's College London in 2018. She Is a Sir Henry Wellcome postdoctoral fellow at the University of Warwick studying photoactivatable anticancer agents, and a member of RSC Dalton Division Council.*

Edward Lant *graduated with a BSc in Natural Sciences from the Open University in 2019 after 16 years as an industrial engineer. He is an EPSRC Molecular and Analytical Sciences CDT PhD CASE student at the University of Warwick working on ultra-high resolution MS studies on metallodrugs in collaboration with Bruker.*

Frederik Lermyte *obtained his PhD at the University of Antwerp in 2016. Since 2017 he has been a Research Fellow at Warwick using FTICR-MS and synchrotron spectromicroscopy to study the role of metals in neurodegeneration. He is currently an Assistant Professor at Department of Chemistry, Technical University of Darmstadt, Germany.*

Russell Needham *graduated from the University of Nottingham in 2007 with an MSci in Chemistry, and from Warwick in 2016 with a PhD on organo-osmium anticancer complexes. He is now an Anglo-American research fellow at Warwick working on metallodrug development.*

Marta Palau *graduated from the University of Barcelona in 2016 with a BSc in Chemistry and in 2017 with an MSc in Organic Chemistry. After working in industry, she was awarded a Warwick Chancellor's Scholarship in 2019 to study for a PhD in medicinal inorganic chemistry.*

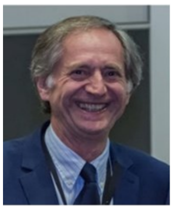
Peter Sadler
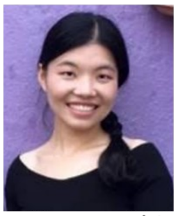
Huayun Shi
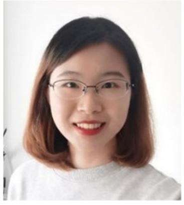
Fang-Xin Wang
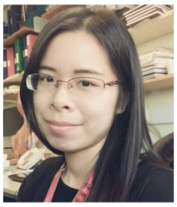
Wen-Ying Zhang
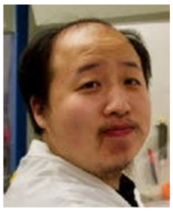
Zijin Zhang

Peter Sadler, *a professor of chemistry at the University of Warwick, works closely with his co-authors on the design and mechanism of action of metallodrugs. He is a Fellow of the Royal Societies of London and Edinburgh, the European Academy of Sciences, Honorary Fellow of the Chemical Research Society of India and Chinese Chemical Society, and an EPSRC RISE Fellow.*

Huayun Shi *obtained her BSc from Sun Yat-sen University in 2015 and was awarded a Warwick Chancellor's Scholarship to study for her PhD on photoactivated platinum complexes, which she completed in 2019. She has recently researched the market potential for metallodrugs as an ICURe early career researcher and is now an Anglo-American research fellow working on photoactivatable metallodrugs.*

Fang-Xin Wang *graduated from Sun Yat-sen University with a BSc in Chemistry in 2015, where she is now studying for her PhD on transition metal anticancer drugs. Currently she is a China Scholarship Council (CSC) visiting scholar at the University of Warwick.*

Wen-Ying Zhang *obtained her BSc in chemistry from Henan University in 2013 and Master's degree from Fudan University in 2016. She was awarded a Warwick Chancellor's Scholarship for PhD study on organometallic anticancer complexes, which she has just completed.*

Zijin Zhang *graduated from Nanjing University with a BSc in Chemistry in 2014 and a Master's in 2017. He is now studying for a PhD at the University of Warwick PhD on metallodrug chronopharmacology supported by the Chinese Scholarship Council.*

Contents

1. Abbreviations

2. Tables cited in main text

Table S1. Medicines containing a metal in their active pharmaceutical ingredient

Table S2. Composition of RPMI-1640 cancer cells growth medium

Table S3. Composition of foetal bovine serum (FBS)

Table S4. Common media used for *in vitro* screening of antimicrobial activity

Table S5. Some analytical methods for metallodrug speciation

Table S6. Cisplatin cytotoxicity assays

Table S7. Examples of target classes for identification of off-target effects

Table S8. Cell death pathway inhibitors

Table S9. Different stages of cell cycle arrest for ruthenium anticancer complexes

Table S10. RNAi studies for cisplatin

Table S11. Characteristic electron-binding energies and X-ray emissions of elements

3. Extended Reading

S1. Viral Assays

S2. Analytical techniques for metallodrugs speciation

S3. Methods for cell preservation

Table S12 NMR parameters for commonly used nuclei in metallodrug speciation

Table S13 EPR parameters of some common metallodrug paramagnetic nuclei

Table S14 Summary of cell and tissue preservation methods

Figure S1. Simulated fine isotope pattern for the (A+1) peak of the OCS^+ ion

4. References

5. Additional examples of recent relevant literature

6. Metallodrug structures

1. Abbreviations

Acac: acetylacetonate

ACE2: angiotensin-converting enzyme 2

AFM: atomic force microscopy

AIE: aggregation-induced emission

Azpy: phenylazopyridine

BCG: Bacillus Calmette-Guerin

Bipy: bipyridine

BSA: bovine serum albumin

CC_{50}: half maximal cytotoxic concentration

CCK8: cell counting kit-8

CCS: collision cross-section

CD: circular dichroism

CDDP: cisplatin

CDK: cyclin-dependent kinases

CDMS: combination: Cabazitaxel, Docetaxel, Mitoxantrone or Satraplatin

CETSA: cellular thermal-shift assay

CFU: colony-forming unit

CLSI: clinical & laboratory standards institute

CNS: central nervous system

COSMIC: catalogue of somatic mutations in cancer

COVID-19: coronavirus disease 2019

Cp: cyclopentadienyl

Cp*: pentamethylcyclopentadienyl

Cp^x: substituted pentamethylcyclopentadienyl

CRISPR: clustered regularly interspaced short palindromic repeats

CTS: circadian timing system

Dmb: 4′-dimethyl-2,2′-bipyridine

DMEM: Dulbecco's modified Eagle medium

DOTA: dodecane tetraacetic acid

EC_{50}: half maximal effective concentration

ELISA: enzyme-linked immunosorbent assay

En: ethylenediamine

ENDOR: electron-nucleus doublé resonance

EMA: European Medicines Agency

EPR: electron paramagnetic resonance

ER: endoplasmatic reticulum

ESEEM: electron spin echo envelope modulation

ES(P)R: electron spin (paramagnetic) resonance

ESKAPE: panel of bacteria pathogens: *Enterococcus faecium, Staphylococcus aureus; Klebsiella pneumonia, Acinetobacter baumannii, Pseudomonas aeruginosa, Enterobacter spp*

EUCAST: European committee on antimicrobial susceptibility testing

EXAFS: extended X-ray absorption fine structure

FACS: fluorescence-activated cell sorting

FBS/FCS: foetal bovine serum/ foetal calf serum

Fc: ferrocene

FDA: Food and Drug Administration

FLIM/PLIM: fluorescence/phosphorescence lifetime imaging

FRET: Förster resonance energy transfer

FT-ICR: Fourier transform-ion cyclotron resonance

FT-IR: Fourier transform infrared

GI_{50}: half-maximal growth inhibition

GMP: guanosine monophosphate

GPCR: G-protein-coupled receptor

GSH: glutathione

GSSG: glutathione disulfide

GSK-3: glycogen synthase kinase 3

HBV: hepatitis B virus

HCV: hepatitis C virus

HIV: human immunodeficiency virus

HSV: herpes simplex virus

HSA: human serum albumin

HSQC: heteronuclear single quantum coherence

HYSCORE: hyperfine sublevel correlation

IC_{50}: half-maximal inhibitory concentration

ICD: immunogenic cell death

ICP: inductively coupled plasma

ICP-MS: inductively coupled-plasma-mass spectrometry

ICP-OES: inductively coupled plasma-optical emission spectroscopy

IL: intraligand

IM: ion mobility

Impy: iminoazopyridine

LA-ICP-MS: laser ablation-inductively coupled plasma-mass spectrometry

LC-MS: liquid chromatography-mass spectrometry

LD_{80}: lethal dose at 80%

LDH: lactate dehydrogenase

LMCT: ligand-to-metal charge transfer

L-OHP: oxaliplatin

MoAs: mechanisms of action

MBC: minimum bacteriocidal concentration

MBLs: metallo-β-lactamases

NDM: New Delhi metallo-β-lactamases

MBP: metal binding pharmacophore

MEM: modified Eagle medium

MIC: minimum inhibitory concentration

miRNA: micro ribonucleic acid

ML: metal-ligand

MLCT: metal-to-ligand charge transfer

mRNA: messenger ribonucleic acid

MTT: 3-(4,5-dimethyl-2-thiazolyl)- 2,5-diphenyl-2*H*-tetrazolium bromide

MV: molecular volume

NAD: nicotinamide adenine dinucleotide

NADP: nicotinamide adenine dinucleotide phosphate

NAMI: new anti-tumour metástasis inhibitor

NCI-60: National Cancer Institute– 60 cell line screen

NCT: National Clinical Trial

NIR: near infrared

NMIP: 2′-(2″-nitro-3″,4″-methylenedioxyphenyl)imidazo[4′,5′-f] -phenanthroline

NRU: neutral red uptake

OCT2: organic cation transporter 2

OECD: Organisation for Economic Co-operation and Development

PACT: photoactivated chemotherapy

PDT: photodynamic therapy

PET: positron emission tomography

PFU: plaque-forming unit

Phen: 1,10-phenanthroline

Phpy: phenylpyridine

PhotoCORM: photoinduced CO releasing molecule

PhotoNORM: photoinduced NO releasing molecule

PI: propidium iodide

Pico: picolinate

Pq: 3-phenylisoquinoline

PS: photosensitiser

PSMA: prostate-specific membrane antigen

PTIR: photothermally-induced resonance

PTT: photothermal therapy

Py: pyridine

qRT-PCR: quantitative real time polymerase chain reaction

RES: resazurin reduction assay

RNAi: ribonucleic acid interference

ROS: reactive oxygen species

RP-HPLC: reverse phase-high pressure liquid chromatography

RPMI: Roswell Park Memorial Institute

SARS-CoV: severe acute respiratory syndrome coronavirus

SEC-ICP-MS: size exclusion chromatography-inductively coupled plasma-mass spectrometry

SILAC: stable isotope labeling with amino acids in cell culture

SIM: structured-illumination microscopy

SIMS: secondary-ion mass spectrometry

shRNA: short hairpin ribonucleic acid

siRNA: small interfering ribonucleic acid

SOD-1: superoxide dismutase 1

SPECT: single-photon emission computed tomography

SXT: soft x-ray tomography

SRB: sulforhodamine B

TPP: *meso*-tetraphenylporphyrin

TP-PLIM: two-photon phosphorescence lifetime imaging

TM: transition metal

TMC: tetra-*N*-methylated cyclam

Ttpy: 4'-(p-tolyl)-2,2':6',2''-terpyridine

Tth: *Thermos thermophilus*

UPLC: ultra performance liquid chromatography

XANES: X-ray absorption near edge structure

XAS: X-ray absorption spectroscopy

XPDT: X-ray photodynamic therapy

XRF: X-ray fluorescence

XRT: X-ray tomography

2. Tables cited in main text

Table S1. List of medicines containing a metal in their active pharmaceutical ingredient.

M	Agent	Approved by[a]	Approval	Status	Indication
Li	Lithium carbonate/citrate	FDA/EU	1970	Marketed	Bipolar disorder
Mg	Magnesium sulfate	FDA/EU	1994	Marketed	Seizures, eclampsia, pre-eclampsia
Al	Sucralfate	FDA/Italy	1981	Marketed	Gastrointestinal ulcers
Al	Mixed aluminium salts	FDA/EU	unclear	Marketed	Adjuvant in vaccines
K	Potassium chloride	FDA/EU	1920's	Marketed	Hypokalaemia
Ca	Calcium acetate	FDA/EU	2007	Marketed	Hyperphosphatemia
Ca	Patiromer sorbitex calcium[b]	FDA/EU	2015	Marketed	Hyperkalaemia
Fe	Sodium nitroprusside	FDA/Italy	1974	Marketed	Hypertension, heart failure
Fe	Saccharated ferric oxide[c]	FDA/EU	1949	Marketed	Iron deficiency anaemia
Fe	Ferric citrate	FDA/EU	2014	Marketed	Hyperphosphatemia
Fe	Ferric pyrophosphate citrate	FDA	2019	Marketed	Iron deficiency anaemia
Fe	Prussian blue insoluble	FDA/EU	1997	Marketed	Heavy metal poisoning
Co	Hydroxocobalamin	FDA/EMA	1996	Marketed	Cyanide poisoning, pernicious anaemia
Co	Cyanocobalamin	FDA/Italy	1989	Marketed	Constipation, pernicious anaemia
Ga	Gallium nitrate	FDA	1991	Discontinued in 2016	Cancer-related hypercalcemia
As	Salvarsan	N/A	1910	Discontinued[d]	Syphilis
As	Melarsoprol	France	1997	Marketed	Sleeping sickness
As	Arsenic trioxide	FDA/EMA	2000	Marketed	Acute promyelocytic leukaemia
Sr	Strontium ranelate	EU	2004	Marketed	Osteoporosis
^{89}Sr	Strontium-89 chloride	FDA/Spain	1993	Marketed	Pain from bone metastasis
^{90}Y	Ibritumomab tiuxetan	FDA/EMA	2002	Marketed	Follicular, B-cell non-Hodgkin, non-Hodgkin and mantle cell lymphomas
Zr	Sodium zirconium cyclosilicate	EMA/FDA	2018	Marketed	Hyperkalaemia
Pd	Tookad soluble	EMA	2015	Marketed	Prostate cancer
Ag	Silver sulfadiazine	FDA/EU	1975	Marketed	Infections of burns and bedsores
Sb	Glucantime	France/Spain	1952	Marketed	Leishmaniasis
Sb	Pentostam	UK	2011	Marketed	Leishmaniasis
^{153}Sm	Sm-153 lexidronam	FDA/EMA	1997	Marketed	Cancer pain, ostealgia (bone pain)
^{177}Lu	Lu-dotatate	FDA/EMA	2017	Marketed	Neuroendocrine tumours
Pt	Cisplatin	FDA/EU	1978	Marketed	Testicular, cervical, ovarian, head and neck cancers; bladder, squamous/non-small cell lung cancer
Pt	Carboplatin	FDA/EU	1986	Marketed	Ovarian and small-cell lung cancers
Pt	Oxaliplatin	FDA/EU	1996	Marketed	Colon/ metastatic colorectal cancers
Au	Aurothioglucose	FDA/ EU	1940s	Discontinued	Rheumatoid arthritis
Au	Aurotioprol	France	1998	Discontinued	Rheumatoid arthritis
Au	Myocrisin	EMA/ EU	1969	Discontinued in 2019	Rheumatoid arthritis
Au	Auranofin	FDA/EU	1986	Marketed	Rheumatoid arthritis
Hg	Merbromin	FDA/EU	unclear	Discontinued in 1998	Antiseptic, antisepsis
Bi	Xeroform dressing	FDA/EMA	1970s	Marketed as device	Occlusive dressing for burns
Bi	Bismuth subcitrate potassium	FDA/EU	2006[e]	Marketed	*H. pylori* infections; duodenal and peptic ulcers
Bi	Bismuth subgallate	FDA/ Germany	unclear	Marketed	Internal deodorant (e.g. for hemorroids)
Bi	Bibrocathol	EU	2012	Marketed	Bacterial conjunctivitis, blepharitis
Bi	Bismuth subsalicylate	FDA	2018	Marketed	*H. pylori* infections; duodenal ulcer
^{222}Ra	Radium dichloride	FDA/EMA	2013	Marketed	Bone metastasis, hormone refractory prostate cancer

Medicines in which the metal only represents the counterion are not included. No diagnostic agents, food supplements or agents contained in anaesthetic or implants have been included. Different formulations of a single API are counted as one entry. For the sake of simplicity, the list is limited to agents approved in the US or EU market as retrieved from GlobalData Drug Database in May 2020. [a] Agents listed as approved by EU are approved in specific EU countries, whereas agents listed as approved by EMA are approved in EU. [b] And other Ca-releasing polymers. [c] And other Fe complexes with sugar substrates. [d] Before establishment of FDA. [e] As combination - used previously. Different bismuth-based agents are used for the same indications in other part of the world, for example colloid bismuth pectin (approved in China since 1998).

Table S2. Composition of RPMI-1640 cancer cell growth medium.[a]

Component	Concentration (mg/L)	Category
Phenol red	5.0	pH indicator
Glutathione (reduced)	1.0	Redox balance
D-Glucose	2000.0	Sugar
Calcium nitrate	100.0	Salt
Magnesium sulfate	48.84	Salt
Potassium chloride	400.0	Salt
Sodium bicarbonate	2000.0	Salt
Sodium chloride	6000.0	Salt
Sodium phosphate dibasic	800.0	Salt
L-Arginine	200.0	Amino acid
L-Asparagine	50.0	Amino acid
L-Aspartic acid	20.0	Amino acid
L-Cystine 2HCl	65.0	Amino acid
L-Glutamic acid	20.0	Amino acid
L-Glutamine[b]	300.0	Amino acid
Glycine	10.0	Amino acid
L-Histidine	15.0	Amino acid
L-Hydroxyproline	20.0	Amino acid
L-Isoleucine	50.0	Amino acid
L-Leucine	50.0	Amino acid
L-Lysine hydrochloride	40.0	Amino acid
L-Methionine	15.0	Amino acid
L-Phenylalanine	15.0	Amino acid
L-Proline	20.0	Amino acid
L-Serine	30.0	Amino acid
L-Threonine	20.0	Amino acid
L-Tryptophan	5.0	Amino acid
L-Tyrosine disodium salt dehydrate	29.0	Amino acid
L-Valine	20.0	Amino acid
4-Aminobenzoic acid	1.0	Vitamin
D-Biotin (Vitamin H)	0.2	Vitamin
D-Calcium pantothenate (Vitamin B5)	0.25	Vitamin
Choline chloride	3.0	Vitamin
Cyanocobalamin (Vitamin B12)	0.005	Vitamin
Folic acid	1.0	Vitamin
Myo-inositol	35.0	Vitamin(-like)
Niacinamide	1.0	Vitamin
Pyridoxine hydrochloride	1.0	Vitamin
Riboflavin (Vitamin B2)	0.2	Vitamin
Thiamine hydrochloride (Vitamin B1)	1.0	Vitamin

[a] https://www.thermofisher.com/es/es/home/technical-resources/media-formulation.114.html Accessed 9th July 2020. [b] Not present in all RPMI-1640 culture media.

Table S3. Composition of foetal bovine serum (FBS) used for eukaryotic cell growth medium.

Components[a]	Origin		
	USA	Australia	Brazil
**Enzymes**			
Alkaline phosphatase	280 U/L	227 U/L	N.D.[b]
Serum alanine transaminase	N.D.	10 U/L	N.D.
Serum aspartate transaminase	N.D.	52 U/L	N.D.
γ-glutamyl transpeptidase	4 U/L	4 U/L	N.D.
Lactic dehydrogenase	699 U/L	588 U/L	N.D.
Serum glutamyl oxalactate transaminase	77 U/L	N.D.	N.D.
**Inorganic species**			
Bicarbonate	14 mmol/L (14 mM)	15 mmol/L (15 mM)	N.D.
Calcium	144 mg/L (3.6 mM)	141 mg/L (3.5 mM)	N.D.
Chloride	96 mM	95 mM	N.D.
Phosphorus	104 mg/L (3.35 mM)	98 mg/L (3.16 mM)	N.D.
Potassium	13.5 mM	11 mM	N.D.
Sodium	140 mM	138 mM	N.D.
Iron	N.D.	2.05 mg/L (36.6 μM)	N.D.
**Metabolites**			
Bilirubin	2 mg/L (3.42 μM)	2 mg/L (3.42 μM)	N.D.
Blood urea nitrogen	140 mg/L (5 mM)	160 mg/L (5.7 mM)	N.D.
Cholesterol	250 mg/L (646 μM)	350 mg/L (904 μM)	N.D.
Creatinine	29 mg/L (257 μM)	24 mg/L (212 μM)	N.D.
Glucose	1510 mg/L (8.39 mM)	750 mg/L (4.17 mM)	N.D.
Triglycerides (TG)	750 mg/L	590 mg/L	N.D.
Uric acid	33 mg/L (194 μM)	15 mg/L (89 μM)	N.D.
Estradiol	27.8 ng/L	413 ng/L	N.D.
Progesterone	< 0.05 μg/L	< 0.06 μg/L	N.D.
Testosterone	< 0.1 μg/L (< 347 pM)	< 0.1 μg/L (< 347 pM)	N.D.
Thyroxine (T4)	101 μg/L (130 μM)	113 μg/L (145 nM)	N.D.
**Proteins**			
High density lipoproteins	100 mg/L	100 mg/L	N.D.
Low density lipoproteins	N.D.	130 mg/L	N.D.
Hemoglobin	11.6 mg% (180 nmol%)	102 mg/L (1.58 μM)	135.5 mg/L (2.1 μM)
Albumin	N.D.	17.4 g/L (261 μM)	22.1 g/L (332 μM)
α-globulin	N.D.	13.8 g/L (148 μM)	16.7 g/L (180 μM)
β-globulin	N.D.	4.3 g/L	3.8 g/L
γ-globulin	N.D.	70 mg/L	15.64 mg/L
Insulin	8.21 mIU/L	5.67 mIU/L	N.D.
Transferrin	1.95 g/L (24.4 μM)	N.D.	N.D.
Total protein	37 g/L	35.5 g/L	42.6 g/L
**Other**			
Endotoxins[c]	< 0.05 EU/mL	< 0.5 EU/mL	0.9 EU/mL

[a] Components in FBS are listed from the certificate of analysis provided by Gibco: https://www.thermofisher.com/order/catalog/product/26140079#/26140079. Accessed 30th June 2020. Concentrations may vary slightly between batches. [b] Components listed as N.D. are not presented in the certificate of analysis. [c] Concentration of endotoxins varies in FBS from different origins. For stem cell lines and sensitive cell lines with gene knockdown, FBS from the USA and Australia with less endotoxins is recommended.

Table S4. Common media used for *in vitro* screening of antimicrobial activity.

Growth medium	Screening method[1]	Component	Concentration	Purpose/Category	Ref
Mueller Hinton Agar (MHA)	Disk diffusion on bacteria, yeast and moulds<br>Agar dilution on bacteria	Beef extract<br>Casein hydrolysate<br>Starch<br><br><br><br>Agar	2.0 g/L<br>17.5 g/L<br>1.5 g/L<br><br><br><br>17.0 g/L	Nutrients<br>Nutrients<br>Colloid, nutrient. It absorbs toxins released from bacteria to avoid interference with antibiotics<br>Solidifying agent	[a]
Mueller Hinton Broth (MHB)	Broth microdilution, broth microdilution, time-kill test on bacteria	Same composition as MHA without agar		-	[b]
Roswell Park Memorial Institute medium (RPMI 1640)	Broth microdilution, broth macrodilution on yeast and moulds	See **Table S2**			
Lysogeny broth (LB)		Tryptone<br>Yeast extract<br>Sodium chloride	10 g/L<br>5 g/L<br>10 g/L (LB-Miller)<br>5 g/L (LB-Lennox)<br>0.5 g/L (LB-Luria)	Source of amino acids<br>Nutrients<br>Provides appropriate osmotic conditions	[c]

[a] Sigma-Aldrich's 70191 Mueller Hinton Agar datasheet. Accessed 1st May 2020. [b] Sigma-Aldrich's 70192 Mueller Hinton Broth datasheet. Accessed 1st May 2020. [c] https://www.sigmaaldrich.com/life-science/molecular-biology/molecular-biology-products.html?TablePage=9618513 Accessed 2nd May 2020.

Table S5. Some analytical methods for metallodrug speciation.

Technique	Advantages	Disadvantages	Information
NMR	Mixtures without separation<br>Quantitative	Insensitive nuclei<br>Mainly diamagnetic compounds	Equilibrium and kinetic parameters<br>Stereochemistry<br>Metabolomics
EPR	Metals, and organic radicals<br>Solutions and solids	Only for some paramagnetic complexes<br>Radicals need spin traps	Oxidation state<br>Coordination geometry<br>Nuclearity<br>Binding sites
IR/Raman	Fast, facile preparation<br>Label-free cell lysate analysis	Mostly solids or powders<br>Computational analysis needed	Coordination sphere<br>Binding sites<br>Stereochemistry
UV-vis	Simple and versatile<br>Compatible with different solvent matrices	Analysis of mixtures is difficult	Equilibria and kinetics<br>Electronic transitions<br>Geometry
CD	Specific for chiral centres<br>High sensitivity<br>Fast, simple	Limited solvent choice<br>Low resolution<br>Interpretation of bands	Absolute configuration<br>Enantiomeric excess<br>Impact of biomolecular stereochemistry
Fluorescence	Fast<br>High sensitivity<br>Solution, cell, and *in vivo* samples	Often relies on external fluorophore<br>Quenching by environment-wavelength shifts	Oxidation state<br>*In situ* tracking of drug activation<br>Cellular targets and localization<br>Binding affinity and target sites
HPLC	Mixtures<br>Analytical or preparative<br>Nanoscale possible	Quantitation depends on detector<br>Time-consuming<br>Conditions can change speciation	Sample purity<br>Relative lipophilicity
Mass spectrometry	Fast, sensitive<br>Quantitative<br>Isotope pattern identifies elements	Only some analytes<br>Ionization of metal complexes unpredictable<br>Avoid ligand loss on ionisation	Molecular formula<br>Metal oxidation state<br>Potential modification of protein/DNA<br>Binding site(s)
Ion mobility	Fast, sensitive<br>Coupling with MS	Low-resolution (overall size only)	Isomers<br>Conformation
ICP (AES/MS)	Multi-element<br>Sensitive<br>Can be hyphenated (HPLC, single cells)	Digested samples<br>Element only<br>Interferences<br>Sample prep critical	Multi-element concentrations

Table S6. Cisplatin cytotoxicity assays.

Cell line	Medium	Solvent for cisplatin	Treatment time (h)	Assay	IC_{50}	Ref
MCF7 (a)	RPMI-1640	0.5% DMSO	48	SRB	20.1 ± 3.5	[2]
MCF7 (b)	RPMI-1640*, DMEM**	Saline	72	MTT	2.42 ± 0.04	[3]
MCF7 (c)	RPMI-1640	0.5% DMF	48	MTT	13.98 ± 2.02	[4]
MDA-MB-231 (d)	RPMI-1640	0.5% DMSO	48	SRB	32.4 ± 7.4	[2]
MDA-MB-231 (e)	RPMI-1640[a], DMEM[b]	Saline	72	MTT	3.53 ± 0.10	[3]
MDA-MB-231 (f)	DMEM	0.5% DMF	48	MTT	2.33 ± 0.40	[4]

* Stock solution. ** Cell culture.

Table S7. Examples of target classes recommended for identification of off-target effects.

Target	Examples	How determined	Ref
GPCRs	Muscarinic receptors, endothelin A receptors, serotonine receptor	Measurements of second messengers release (cAMP, calcium), binding of ^{35}S-labelled GTPγs	[5]
Ion Channels	hERG, other ion gated ion channels, ligand gated channels	Population patch clamping, flux assays, fluorescence-based assays	[5, 6]
Enzymes	Cyclooxygenase II, monoamine oxidase, phosphodiesterase, acetylcholinesterase, kinases	Incubation with substrates/products with clear readout	[5]
Nuclear receptors	Androgen and glucocorticoid receptors	Gene reporters, FRET	[5]

Table S8. Cell death pathway inhibitors.

Agent	Function	Description
Z-VAD-FMK	Apoptosis inhibitor	Irreversible, cell-permeable, pan-caspase inhibitor
Z-DEVD-FMK	Apoptosis inhibitor	Specific and irreversible caspase-3 inhibitor
Etoposide	Apoptosis activator	Topoisomerase II inhibitor
Nutlin-3a	Apoptosis activator	Murine double minute (MDM2) antagonist. Inhibits MDM2-p53 interactions, stabilizes the p53 protein
Necrostatin-1	Necroptosis inhibitor	Allosteric inhibitor of the death domain receptor-associated adaptor kinase RIP (RIP1)
Necrosulfonamide	Necroptosis inhibitor	Targets the mixed lineage kinase domain-like protein (MLKL). Blocks the formation of necrosomes
Ferrostatin-1	Ferroptosis inhibitor	Potent inhibitor of erastin-induced ferroptosis
Erastin	Ferroptosis activator	Binds and inhibits voltage-dependent anion channels (VDAC2/VDAC3)
3-methyladenine	Autophagy inhibitor	PI3K inhibitor
Chloroquine	Autophagy inhibitor	Toll-like receptors (TLRs) and autophagy inhibitor
Bafilomycin A1	Autophagy inhibitor	Specific inhibitor of vacuolar-type H+ ATPase
Rapamycin	Autophagy activator<br>Immunosuppressant	mTOR inhibitor
Cyclosporine A	Immunosuppressant	Binds to cyclophilin. Inhibits phosphatase activity of calcineurin
Pomalidomide	Immunomodulator	TNF-α release inhibitor

Table S9. Stages of cell cycle arrest for ruthenium anticancer complexes.

Complex	Stage	Information	Ref
RM175	G_1/G_2	In HCT-116 cells: upregulates tumour suppressors p53/p21 (essential for G_1-phase cell cycle arrest). GADD45 and p21 activation by p53 could attribute for G_2/M arrest	7
NAMI-A	G_2/M	Increases expression of CDK inhibitors such as p21, involved in cell cycle arrest	7
NKP-1339	G_2/M	Alters the cellular redox balance by increasing ROS concentration, initiates intrinsic and extrinsic apoptosis	7
$[Ru(dmb)_2(NMIP)](ClO_4)_2$<br>$[Ru(phen)_2(NMIP)](ClO_4)_2$	G_0/G_1<br>S	Decreases membrane potential in mitochondria and augments ROS levels, induces apoptosis	8

Table S10. RNAi studies for cisplatin.

Gene knockdown	Function	Title	Ref
CTR1	Drug uptake	Role of the Copper Transporter, CTR1, in Pt-Induced Ototoxicity	9
ATP7B	Drug uptake	Therapeutic Targeting of ATP7B in Ovarian Carcinoma	10
ATP7A	Drug uptake	ATPase copper transporter A, negatively regulated by miR-148a-3p, contributes to cisplatin resistance in breast cancer cells	11
MRP2	Drug uptake	Role of multidrug resistance protein 2 (MRP2) in chemoresistance and clinical outcome in oesophageal squamous cell carcinoma	12
Metallothionein	Drug inactivation	Inhibition of Cisplatin-Resistance by RNA Interference Targeting Metallothionein Using Reducible Oligo-Peptoplex	13
BOK/BCL2L11<br>TP53/CHEK2/CHEK1/ ATR/SMG1/PRKDC	Apoptosis<br>DNA repair, DNA damage response	A subset of platinum-containing chemotherapeutic agents kills cells by inducing ribosome biogenesis stress rather than by engaging a DNA damage response	14
p53	DNA repair, apoptosis	Glutathione depletion sensitizes cisplatin- and temozolomide-resistant glioma cells *in vitro* and *in vivo*	15
XPF	DNA repair	Cisplatin Resistance in Osteosarcoma: *In vitro* Validation of Candidate DNA Repair-Related Therapeutic Targets and Drugs for Tailored Treatments	16
ERCC1	DNA repair	ERCC1 siRNA Ameliorates Drug Resistance to Cisplatin in Gastric Carcinoma	17
FANCF/FANCL/ FANCD2	DNA repair	RNA interferences targeting the Fanconi anaemia/BRCA pathway upstream genes reverse cisplatin resistance in drug-resistant lung cancer cells	18
HDAC1/2	Cancer initiation and progression	Suberoylanilide hydroxamic acid (SAHA) reverses chemoresistance in head and neck cancer cells by targeting cancer stem cells via the downregulation of nanog	19

Table S11. Characteristic electron-binding energies and X-ray emissions of elements.[a]

Element	Electron-binding energy (eV)	X-ray emission	Energy (eV)	Element	Electron-binding energy (eV)	X-ray emission	Energy (eV)
Be	111.5	K_α	109	Cd	3538	L_α	3134
B	188	K_α	183	In	3730	L_α	3287
C*	284.2	K_α	277	Sn	3929	L_α	3444
N*	409.9	K_α	392	Sb	4132	L_α	3605
O*	543.1	K_α	525	Te	4341	L_α	3769
F	696.7	K_α	677	I*	4557	L_α	3938
Ne	870.2	K_α	849	Xe	4786	L_α	4110
Na*	1070.8	K_α	1041	Cs	5012	L_α	4287
Mg*	1303.0	K_α	1254	Ba	5247	L_α	4466
Al	1559.6	K_α	1487	La	5483	L_α	4651
Si*	1839	K_α	1740	Ce	5723	L_α	4840
P*	2145.5	K_α	2014	Pr	5964	L_α	5034
S*	2472	K_α	2308	Nd	6208	L_α	5230
Cl*	2822.4	K_α	2622	Pm	6459	L_α	5432
Ar	3205.9	K_α	2958	Sm	6716	L_α	5636
K*	3608.4	K_α	3314	Eu	6977	L_α	5846
Ca*	4038.5	K_α	3692	Gd	7243	L_α	6057
Sc	4492	K_α	4091	Tb	7514	L_α	6273
Ti	4964	K_α	4511	Dy	7790	L_α	6595
V*	5465	K_α	4952	Ho	8071	L_α	6720
Cr	5989	K_α	5415	Er	8358	L_α	6949
Mn*	6539	K_α	5899	Tm	8648	L_α	7180
Fe*	7112	K_α	6404	Yb	8944	L_α	7416
Co*	7709	K_α	6930	Lu	9244	L_α	7656
Ni*	8333	K_α	7478	Hf	9561	L_α	7899
Cu*	8979	K_α	8048	Ta	9881	L_α	8146
Zn*	9659	K_α	8639	W*	10,207	L_α	8398
Ga	10,367	K_α	9251	Re	10,535	L_α	8653
Ge	11,103	K_α	9886	Os	10,871	L_α	8912
As	11,867	K_α	10,543	Ir	11,215	L_α	9175
Se*	12,658	K_α	11,224	Pt	11,564	L_α	9442
Br*	13,474	K_α	11,924	Au	11,919	L_α	9713
Kr	14,326	K_α	12,648	Hg	12,284	L_α	9989
Rb	15,200	K_α	13,396	Tl	12,658	L_α	10,268
Sr	16,105	K_α	14,165	Pb	13,035	L_α	10,552
Y	17,038	K_α	14,958	Bi	13,419	L_α	10,839
Zr	17,998	K_α	15,775	Po	13,814	L_α	11,131
Nb	18,986	K_α	16,615	At	14,214	L_α	11,427
Mo*	20,000	K_α	17,480	Rn	14,619	L_α	11,727
Tc	21,044	K_α	18,367	Fr	15,031	L_α	12,031
Ru	22,117	K_α	19,279	Ra	15,444	L_α	12,340
Rh	23,220	K_α	20,216	Ac	15,871	L_α	12,652
Pd	24,350	K_α	21,177	Th	16,300	L_α	12,969
Pd	3173	L_α	2838	Pa	16,733	L_α	13,291
Ag	25,514	K_α	22,163	U	17,166	L_α	13,615
Ag	3351	L_α	2984	-	-	-	-

[a] Data taken from X-Ray Data Booklet https://xdb.lbl.gov/ Accessed 15th October 2020

Electron-binding energies correspond to K_{1s} electrons for monitoring the K_α XRF emissions and L_3 $2p_{3/2}$ electrons for monitoring the L_α emissions. The blue box indicates elements in the 'water-region' (transparent to soft x-rays) typically used in cryo-SXRT. The red box indicates elements which have been mapped *in vitro*, in cells *ex vivo* and *in vivo* and using hard-XRF. *Endogenous elements.

3. Extended Reading

S1. Viral Assays

Viral plaque assays determine the number of plaque forming units (pfu) in a virus sample. A confluent monolayer of host cells is exposed to the virus at varying dilutions and covered with a semi-solid medium, such as agar. This prevents the virus from spreading indiscriminately. A viral plaque is then formed when a cell is infected within the monolayer. This plaque can be observed with an optical microscope or visually (via the use of crystal violet). The plaques are then counted, and the number of pfu per sample unit volume (pfu/mL) is calculated, providing the number of infective particles within the sample, based on the assumption that each plaque represents one infective virus particle.[20] The antiviral activity is then inferred from concentration-dependent reduction of infective particles.

While different types of ELISA assay exist, in the most relevant workflow for direct virus detection, viral particles are first immobilized on a solid surface. Then, this surface is washed with a solution containing an antibody linked to an enzyme. Detection is then accomplished by assessing the conjugated enzyme activity via incubation with a substrate to produce a measureable effect, *e.g.* a colour change. The most crucial element of the detection strategy is a highly specific antibody-antigen interaction.[21]

Quantitative real-time PCR relies on the amplification of a targeted DNA molecule. Selectivity is achieved by the use of specific primers. For RNA viruses, reverse transcription of viral genetic material to complementary DNA is required. 'Two-enzyme, two-tube', 'two-enzyme, one-tube', and 'one-enzyme, one tube' (utilising DNA- and RNA-dependent Tth polymerase) variants of this reaction exist.[22, 23]

S2. Analytical techniques for metallodrug speciation

Several common analytical techniques have metal-specific features, which can be used for characterising metallodrugs and their metabolites and detecting them in biological media. The discussion here is not exhaustive, and certain important techniques (e.g. Mössbauer spectroscopy) will not be discussed. The combined application of a range of them is usually necessary.

NMR. As a semi-quantitative, non-destructive technique probing both covalent and long-range non-covalent interactions, NMR spectroscopy is widely used for metallodrug speciation, often for solutions of isolated metallodrugs or complexes with specific biomolecules. It can provide information about multiple species simultaneously without need for prior separation, allowing analysis of cell lysates or other biological materials.[24] However, it is relatively insensitive for physiologically-relevant drug concentrations, requiring tens of micromolar for even the most sensitive nucleus ^{1}H (^{3}H is more sensitive, but radioactive).

Spin-½ nuclei give the sharpest resonances, the most relevant for metallodrugs being ^{1}H, ^{13}C, ^{15}N, ^{19}F, ^{31}P, ^{195}Pt, ^{187}Os, and ^{103}Rh (See Table S11).[25] However, low gyromagnetic ratios (^{103}Rh) and/or low natural abundance (^{187}Os) can make observation difficult. Quadrupolar nuclei ($I > ½$) usually give broad peaks, reducing sensitivity. Mn, Cu, Zn and Ir, for example, only have quadrupolar isotopes. The large quadrupole moments of Au and Bi (both mononuclidic elements) cause line broadening to the extent that their NMR signals cannot be observed. Moreover, if complexes are paramagnetic then resonances can become extremely broad and greatly shifted, although these effects can sometimes be used to advantage.

NMR studies on metallic NMR-active nuclei can provide valuable insight into the metal oxidation state and the coordination sphere around the metal centre. Metal oxidation state can often be inferred either by line broadening due to paramagnetism, or by chemical shift, as $^{195}Pt(II)$, for instance, appears at much higher field than $^{195}Pt(IV)$. The coordination sphere can be inferred by changes in chemical shift, or by use of 2D NMR experiments, usually involving ^{1}H, ^{15}N, or other organic nuclei.

For Pt drugs, for instance, use of ^{195}Pt NMR and ^{13}C NMR, in tandem with [^{1}H, ^{15}N] HSQC NMR using ^{15}N-labelled complexes (the intensity of ^{15}N resonances being enhanced by polarisation transfer from ^{1}H) can be highly effective for both equilibrium and kinetic studies; the combination of these three techniques recently elucidated the biological ligands capable of activating oxaliplatin.[26] ^{195}Pt shifts and couplings are sensitive to the oxidation state of Pt, the types of ligands, and coordination geometry;[27,26] for instance, a combination of ^{195}Pt and ^{19}F NMR can monitor the hydrolysis products of a Pt(IV) prodrug.[28]

NMR speciation studies on cell lysates or tissue extracts are uncommon outside of the context of ^{1}H NMR metabolomic studies (*vide supra*). Dynamic nuclear polarization, which increases NMR sensitivity by transferring spin polarization from a paramagnetic polarizing agent, was recently reported to improve live-cell and cell lysate NMR signals; although there are not yet reports of live-cell dynamic nuclear polarization studies of metallodrugs, this could be a useful method to observe metallic NMR signals in biological samples.[29]

EPR. Paramagnetic complexes give rise to EPR spectra, but peaks are usually relatively broad and detection limits, at best, are in the millimolar range. Paramagnetic ions such as Mn(II), V(IV), Cu(II) and Gd(III) give signals at ambient temperature, but others such as Co(II), Fe(III) and Ru(III) (see Table S12)[30] require much lower temperatures, and spectra become more complicated.

Advanced EPR methods can probe metal-ligand bonds through hyperfine interactions, including electron-nucleus double resonance (ENDOR), electron spin echo envelope modulation (ESEEM), and 2D hyperfine sublevel correlation (HYSCORE).[30]

ENDOR has been used to deconvolute hyperfine interactions between ^{1}H, ^{14}N, and Ru(III) in NAMI-A, which confirmed coordination of NAMI-A to HSA through His residues.[31]

EPR measurements are possible for biological materials; EPR of fractionated lysates of *Saccharomyces cerevisiae* cells showed that KP1019 was more localised in cell walls compared to NAMI-A, which localises mainly in the cytoplasm and mitochondria.[30]

Vibrational spectroscopy. Vibrational spectroscopy involves relatively easy, label-free sample preparation with short data collection times. However, many complexes are studied in isolation as a powder, pellet, or evaporated solution, which limits the applicability. Computational studies are often needed to assign fully the observed vibrations.

The exclusion rule allows distinction between *cis* and *trans* isomers of metallodrugs. *Trans* isomers generally contain an inversion centre at the metal, so M-L vibrations should not appear in the IR and Raman spectra, while *cis* isomers should show all vibrations in both spectra; this has been used to distinguish between stereoisomers of square-planar anticancer Au(III) complexes.[32]

Intensity changes of IR amide I-III bands and bands assigned to specific amino acids can identify metallodrug-protein binding sites. Gas-phase IR multiphoton dissociation spectroscopy elucidated different binding modes to methionine residues for cisplatin and transplatin. The asymmetric $-NH_2$ stretch at 3440 cm^{-1} indicated that cisplatin prefers S-ligated complexes, while transplatin favours N-ligation.[33]

UV-vis. UV-vis measurements are generally fast and technically unchallenging. They are compatible with a wide variety of solvents and matrices, thereby simplifying studies on parameters like solvent and pH effects on metallodrug reactions. The most prominent drawbacks of UV-vis are the inability to identify definitively more than two analytes in the same solution, usually due to broad, overlapping bands for structurally similar molecules, and the difficulty in obtaining measurements for dilute samples, both of which restrict its direct use on biological samples.

Metal-specific absorptions include metal-centred d-d transitions and ligand-to-metal and metal-to-ligand charge transfer (LMCT, MLCT) bands, all of which provide markers to monitor drug reactivity kinetics and biomolecular target binding.

The wavelength and molar absorptivity of d-d transitions, which reflect the energy and 'allowedness' of the transition, respectively, can be combined to infer geometric information about metal complexes. Charge-transfer bands are particularly important in the context of photoactivated metallodrugs. For example, photoactivated Ru(II) bipyridine carbonyl complexes release CO upon irradiation of the MLCT/LMCT bands near 350 nm.[34]

Circular Dichroism. CD spectroscopy measures the differential absorption of circularly polarized light. Since compounds with chiral centres interact differently with left- and right-handed polarized light, CD is a useful technique to study the structures of chiral complexes. The metal itself may be a chiral centre, or chirality may come from ligands. Induced chirality in metal complexes can arise through interactions with chiral biomolecules, a powerful way of using metal absorption bands to study such binding.

Although CD cannot provide detailed structural information, it is fast and requires very little sample. CD measurements are largely confined to *in vitro* solution samples, as unfiltered

samples of cell lysates or biological fluids often contain too many chiral biomolecules to study any one specific molecule or interaction.

The CD spectrum provides a fingerprint for a particular enantiomer.[35] Such information can be extremely important for a drug since enantiomers may have differing biological activity if they involve reactions with chiral biomolecules. For example, the *R,R* isomer of oxaliplatin is more active than its *S,S* counterpart,[36] and anticancer Ru and Ir complexes have enantioselective binding affinities for target proteins such as glycogen synthase kinase 3 (GSK-3), carbonic anhydrase II, histone deacetylases, and trypsin, affecting their activity as inhibitors.[37, 38]

Mass spectrometry. The necessary ions are usually produced using electrospray ionisation, which allows retention of metal-bound ligands in the gas phase.[39] MS requires little sample and allows fast measurement, making it valuable for high-throughput experiments. A potential limitation is that highly acidic analytes may not readily form positive ions. High mass accuracy can provide unequivocal assignments of elemental composition.[40] Typically, this requires sub-ppm accuracy, achievable with Fourier transform ion cyclotron resonance (FTICR) and Orbitrap instruments.[41] These provide resolving powers up to hundreds of thousands or even millions, allowing separation of isotope peaks. Many transition metals , *e.g.* Pt, Ir, and Os, have characteristic isotope ratios generating recognisable patterns.[42] Such patterns enable rapid and efficient screening of large LC-MS datasets for peptides containing such metallodrug fragments.[42] By counting charge carriers with fixed oxidation states such as H^+/Na^+ while knowing the net charge state of an ion, the oxidation state of a metal centre can be easily calculated.[43]

Finally, at extremely high resolving power, the so-called 'fine isotope distribution',[44] allows resolution of isotopologues with the same nucleon count, but with neutrons added to different nuclei, for instance $^{16}O^{13}C^{32}S$ and $^{16}O^{12}C^{33}S$ (See Figure S1). Due to different nuclear binding energies, these ions have a slightly different mass (*ca.* 4 mDa mass difference for OCS), and this provides strong evidence for a postulated molecular formula.[45]

All the benefits outlined here also apply in tandem MS experiments, in which a precursor ion is isolated and fragmented in the gas phase, *e.g.* determination of oxaliplatin-treated ubiquitin

platination sites by FTICR-based tandem MS as the N-terminal Met, whereas transplatin binds to the P(19)SDTIE(24) region.[46]

Ion mobility MS. Ions in IM spectrometry are separated by their electrophoretic mobility, dependent on their collision cross-section (CCS) as they move through a collision gas (*e.g.* N_2).[47] This allows separation of isomers of the same ion, for example anticancer organoruthenium(II) complexes containing *ortho*-, *meta*-, or *para*-terphenyl arene ligands (*e.g.* **Ru17**).[48]

From reactions of $[(\eta^6\text{-bip})Ru(en)Cl]^+$ with d(CACGTG), IMS can separate several conformations of mono- and di-ruthenated species on the basis of the change in size and shape.[49] Conformational changes in platinated proteins have also be detected by IMS, *e.g.* for ubiquitin adducts with cisplatin.[46,50]

S3. Methods for cell preservation

Chemical fixation. One of the most common methods of cell and tissue preservation for biological research is chemical fixation: using chemicals to prevent enzymatic autolysis. Chemical fixatives can be broadly categorized as crosslinking, precipitating, or oxidizing fixatives. Crosslinking fixatives include formaldehyde and glutaraldehyde, which bind covalently to proteins – preserving both chemical and structural integrity.[51] Precipitating fixatives work by reducing protein solubility (cell dehydration), causing precipitation of proteins.[52] In particular, ethanol and methanol are precipitating fixatives which are commonly employed in flow cytometry and confocal microscopy. Finally, oxidizing chemical fixatives (*e.g.* osmium tetroxide) react with biomolecules and proteins to stabilize cellular or tissue structure.[53] The efficiency of chemical fixation depends on a variety of factors: pH, time, temperature, osmolarity, penetration rate, and volume ratio.[54] The advantages of preserving biological samples with chemical fixatives include rapid preservation, good preservation of cellular morphology, suitable tissue penetration, and low expense. However, chemical fixation can be disadvantageous as it can alter endogenous factors (*i.e.* cell volume, ATP content),[55] as they typically bind to proteins (which may alter the observed drug distribution), have detrimental effects on DNA and can be toxic (Table S14).[55, 56]

Cryopreservation. Cells and tissues can be cryopreserved by plunge-freezing samples in liquid ethane (-170 °C), before storage at liquid nitrogen temperatures. Cryopreservation fixes biological specimens as close to their native state as possible (besides live analysis), without the need for invasive chemical fixatives. In general, cryopreservation provides the best preservation of biological samples; however, it is more challenging and requires specialist cryogenic equipment such as electron microscopes which operate under liquid nitrogen conditions. For example, the B24 cryo-soft X-ray Tomography beamline of the Diamond Light Source (Oxfordshire, UK) allows the analysis of vitrified and hydrated biological samples down to 30-40 nm spatial resolution.[57] However, a major disadvantage is that frozen samples are more susceptible to beam damage in electron microscopy.[58]

Freeze-drying. Freeze-drying can be used to preserve cells in a dehydrated state, for analysis and storage at room temperature. Biological samples can be frozen at -80 °C, after which the ice is removed under vacuum. Advantages of freeze-drying include good preservation of nucleic acids, long-term sample storage, avoiding the use of chemical fixatives, ease to handling, versatility, and low cost. However, both freezing and drying can damage biomolecules so cryoprotectants are often required) and the process can be time-consuming. Often, cryopreserved cells (frozen in liquid nitrogen, -196 °C) are freeze-dried using a gradual temperature gradient, for analysis at room temperature.[59]

Hypothermic preservation. Hypothermic preservation differs to that of cryopreservation and freezing in that it preserves cells at temperatures between 4 to 10 °C.[60] In hypothermic preservation, cells are incubated in a preservation solution, which reduces cellular stress from extreme temperature changes (cooling / warming). Cells can remain in this preservation solution at temperatures up to 10 °C for short periods of time, which can be removed and replaced with fresh culture media (37 °C) prior to use. The hypothermic preservation of biological specimens reduces the level of cellular stress; it is simple and cheap compared to cryopreservation. However, the main disadvantage of hypothermic preservation is the short-term storage (hours to weeks).

Sectioning. Sectioning of tissues is commonly used for immunohistochemistry – a microscopy technique involving immunostaining used to image cellular components. The first stage of sectioning involves fixation of biological samples – either chemically (*e.g.* aldehyde fixation) or cryogenically (immersion in isopentane or liquid nitrogen). The fixed samples can then be embedded in either paraffin wax (chemically-fixed samples) or using an Optimal Cutting Temperature (OCT) compound (cryopreserved samples),[61] ready for sectioning into thin slices using a microtome or cryostat, respectively. Chemically-fixed, paraffin-embedded tissue sections have advantages over frozen, OCT-embedded samples: good preservation of biomolecules, long-term storage and versatility. In contrast, frozen tissue sections provide information closer to the native, endogenous state of the tissue and preserves enzymatic functions; however, these samples are significantly softer (making them difficult to handle) and the presence of ice on the sample can alter tissue structure.

Table S12. NMR parameters for commonly used nuclei in metallodrug speciation. For atoms with more than one NMR-active nucleus, the preferred isotope is bold.[62, 63]

Nucleus	Natural Abundance (%)	Spin Quantum Number (I)	Quadrupole Moment (10^{28} * Q/m^2)	Absolute Sensitivity (^{1}H = 1.00)	Reports of Use in Biological Systems
**Non-metals**					
^{1}H	99.98	1/2	0	1.00	Yes
^{13}C	1.108	1/2	0	1.76 x 10^{-4}	Yes
^{15}N	0.365	1/2	0	3.85 x 10^{-6}	Yes
^{17}O	0.037	5/2	-2.6 x 10^{-2}	1.08 x 10^{-5}	Yes
^{19}F	100	1/2	0	0.83	Yes
^{31}P	100	1/2	0	6.63 x 10^{-2}	Yes
^{33}S	0.76	3/2	-5.5 x 10^{-2}	1.72 x 10^{-5}	Yes
**Metals with relatively easy collection & analysis**					
^{107}Ag	51.82	1/2	0	3.43 x 10^{-5}	Some
**^{109}Ag**	48.18	1/2	0	4.86 x 10^{-5}	Yes
^{195}Pt	33.7	1/2	0	3.36 x 10^{-3}	Yes
**Metals with collection & analysis complications**					
^{6}Li	7.42	1	-8 x 10^{-4}	6.31 x 10^{-4}	
^{7}Li	92.58	3/2	-4 x 10^{-2}	0.27	Yes
^{25}Mg	10.13	5/2	0.22	2.71 x 10^{-4}	Yes
^{27}Al	100	5/2	0.15	0.21	Yes
^{50}V	0.24	6	6 x 10^{-2}	1.33 x 10^{-4}	
**^{51}V**	99.76	7/2	-5 x 10^{-2}	0.38	Yes
^{57}Fe	2.19	1/2	0	7.38 x 10^{-7}	Some
^{59}Co	100	8/2	0.38	0.28	Yes
**^{63}Cu**	69.09	3/2	-0.211	6.433 x 10^{-2}	No
^{65}Cu	30.91	3/2	-0.195	3.52 x 10^{-2}	No
^{67}Zn	4.11	5/2	0.16	1.17 x 10^{-4}	Yes
^{69}Ga	60.4	3/2	0.19	4.17 x 10^{-2}	
**^{71}Ga**	39.6	3/2	0.12	5.62 x 10^{-2}	Yes
^{75}As	100	3/2	0.29	2.51 x 10^{-2}	No
^{103}Rh	100	1/2	0	3.11 x 10^{-5}	No
**^{119}Sn**	8.58	1/2	0	4.44 x 10^{-3}	Yes
**^{121}Sb**	57.25	5/2	-0.28	9.16 x 10^{-2}	No
^{123}Sb	42.75	7/2	-0.36	1.95 x 10^{-2}	No
**Metals with difficult collection & analysis**					
^{43}Ca	0.145	7/2	0.2	9.28 x 10^{-6}	Yes
^{53}Cr	9.55	3/2	3 x 10^{-2}	8.62 x 10^{-3}	No
^{61}Ni	1.19	3/2	0.16	4.25 x 10^{-5}	No
^{55}Mn	100	5/2	0.4	0.18	No
^{95}Mo	15.72	5/2	0.12	5.07 x 10^{-4}	No
^{97}Mo	9.46	5/2	1.1	3.24 x 10^{-4}	No
**^{99}Ru**	12.72	3/2	7.6 x 10^{-2}	2.48 x 10^{-5}	No
^{101}Ru	17.07	5/2	0.44	2.4 x 10^{-4}	No
^{183}W	14.4	1/2	0	1.03 x 10^{-5}	No

**^{187}Os**	**1.64**	**1/2**	**0**	**2.00×10^{-7}**	**No**
^{189}Os	**16.1**	**5/2**	**0.8**	**3.76×10^{-4}**	**No**
^{191}Ir	37.3	3/2	1.1	9.43×10^{-6}	No
^{193}Ir	62.7	3/2	1.0	2.05×10^{-5}	No
^{197}Au	100	3/2	0.59	2.51×10^{-5}	No
^{209}Bi	100	9/2	-0.38	0.13	No

Table S13. EPR parameters of some common paramagnetic metallodrug ions.[30, 64, 65]

Metal ion	Spin system (S)	Hyperfine coupling (natural abundance)	Electronic relaxation rate (τ_s^{-1} s^{-1})
Cr(III)	3/2	^{53}Cr, I = 3/2 (9.5%)	2×10^9
Mn(II)	5/2	^{55}Mn, I = 5/2 (100%)	10^8 - 10^9
Fe(III)	5/2 (high-spin)	^{57}Fe, I = ½ (2%)	10^{10} - 10^{11}
Fe(III)	1/2 (low-spin)	^{57}Fe, I = ½ (2%)	10^{11} - 10^{12}
Co(II)	3/2 (high-spin)	^{59}Co, I = 7/2 (100%)	10^{11} - 10^{12}
Co(II)	1/2 (low-spin)	^{59}Co, I = 7/2 (100%)	10^9 - 10^{10}
Cu(II)	1/2	$^{63/65}Cu$, I = 3/2 (69/31%)	3×10^8 - 10^9
Ru(III)	½	$^{99/101}Ru$, I = 5/2 (13/17%)	10^{11} - 10^{12}

Table S14. Summary of cell and tissue preservation methods and their advantages and disadvantages.

Preservation method	Fixative	Advantages	Disadvantages
Chemical fixation	Crosslinking (aldehydes)	Good cell preservation<br>Good tissue preservation<br>Simplicity<br>Rapid preservation<br>Fast tissue penetration<br>Cheap	Affects endogenous factors<br>Protein-binding<br>Detrimental to DNA<br>Cause cell shrinkage<br>Toxic
Chemical fixation	Precipitating (alcohols)	Good cell preservation<br>Good tissue preservation<br>Rapid dehydration<br>Suitable for studying DNA and RNA	Loss of cellular content (particularly lipids)
Chemical fixation	Oxidizing agents (osmium tetroxide)	Good cell preservation<br>Good tissue preservation<br>Electron microscopy<br>Lipid interactions	Limited penetration<br>Cause tissue swelling<br>Highly toxic
Cryopreservation	Vitrification	Good cell preservation<br>Good tissue preservation<br>Near physiological state	Challenging set up<br>Susceptible to beam damage in EM
Freeze-drying	Freeze-drying	Good preservation of nucleic acids<br>Simplicity<br>Long-term storage<br>Versatile<br>Cost effective	Damage / stress to biomolecules<br>Sometimes requires cryoprotectants<br>Time-consuming
Hypothermic preservation	Hypothermic preservation	Avoids cryoprotectants<br>Reduced cell stress<br>Simplicity<br>Cheap	Short-term storage
Sectioning	Wax-embedding	Good tissue preservation<br>Good preservation of proteins and DNA<br>Long-term storage<br>Versatility	Damage epitopes of target antigens
Sectioning	Cryopreserved	Near native state<br>Good tissue preservation<br>Good preservation of enzymatic activity	Ice damage to tissue structure<br>Soft embedding is difficult to handle

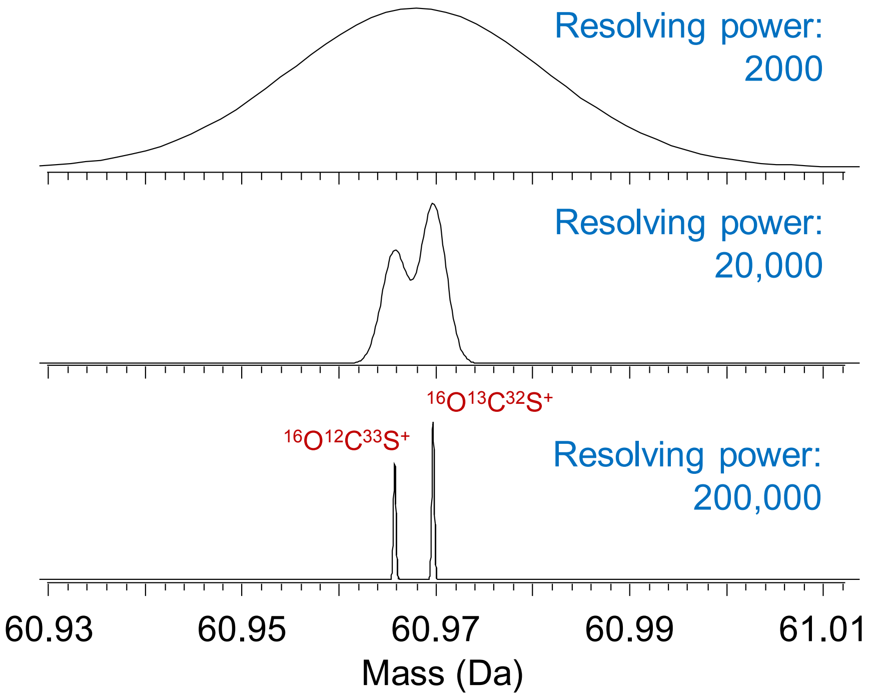

Figure S1. Simulated fine isotope pattern for the (A+1) peak of the OCS^+ ion, with a nominal mass of 61 Da. The panels show the result of acquiring the spectrum at three different resolving powers.

4. References

1. M. Balouiri, M. Sadiki and S. K. Ibnsouda, *J. Pharm. Anal.*, 2016, **6**, 71-79.
2. G. Golbaghi, I. Pitard, M. Lucas, M. M. Haghdoost, Y. L. de los Santos, N. Doucet, S. A. Patten, J. T. Sanderson and A. Castonguay, *Eur. J. Med. Chem.*, 2020, **188** (DOI: 10.1016/j.ejmech).
3. L. Tabrizi, L. O. Olasunkanmi and O. A. Fadare, *Dalton Trans.*, 2019, **48**, 728-740.
4. K. M. Oliveira, E. J. Peterson, M. C. Carroccia, M. R. Cominetti, V. M. Deflon, N. P. Farrell, A. A. Batista and R. S. Correa, *Dalton Trans.*, 2020 (DOI: 10.1039/D0DT01091J).
5. J. Bowes, A. J. Brown, J. Hamon, W. Jarolimek, A. Sridhar, G. Waldron and S. Whitebread, *Nat. Rev. Drug Discov.*, 2012, **11**, 909-922.
6. B. T. Priest, I. M. Bell and M. L. Garcia, *Channels*, 2008, **2**, 87-93.
7. J. P. Coverdale, T. Laroiya-McCarron and I. Romero-Canelón, *Inorganics*, 2019, **7**, 31.
8. S.-H. Liu, J.-W. Zhu, H.-H. Xu, Y. Wang, Y.-M. Liu, J.-B. Liang, G.-Q. Zhang, D.-H. Cao, Y.-Y. Lin and Y. Wu, *Spectrochim. Acta A.*, 2016, **161**, 77-82.
9. S. S. More, O. Akil, A. G. Ianculescu, E. G. Geier, L. R. Lustig and K. M. Giacomini, *J. Neurosci.*, 2010, **30**, 9500-9509.
10. L. S. Mangala, V. Zuzel, R. Schmandt, E. S. Leshane, J. B. Halder, G. N. Armaiz-Pena, W. A. Spannuth, T. Tanaka, M. M. Shahzad, Y. G. Lin, A. M. Nick, C. G. Danes, J. W. Lee, N. B. Jennings, P. E. Vivas-Mejia, J. K. Wolf, R. L. Coleman, Z. H. Siddik, G. Lopez-Berestein, S. Lutsenko and A. K. Sood, *Clin. Cancer Res.*, 2009, **15**, 3770-3780.
11. Z. Yu, W. Cao, Y. Ren, Q. Zhang and J. Liu, *Clin. Transl. Med.*, 2020, **10**, 57-73.
12. M. Yamasaki, T. Makino, T. Masuzawa, Y. Kurokawa, H. Miyata, S. Takiguchi, K. Nakajima, Y. Fujiwara, N. Matsuura, M. Mori and Y. Doki, *Br. J. Cancer*, 2011, **104**, 707-713.
13. J. H. Lee, J. W. Chae, J. K. Kim, H. J. Kim, J. Y. Chung and Y. H. Kim, *J. Control. Release* , 2015, **215**.
14. P. M. Bruno, Y. Liu, G. Y. Park, J. Murai, C. E. Koch, T. J. Eisen, J. R. Pritchard, Y. Pommier, S. J. Lippard and M. T. Hemann, *Nat. Med*, 2017, **23**, 461-471.
15. C. R. R. Rocha, C. C. M. Garcia, D. B. Vieira, A. Quinet, L. C. de Andrade-Lima, V. Munford, J. E. Belizário and C. F. M. Menck, *Cell Death Dis.*, 2014, **5**, e1505.
16. M. Fanelli, E. Tavanti, M. P. Patrizio, S. Vella, A. Fernandez-Ramos, F. Magagnoli, S. Luppi, C. M. Hattinger and M. Serra, *Front. Oncol.*, 2020, **10**, 331.
17. W. Li, Z. Jie, Z. Li, Y. Liu, Q. Gan, Y. Mao and X. Wang, *Mol. Med. Rep.*, 2014, **9**, 2423-2428.
18. C.-H. Dai, J. Li, P. Chen, H.-G. Jiang, M. Wu and Y.-C. Chen, *J. Biomed. Sci.*, 2015, **22**, 1-13.
19. B. Kumar, A. Yadav, J. C. Lang, T. N. Teknos and P. Kumar, *Genes Cancer*, 2015, **6**, 169-181.
20. A. Baer and K. Kehn-Hall, *J. Vis. Exp.*, 2014, **93**, e52065.
21. E. Engvall and P. Perlmann, *J. Immunol*, 1972, **109**, 129-135.
22. W. M. Freeman, S. J. Walker and K. E. Vrana, *Biotechniques*, 1999, **26**, 112-122, 124-115.
23. S. A. Bustin, *J. Mol. Endocrinol.*, 2000, **25**, 169-193.
24. F. De Castro, M. Benedetti, L. Del Coco and F. P. Fanizzi, *Molecules*, 2019, **24**, 14.
25. T. Zou and P. J. Sadler, *Drug Discov. Today Technol.*, 2015, **16**, 7-15.
26. D. Höfer, M. Galanski and B. K. Keppler, *Eur. J. Inorg. Chem.*, 2017, **2017**, 2347-2354.
27. S. J. Berners-Price, L. Ronconi and P. J. Sadler, *Prog. Nucl. Magn. Reson. Spectrosc.*, 2006, **49**, 65-98.
28. E. Wexselblatt, E. Yavin and D. Gibson, *Angew. Chem. Int. Ed.*, 2013, **52**, 6059-6062.

29. P. T. Judge, E. L. Sesti, L. E. Price, B. J. Albert, N. Alaniva, E. P. Saliba, T. Halbritter, S. T. Sigurdsson, G. B. Kyei and A. B. Barnes, *J. Phys. Chem. B*, 2020, **124**, 2323-2330.
30. K. E. Prosser and C. J. Walsby, *Eur. J. Inorg. Chem.*, 2017, 1573-1585.
31. M. I. Webb and C. J. Walsby, *Dalton Trans.*, 2015, **44**, 17482-17493.
32. R. Krikavova, J. Hosek, P. Suchy, J. Vanco and Z. Travnicek, *J. Inorg. Biochem.*, 2014, **134**, 92-99.
33. R. Paciotti, D. Corinti, A. De Petris, A. Ciavardini, S. Piccirillo, C. Coletti, N. Re, P. Maitre, B. Bellina, P. Barran, B. Chiavarino, M. Elisa Crestoni and S. Fornarini, *Phys. Chem. Chem. Phys.*, 2017, **19**, 26697-26707.
34. M. Kubeil, R. R. Vernooij, C. Kubeil, B. R. Wood, B. Graham, H. Stephan and L. Spiccia, *Inorg. Chem.*, 2017, **56**, 5941-5952.
35. Y. Fu, R. Soni, M. J. Romero, A. M. Pizarro, L. Salassa, G. J. Clarkson, J. M. Hearn, A. Habtemariam, M. Wills and P. J. Sadler, *Chem.-Eur. J.*, 2013, **19**, 15199-15209.
36. Y. Wang, H. Y. Huang, Q. L. Zhang and P. Y. Zhang, *Dalton Trans.*, 2018, **47**, 4017-4026.
37. G. E. Atilla-Gokcumen, L. Di Costanzo and E. Meggers, *J. Biol. Inorg. Chem.*, 2011, **16**, 45-50.
38. P. Gobel, F. Ritterbusch, M. Helms, M. Bischof, K. Harms, M. Jung and E. Meggers, *Eur. J. Inorg. Chem.*, 2015, 1654-1659.
39. M. Wenzel and A. Casini, *Coord. Chem. Rev.*, 2017, **352**, 432-460.
40. R. A. Zubarev, P. Hakansson and B. Sundqvist, *Anal. Chem.*, 1996, **68**, 4060-4063.
41. J. Claesen, F. Lermyte, F. Sobott, T. Burzykowski and D. Valkenborg, *Anal. Chem.*, 2015, **87**, 10747-10754.
42. C. A. Wootton, Y. P. Y. Lam, M. Willetts, M. A. van Agthoven, M. P. Barrow, P. J. Sadler and O. C. PB, *Analyst*, 2017, **142**, 2029-2037.
43. F. Lermyte, J. Everett, Y. P. Y. Lam, C. A. Wootton, J. Brooks, M. P. Barrow, N. D. Telling, P. J. Sadler, P. B. O'Connor and J. F. Collingwood, *J. Am. Soc. Mass Spectrom.*, 2019, **30**, 2123-2134.
44. P. Dittwald, D. Valkenborg, J. Claesen, A. L. Rockwood and A. Gambin, *J. Am. Soc. Mass Spectrom.*, 2015, **26**, 1732-1745.
45. S. Banerjee, A. Omlor, J. A. Wolny, Y. Han, F. Lermyte, A. E. Godfrey, P. B. O'Connor, V. Schunemann, M. Danaie and P. J. Sadler, *Dalton. Trans.*, 2019, **48**, 9564-9569.
46. C. G. Hartinger, Y. O. Tsybin, J. Fuchser and P. J. Dyson, *Inorg. Chem.*, 2008, **47**, 17-19.
47. V. Gabelica, A. A. Shvartsburg, C. Afonso, P. Barran, J. L. P. Benesch, C. Bleiholder, M. T. Bowers, A. Bilbao, M. F. Bush, J. L. Campbell, I. D. G. Campuzano, T. Causon, B. H. Clowers, C. S. Creaser, E. De Pauw, J. Far, F. Fernandez-Lima, J. C. Fjeldsted, K. Giles, M. Groessl, C. J. Hogan, Jr., S. Hann, H. I. Kim, R. T. Kurulugama, J. C. May, J. A. McLean, K. Pagel, K. Richardson, M. E. Ridgeway, F. Rosu, F. Sobott, K. Thalassinos, S. J. Valentine and T. Wyttenbach, *Mass. Spectrom. Rev.*, 2019, **38**, 291-320.
48. J. P. Williams, T. Bugarcic, A. Habtemariam, K. Giles, I. Campuzano, P. M. Rodger and P. J. Sadler, *J. Am. Soc. Mass Spectrom.*, 2009, **20**, 1119-1122.
49. J. P. Williams, J. A. Lough, I. Campuzano, K. Richardson and P. J. Sadler, *Rapid Commun. Mass Spectrom.*, 2009, **23**, 3563-3569.
50. J. P. Williams, H. I. Phillips, I. Campuzano and P. J. Sadler, *J. Am. Soc. Mass Spectrom.*, 2010, **21**, 1097-1106.
51. R. Thavarajah, V. K. Mudimbaimannar, J. Elizabeth, U. K. Rao and K. Ranganathan, *J. Oral Maxillofac Pathol.*, 2012, **16**, 400-405.
52. M. C. Jamur and C. Oliver, *Methods Mol. Biol.*, 2010, **588**, 55-61.
53. A. J. Nielson and W. P. Griffith, *J. Histochem. Cytochem.*, 1979, **27**, 997-999.
54. C.-H. Park, H.-W. Kim, I. J. Rhyu and C.-S. Uhm, *Appl. Microsc*, 2016, **46**, 188-192.
55. A. Penttila, H. Kalimo and B. F. Trump, *J. Cell Biol.*, 1974, **63**, 197-214.
56. M. Srinivasan, D. Sedmak and S. Jewell, *Am. J. Pathol.*, 2002, **161**, 1961-1971.

57. R. Carzaniga, M.-C. Domart, E. Duke and L. M. Collinson, in *Methods in Cell Biology*, eds. T. Müller-Reichert and P. Verkade, Academic Press, 2014, vol. 124, pp. 151-178.
58. M. A. Aronova, A. A. Sousa, G. Zhang and R. D. Leapman, *J Microsc.*, 2010, **239**, 223-232.
59. Q. Jin, T. Paunesku, B. Lai, S.-C. Gleber, S. I. Chen, L. Finney, D. Vine, S. Vogt, G. Woloschak and C. Jacobsen, *J. Microsc.*, 2017, **265**, 81-93.
60. C. Correia, A. Koshkin, M. Carido, N. Espinha, T. Šarić, P. A. Lima, M. Serra and P. M. Alves, *Stem Cells Transl. Med*, 2016, **5**, 658-669.
61. R. Zeller, *Curr. Protoc. Mol. Bio.*, 1989, **7**, 14.1.1-8
62. L. Ronconi and P. J. Sadler, *Coord. Chem. Rev.*, 2008, **252**, 2239-2277.
63. A. Levina, D. C. Crans and P. A. Lay, *Coord. Chem. Rev.*, 2017, **352**, 473-498.
64. R. S. Drago and R. S. Drago, *Physical methods for chemists*, Saunders College Pub., Ft. Worth, 1992.
65. Z. Zhao, C. Sun, J. Bao, L. Yang, R. Wei, J. Cheng, H. Lin and J. Gao, *J. Mater. Chem. B*, 2018, **6**, 401-413.

5. Additional examples of recent relevant literature

1. The Elements of Life and Medicines, P. Chellan and P. J. Sadler, *Phil. Trans. R. Soc. A.* 2015, **373**, 20140182.
2. Exploration of the medical periodic table: towards new targets, N. P. Barry and P. J. Sadler, *Chem. Commun*, 2013, **49**, 5106-5131.
3. Designing organometallic compounds for catalysis and therapy, L. Noffke, A. Habtemariam, A. M. Pizarro and P. J. Sadler,*Chem. Commun*, 2012, **48**, 5219-5246.
4. Advances in Metallodrugs: Preparation and Applications in Medicinal Chemistry, ed. S. Ul-Islam, A. A. Hashmi and S. A. Khan, Wiley, 2020.
5. Metallomics: The Science of Biometals and Biometalloids. W. Maret, in Metallomics, Vol 1055, *Adv. Exp. Med. Biol*., Springer, 2018, pp. 1-20.
6. Metals in Medicine, ed. K. J. Franz and N. Metzler-Nolte, *Chem. Rev.* (Special Issue) 2019, 119, 2, pages 727-1624
7. Metallodrugs: Activation, Targeting, and Delivery, ed. N. Metzler-Nolte and Z. Guo, *Dalton Trans.* (Special Issue), 2016, **45**, 12965-13069.
8. Medicinal Chemistry, ed. R. Van Eldik and P. J. Sadler, *Adv. Inorg. Chem.*, Vol. 75, Academic Press, 2020.
9. Essential Metals in Medicine, ed. A. Sigel, H. Sigel, R. K. O. Siegel, Vol. 19 *Met. Ions Life Sci.*, Walter de Gruyter GmbH, Berlin, Boston, 2019
10. Stimuli-Responsive Therapeutic Metallodrugs, X. Wang, X. Wang, S. Jin, N. Muhammad and Z. Guo, *Chem. Rev.*, 2019, **119**, 1138-1192.
11. Photoresponsive ruthenium-containing polymers: potential polymeric metallodrugs for anticancer phototherapy, W. Sun, X. Zeng and S. Wu, *Dalton Trans.*, 2018, **47**, 283-286.
12. Metallo-Drugs: Development and Action of Anticancer Agents, ed. A. Sigel, H. Sigel, R. K. O. Siegel, Vol. 18 *Met. Ions Life Sci.*, Walter de Gruyter GmbH, Berlin, Boston, 2018
13. Preparation and Antitumoral Activity of Au-Based Inorganic-Organometallic Nanocomposites, M. Dalmases, A. Pinto, P. Lippmann, I. Ott, L. Rodríguez and A. Figuerola, *Front. Chem.*, 2019, **7**, 60.
14. NAMI-A and KP1019/1339, Two Iconic Ruthenium Anticancer Drug Candidates Face-to-Face: A Case Story in Medicinal Inorganic Chemistry, E. Alessio and L. Messori, *Molecules*, 2019, **24**, 1995.
15. Metal Anticancer Complexes - Activity, Mechanism of Action, Future Perspectives. Eds. E. Alessio and Z. Guo, *Eur. J. Inorg. Chem.*, 2017, **2017**, 1539-1840.
16. Throwing Light on Recent Advances on Metallodrugs: From Deemed Poisons to a Striking Hope for the Future, ed. C. Nardon and D. Fregona, *Curr. Med. Chem*. (Special Issue), 2018, **25**, 434-555.
17. Exposing "Bright" Metals: Promising Advances in Photoactivated Anticancer Transition Metal Complexes, Bjelosevic, L. K. Spare, K. M. Deo, D. L. Ang and J. R. Aldrich-Wright, *Curr. Med. Chem.*, 2018, **25**, 478-492.
18. Metallodrugs in Targeted Cancer Therapeutics: Aiming at Chemoresistance- related Patterns and Immunosuppressive Tumor Networks, S. Petanidis, E. Kioseoglou and A. Salifoglou, *Curr. Med. Chem.*, 2019, **26**, 607-623.
19. Polyoxometalates as Potential Next-Generation Metallodrugs in the Combat Against Cancer, Bijelic, M. Aureliano and A. Rompel, *Angew. Chem. Int. Ed.*, 2019, **58**, 2980-2999.
20. Catalytic Metallodrugs: Substrate-Selective Metal Catalysts as Therapeutics. Chemistry, Z. Yu and J. A. Cowan, *Chem. Eur. J.*, 2017, **23**, 14113-14127.
21. Ferrocifen type anti cancer drugs, G. Jaouen, A. Vessières and S. Top, *Chem. Soc. Rev.*, 2015, **44**, 8802-8817.

22. Visualization of metallodrugs in single cells by secondary ion mass spectrometry imaging, K. Wu, F. Jia, W. Zheng, Q. Luo, Y. Zhao and F. Wang, *J. Biol. Inorg. Chem.* 2017, **22**, 653-661.
23. Ferrocifens labelled with an infrared rhenium tricarbonyl tag: synthesis, antiproliferative activity, quantification and nano IR mapping in cancer cells, Y. Wang, F. Heinemann, S. Top, A. Dazzi, C. Policar, L. Henry, F. Lambert, G. Jaouen, M. Salmain and A. Vessières, *Dalton Trans.*, 2018, **47**, 9824-9833.
24. Systems Chronotherapeutics, Ballesta, P. F. Innominato, R. Dallmann, D. A. Rand and F. A. Lévi, *Pharmacol. Rev.*, 2017, **69**, 161-199.
25. Cell Cycle-Dependent Uptake and Cytotoxicity of Arsenic-Based Drugs in Single Leukemia Cells, Y. Zhou, H. Wang, E. Tse, H. Li and H. Sun, *Anal. Chem.*, 2018, **90**, 10465-10471.
26. Comparative studies of oxaliplatin-based platinum(iv) complexes in different in vitro and in vivo tumor models, S. Göschl, E. Schreiber-Brynzak, V. Pichler, K. Cseh, P. Heffeter, U. Jungwirth, M. A. Jakupec, W. Berger and B. K. Keppler, *Metallomics*, 2017, **9**, 309-322.
27. Metabolomics in Metal-Based Drug Research, F. De Castro, M. Benedetti, L. Del Coco and F. P. Fanizzi, *Molecules*, 2019, **24**, 2240.
28. Metalloproteomics in conjunction with other omics for uncovering the mechanism of action of metallodrugs: Mechanism-driven new therapy, H. Wang, Y. Zhou, X. Xu, H. Li and H. Sun, *Curr. Opin. Chem. Biol.*, 2020, **55**, 171-179.
29. Frontiers in radionuclide imaging and therapy, a chemical journey from naturally radioactive elements to targeted theranostic agents. Ed. A. Casini, C. Orvig and J. D. Correia, *Dalton Trans.* (Special Issue), 2017, **46**, 14433-14536.
30. A nuclear chocolate box: the periodic table of nuclear medicine, P. J. Blower, *Dalton Trans.* 2015, **44**, 4819-4844.
31. Metalloproteomics for Unveiling the Mechanism of Action of Metallodrugs Y. Wang, H. Li and H. Sun, *Inorg. Chem.*, 2019, **58**, 13673-13685.

6. Metallodrug structures

Structures by metal atomic number	Name	Section
Aluminium		
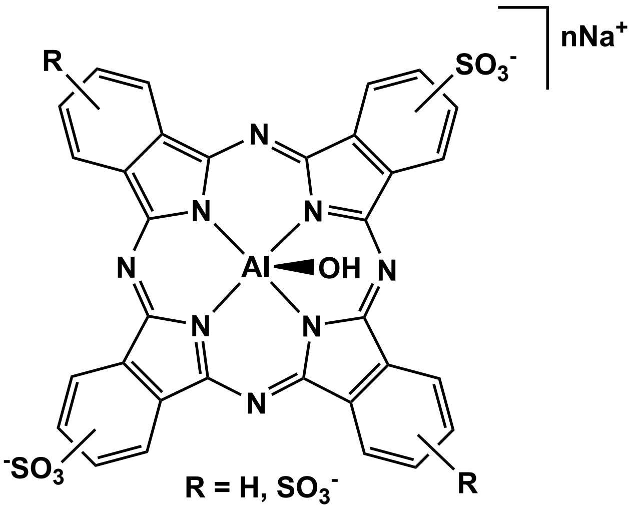	**Al1**<br>(Photosens)	**2**
Titanium		
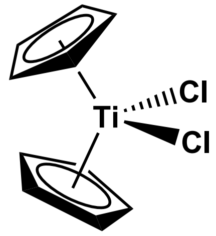	**Ti1**<br>($TiCp_2Cl_2$)	**2**
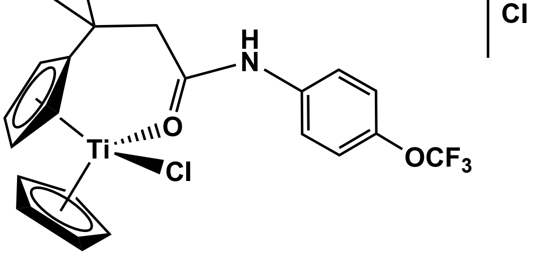	**Ti2**	**2**

Manganese		
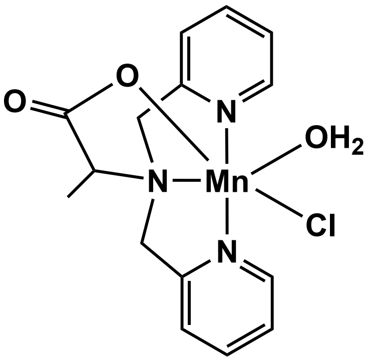	**Mn1** (Adpa-Mn)	**4**
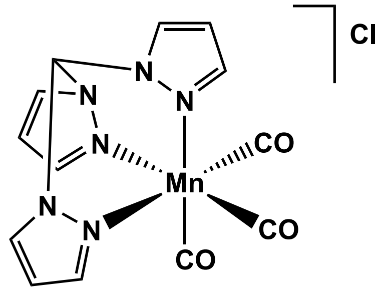	**Mn2**	**5**
Iron		
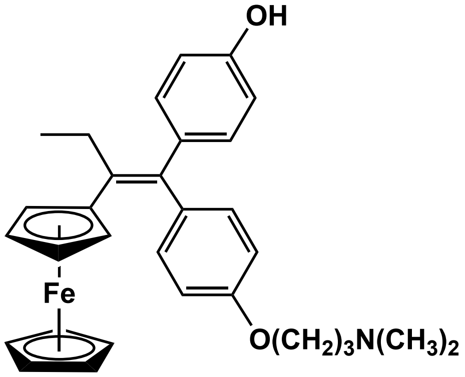	**Fe1** (Ferrocifen)	**2**
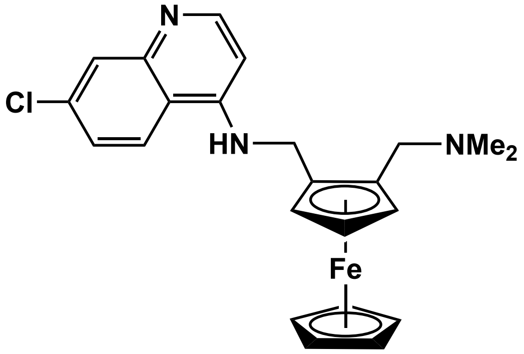	**Fe2** (Ferroquine)	**2, 3**

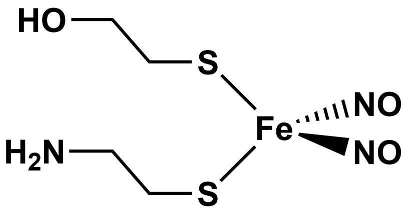	**Fe3**<br>(DNIC 2)	**4**
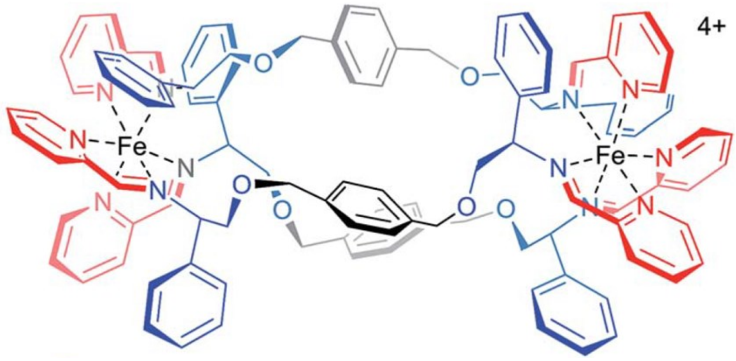	**Fe4**<br>[Fe2L3[1a]]Cl	**4**
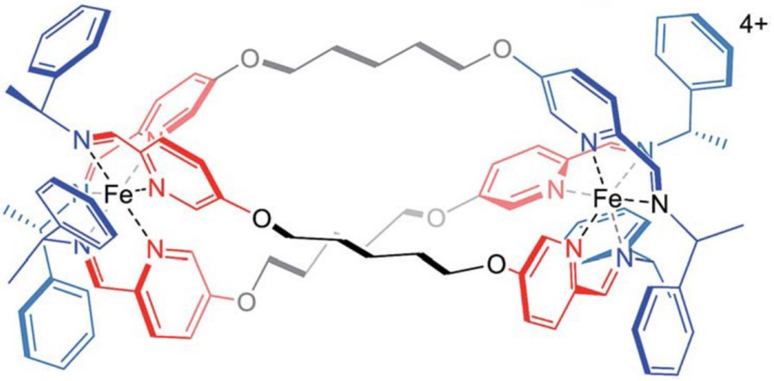	**Fe5**<br>[Fe2L3[2a–b]]Cl4	**4**

Cobalt		
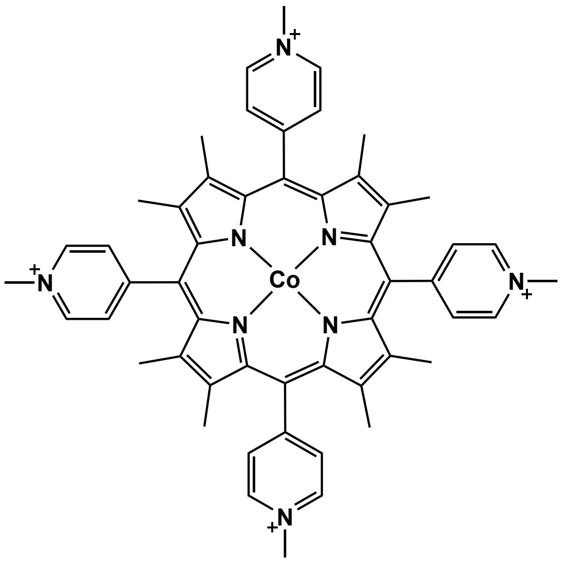	**Co1** (CoTMPyP)	**2**
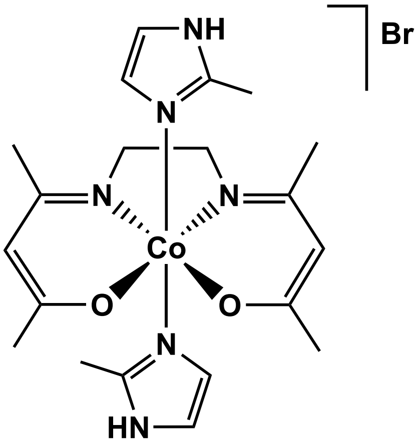	**Co2** (Doxovir)	**3**

Copper		
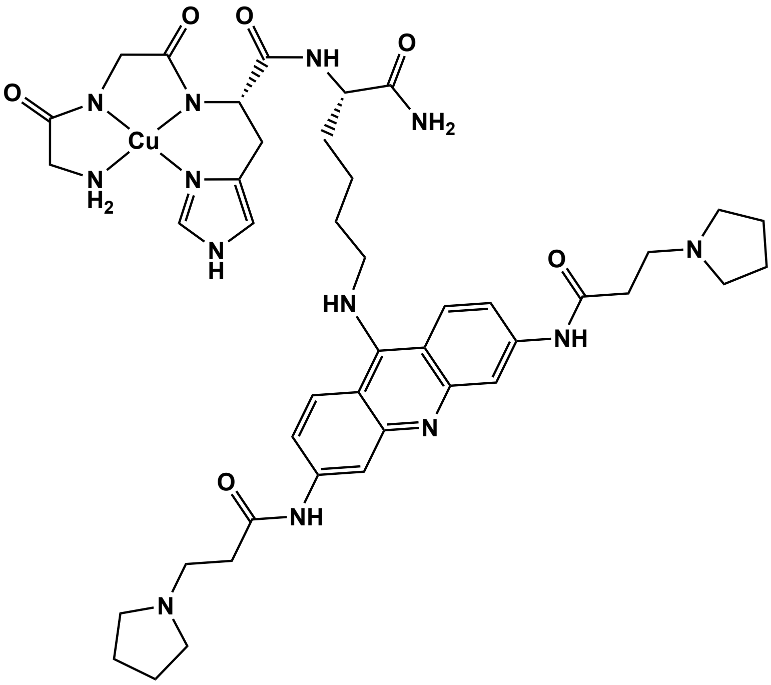	**Cu1**<br>(CuGGHK-Acr)	**2**
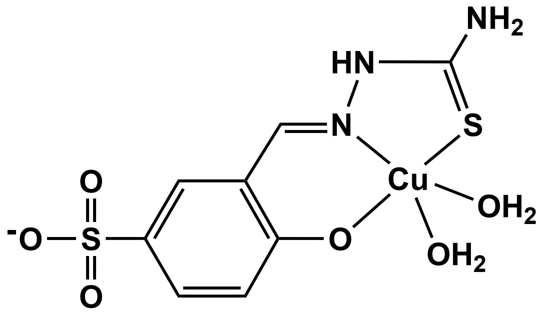	**Cu2**<br>(Cu-TSC)	**4**
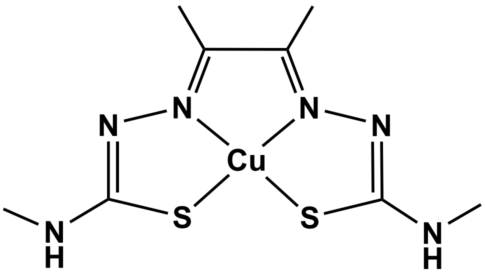	**Cu3**<br>[Cu(ATSM)]	**5**
Zinc		

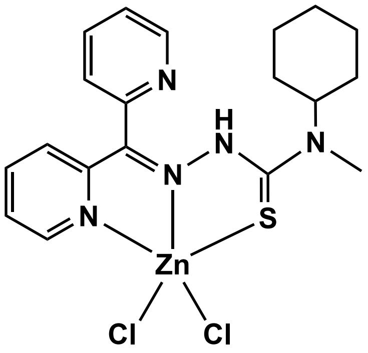	**Zn1**	**3**
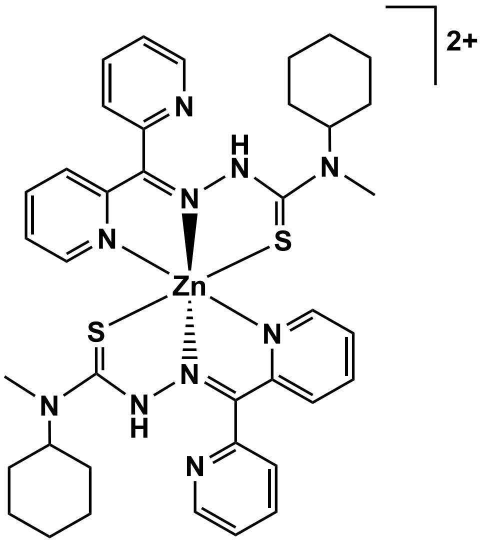	**Zn2**	**3**
Gallium		
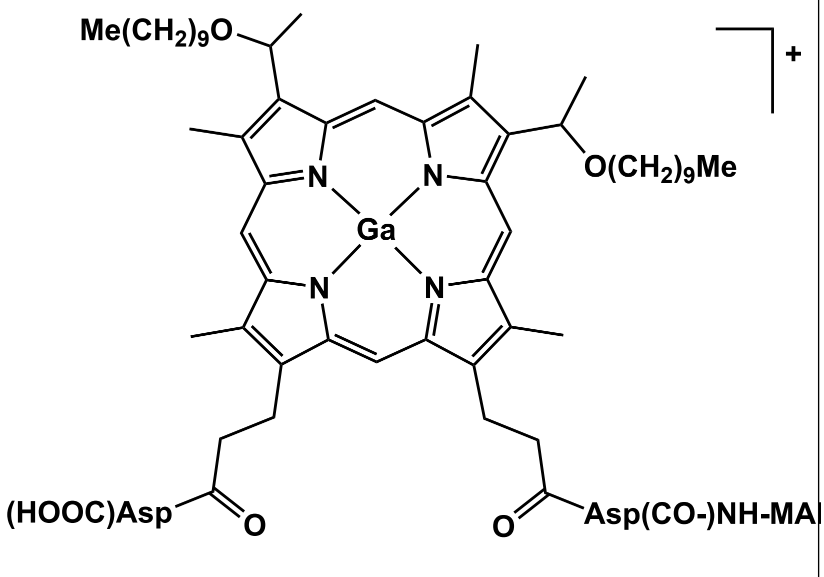	**Ga1**<br>(F39/ATX 70)	**2**
	**Ga2**<br>(KP46)	**3**

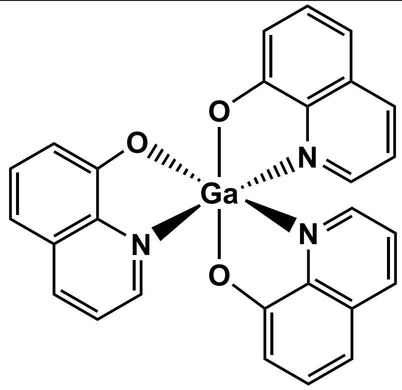		
Arsenic		
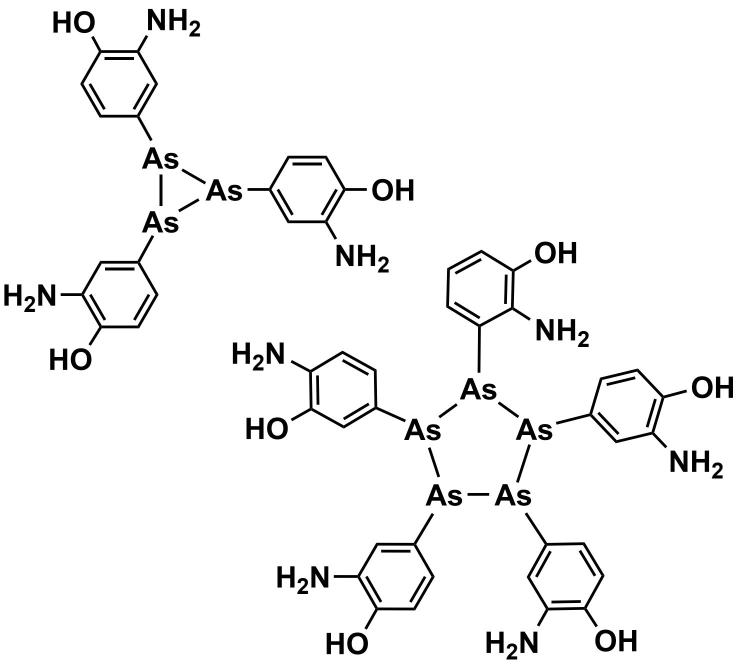	**As1**<br>(Salvarsan)	**1**
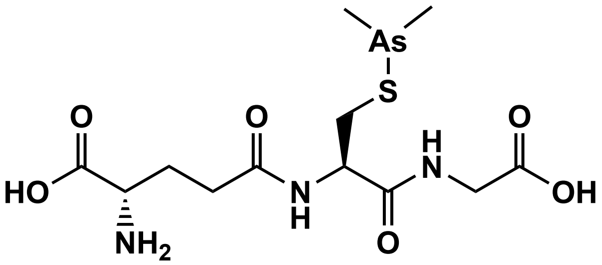	**As2**<br>(ZIO-101)	**3,4**
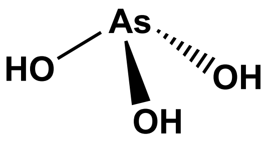	**As3**<br>(Arsenous acid)	**4**
Ruthenium		

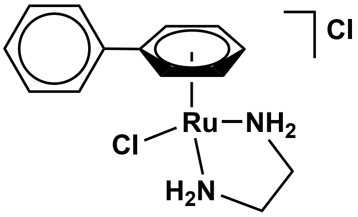	**Ru1**<br>(RM175)	**2**
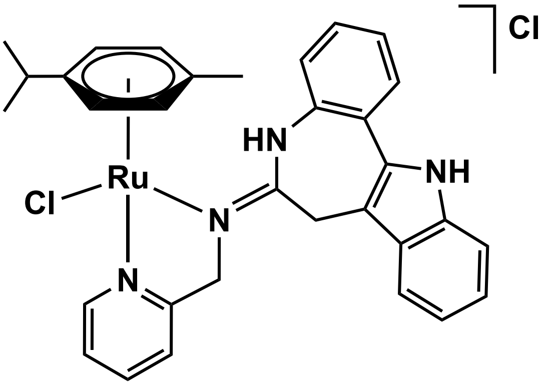	**Ru2**	**2**
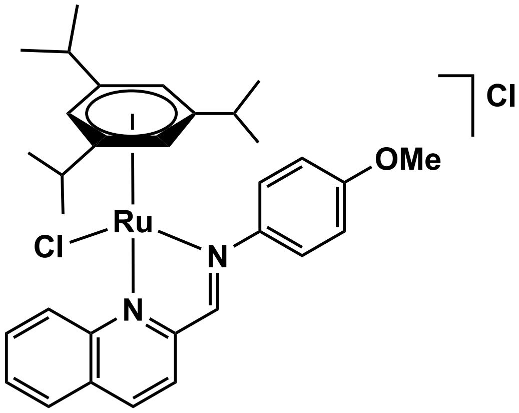	**Ru3**<br>(RAS-T1)	**2**
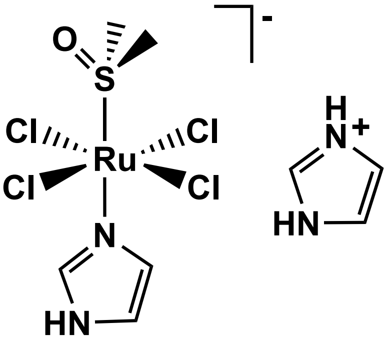	**Ru4**<br>(NAMI-A)	**2,3,4**

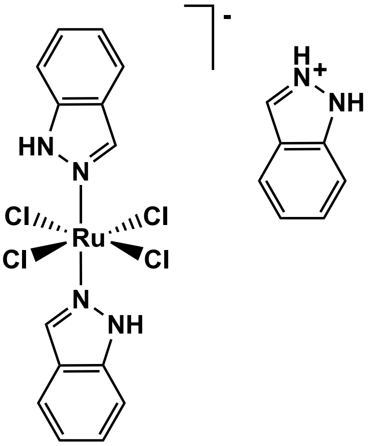	**Ru5** (KP1019)	**2,3,5**
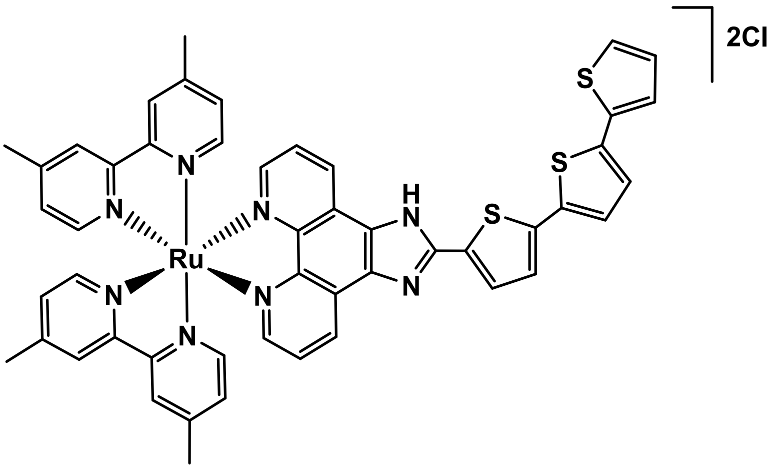	**Ru6** (TLD-1433)	**2,3,5**
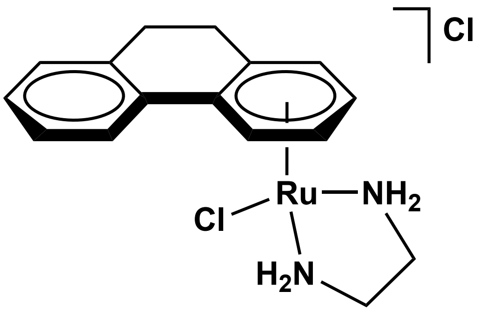	**Ru7** (AH63)	**2**
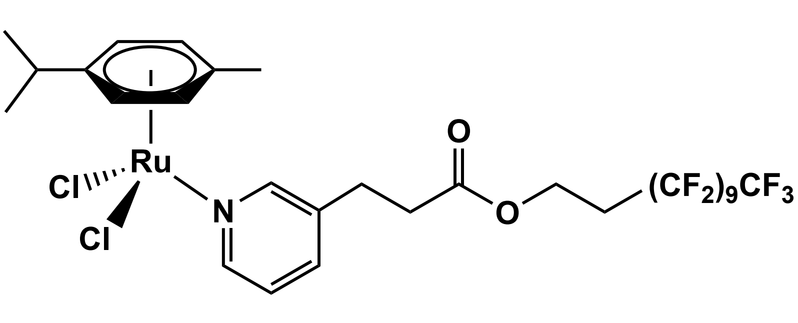	**Ru8**	**2**

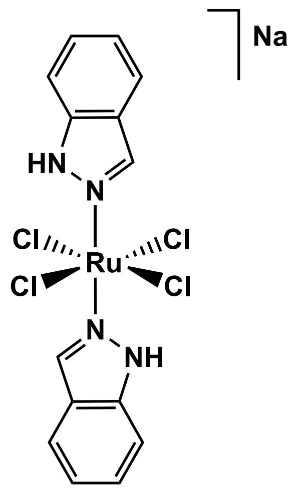	**Ru9** (KP1339)	**3,4**
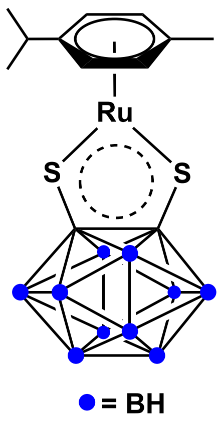	**Ru10**	**3**
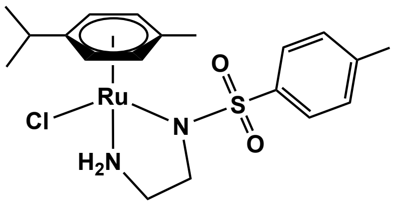	**Ru11**	**4**
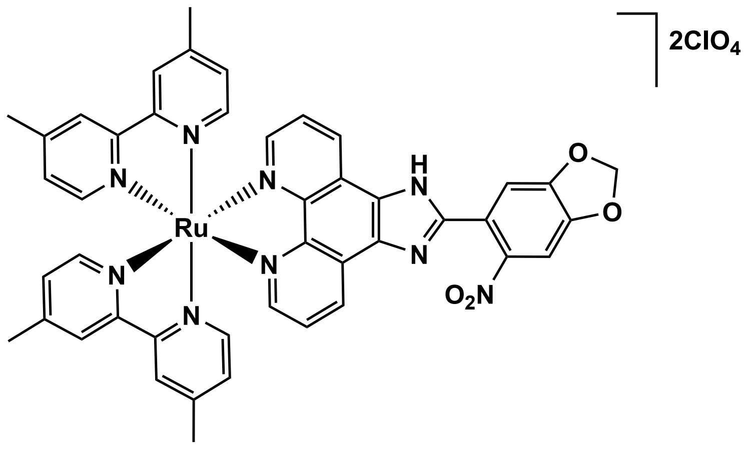	**Ru13**	**4**

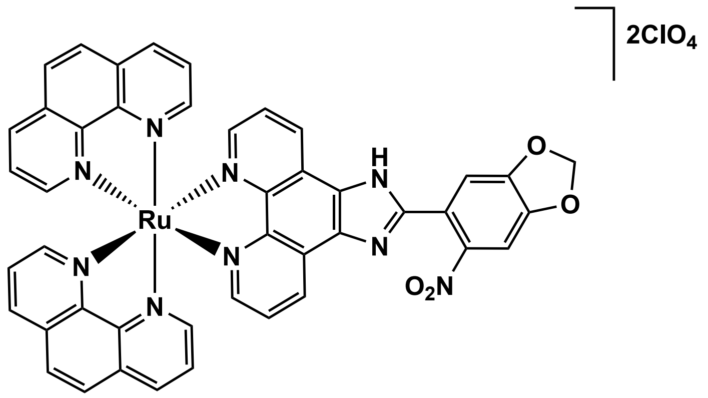	**Ru14**	**4**
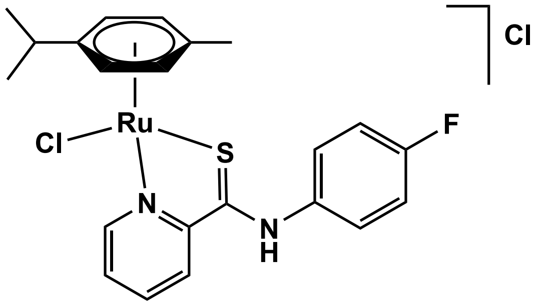	**Ru15** (Plecstatin-1)	**4,5**
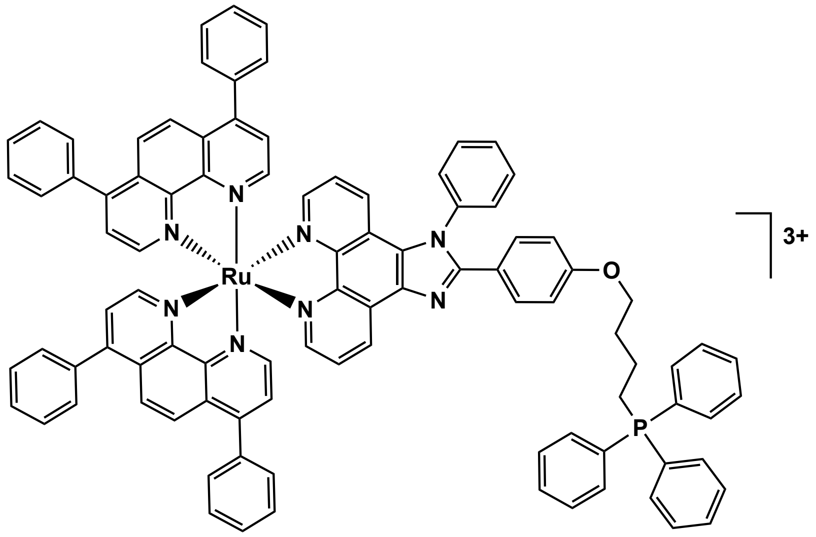	**Ru16**	**5**

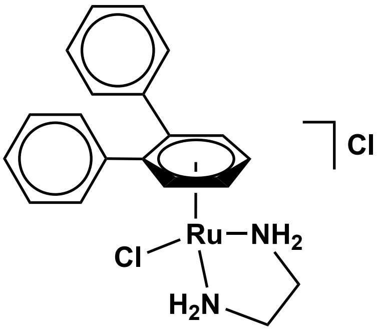	**Ru17**	**ESI**
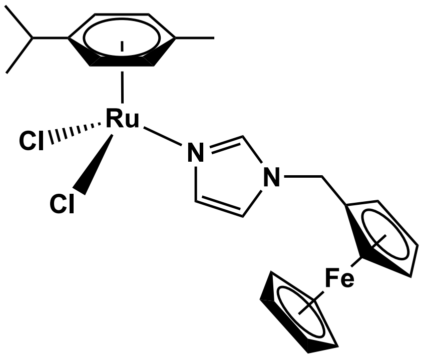	**Ru-Fe1**<br>(Ru-1-<br>FcMeIm)	**2**
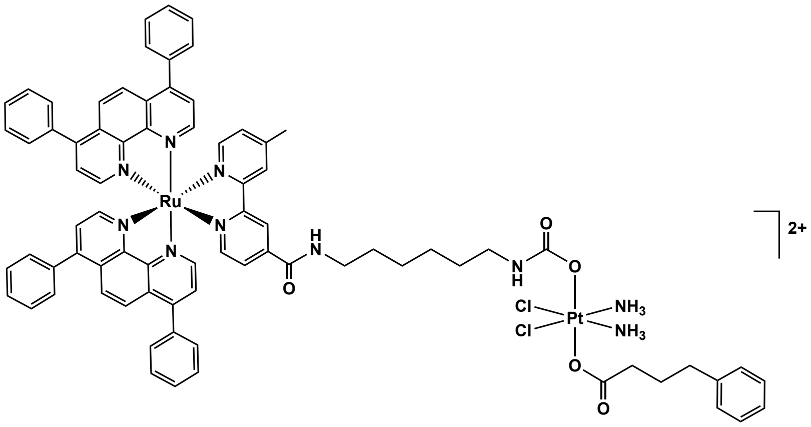	**Ru-Pt1**	**2**
Rhodium		
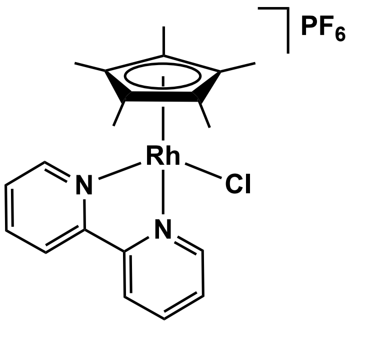	**Rh1**	**2**

Palladium		
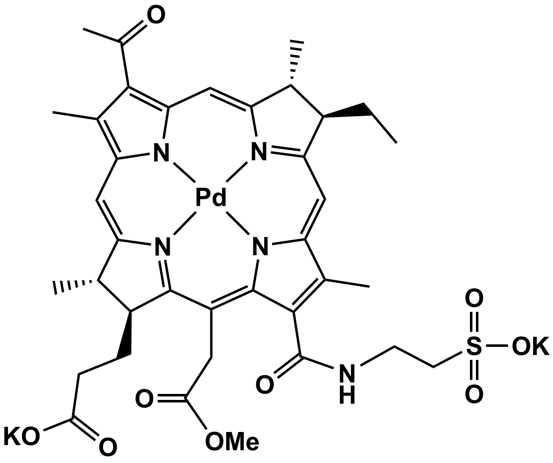	**Pd1** (TOOKAD-soluble)	**2**
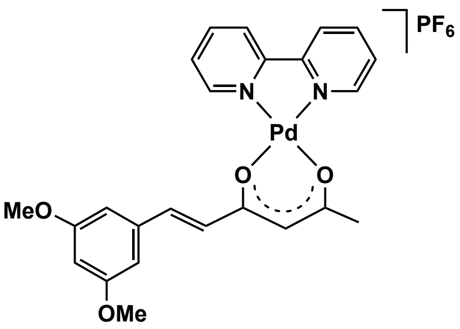	**Pd2**	**4**
Silver		
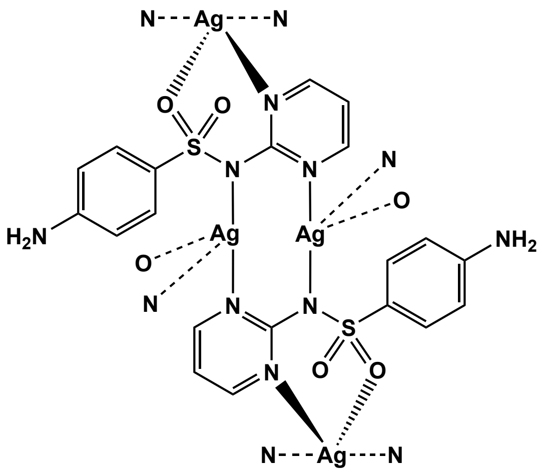	**Ag1** (Silver Sulfadiazine)	**3**

<table>
<tr><th colspan="3">Rhenium</th></tr>
<tr><td>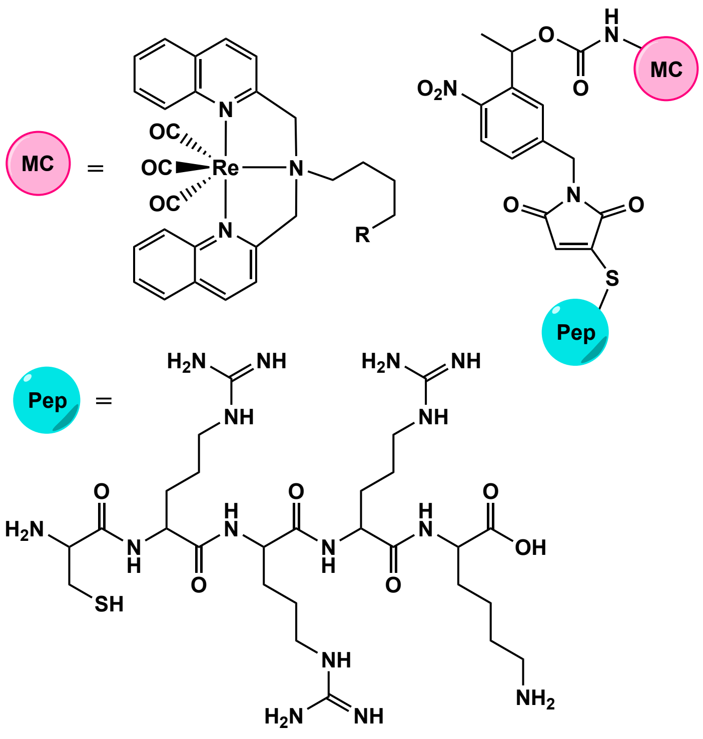
</td><td>Re1
(Re-PLPG-NLS)</td><td>2</td></tr>
<tr><td>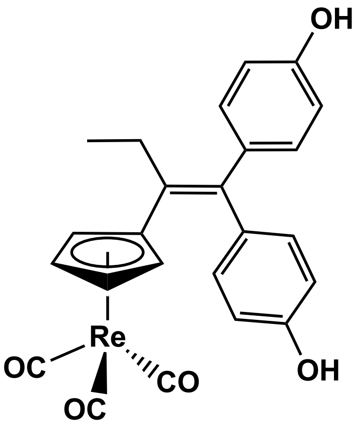
</td><td>Re2</td><td>5</td></tr>
</table>

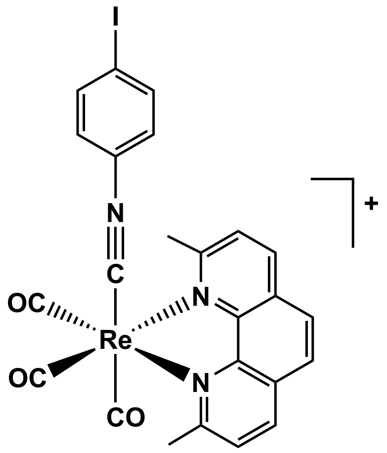	**Re3**	**5**
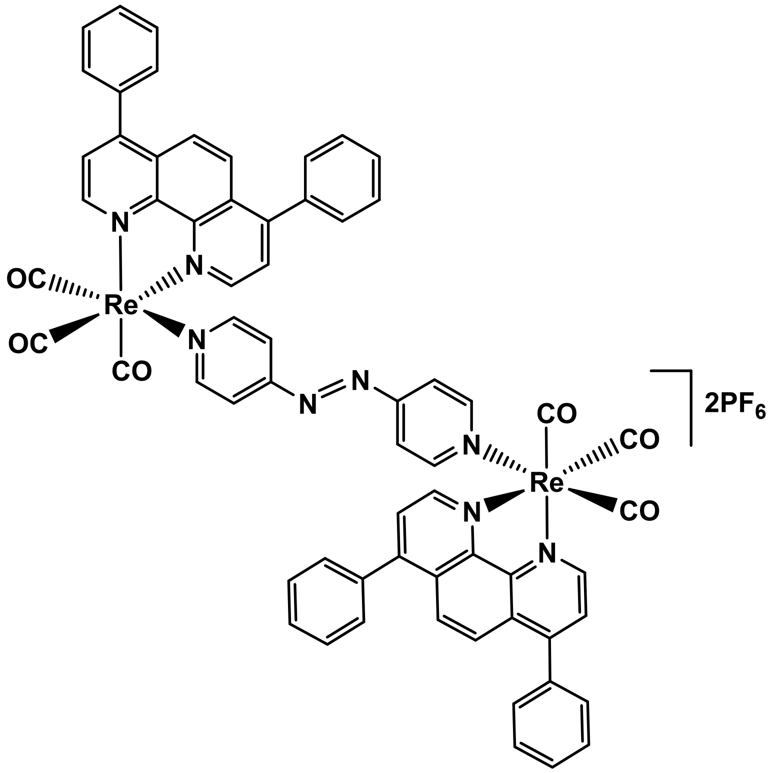	**Re-Re1**	**4**

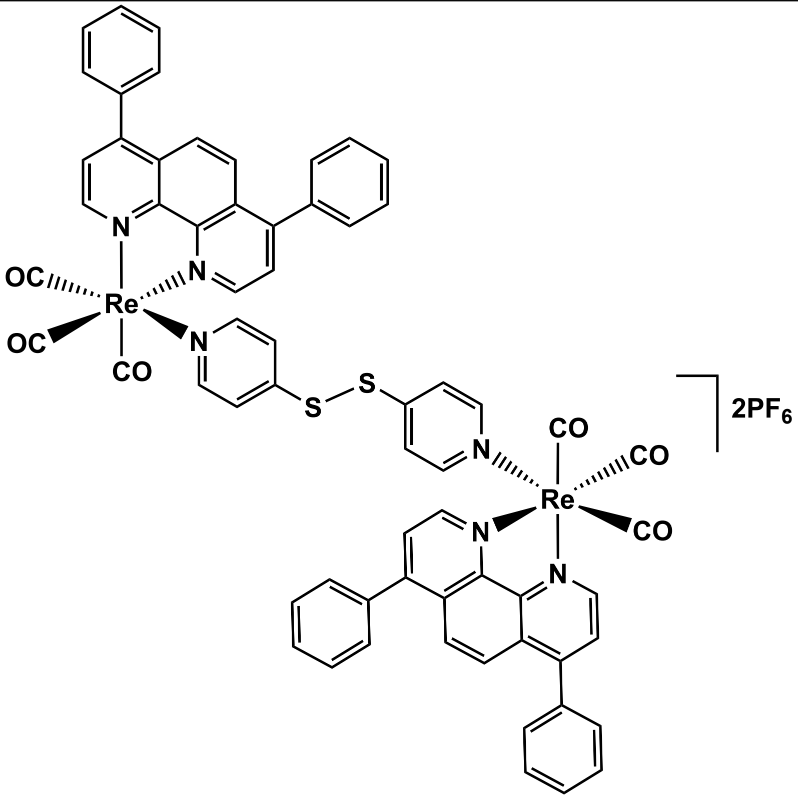	**Re-Re2**	**4**
Osmium		
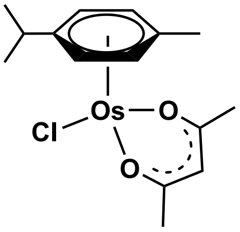	**Os1**	**2**
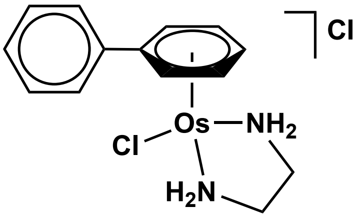	**Os2**	**2**
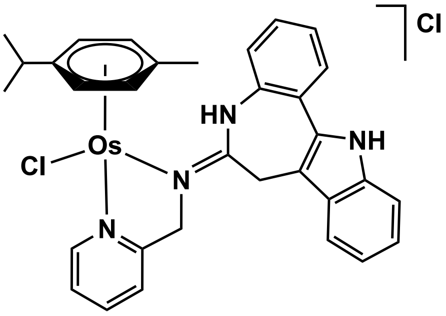	**Os3**	**2**

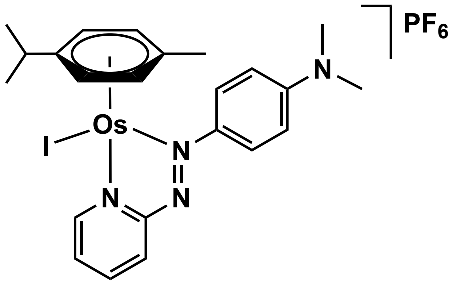	**Os4**<br>(FY26)	**2,4,5**
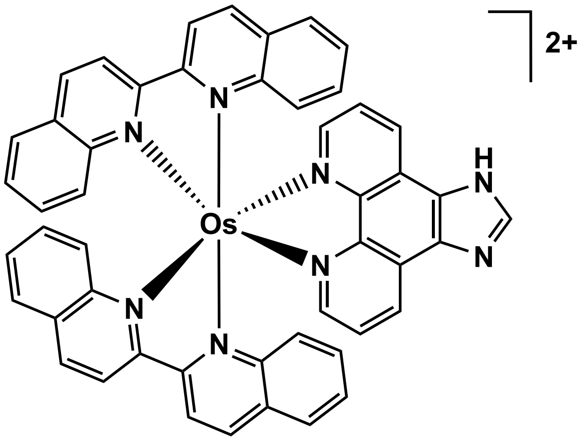	**Os5**<br>(TLD-1829)	**2**
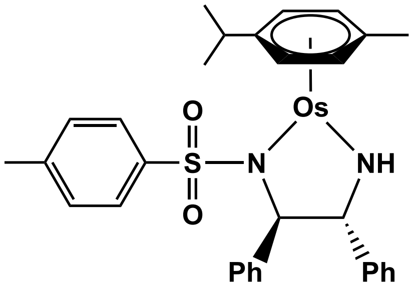	**Os6**	**2**
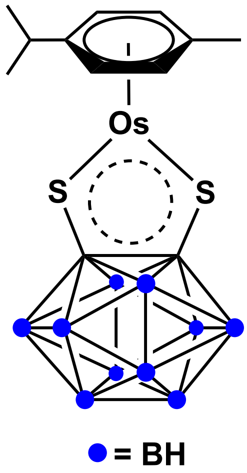	**Os7**	**3**

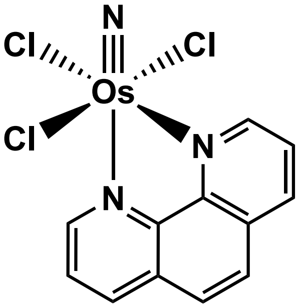	Os8	4
Iridium		
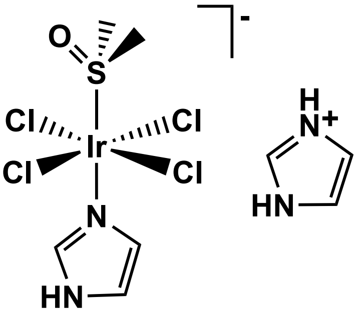	Ir1	2
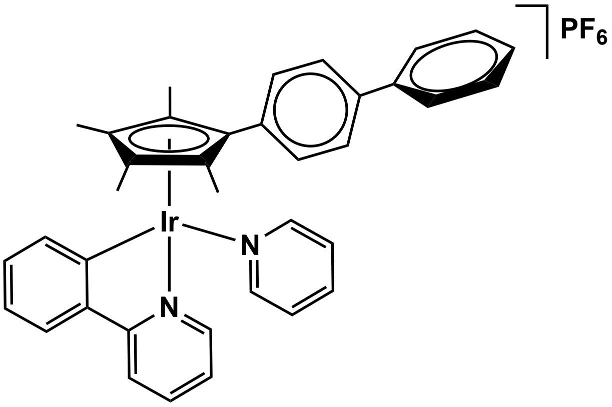	Ir2	2
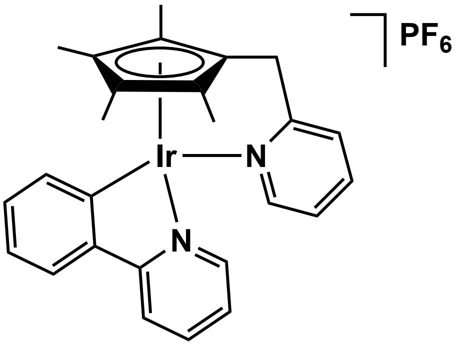	Ir3	2, 5
	Ir4	2

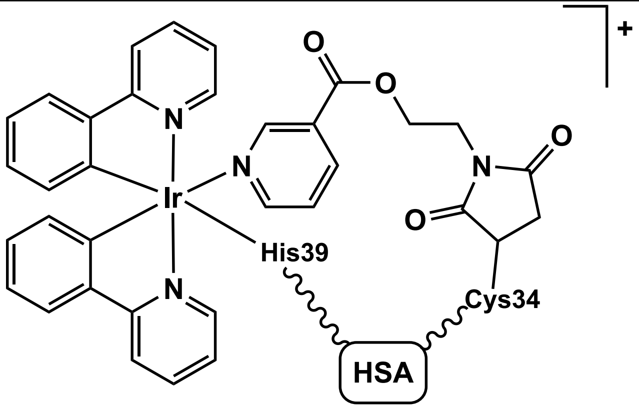	(Ir-HSA)	
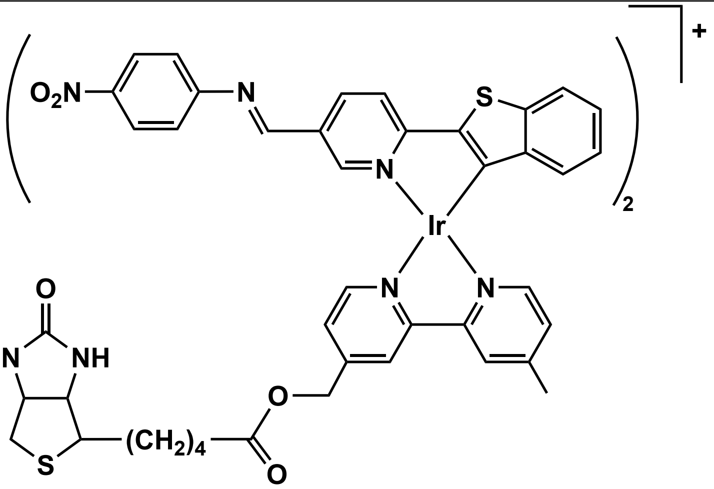	**Ir5**<br>(Ir-NB)	**2**
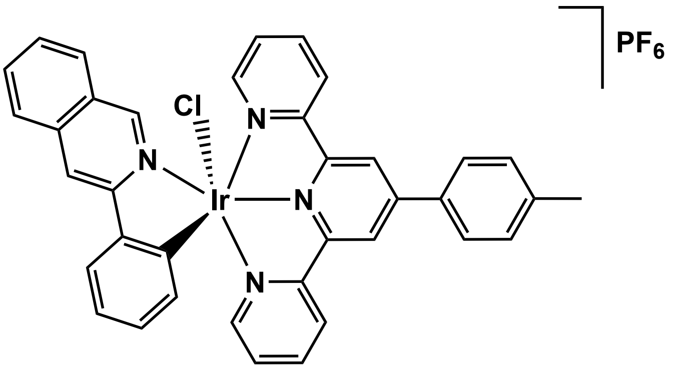	**Ir6**	**2**
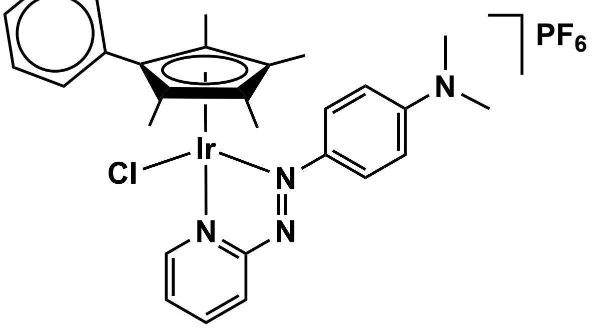	**Ir7**<br>(ZL109)	**3,4**
	**Ir8**	**3**

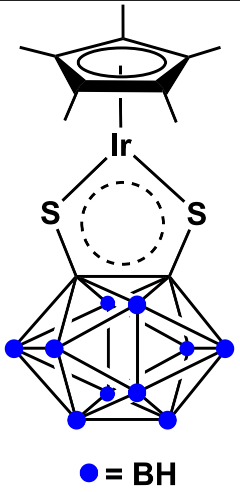		
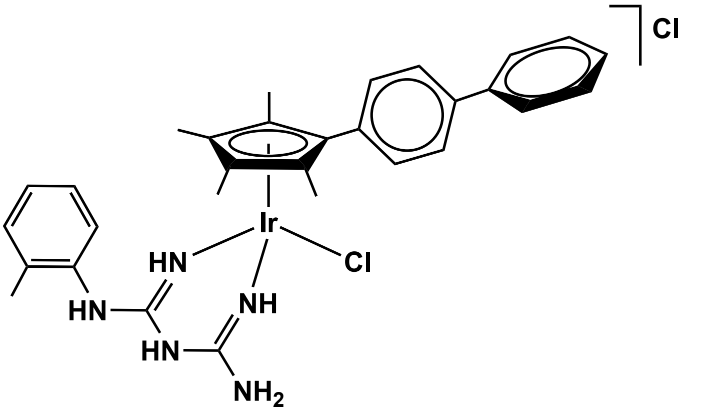	**Ir9**	**3**
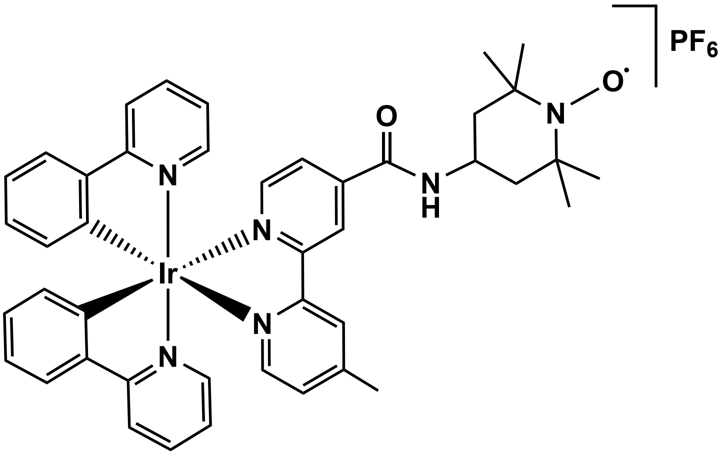	**Ir10**<br>(Ir-TEMPO1)	**4**
	**Ir11**	**5**

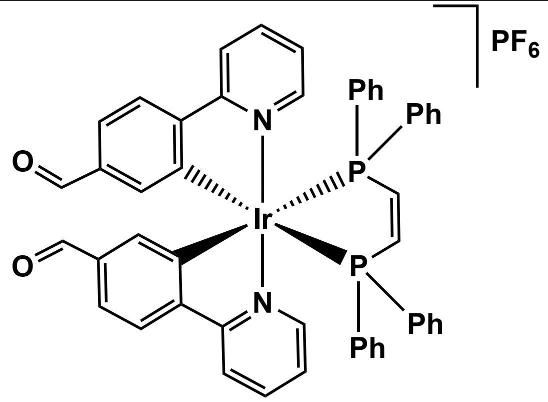		
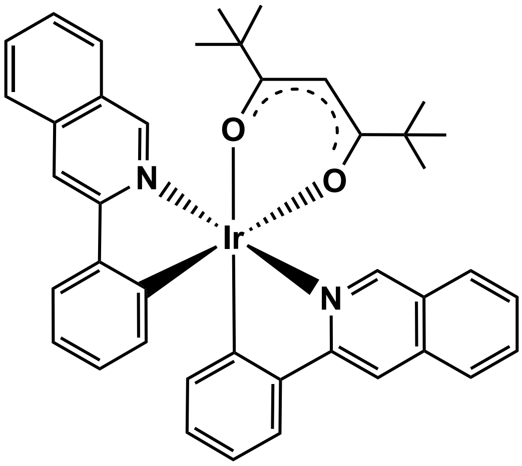	**Ir12**	**5**
Platinum		
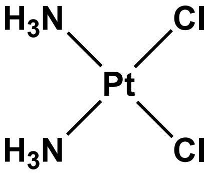	**Pt1**<br>(Cisplatin)	**1,2,3,4,5**
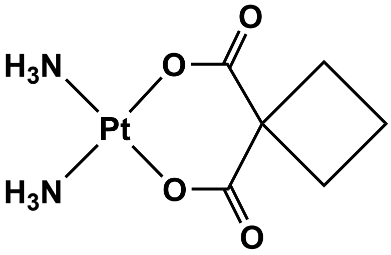	**Pt2**<br>(Carboplatin)	**1,2,3,4.5**
	**Pt3**	**1,3,4,5**

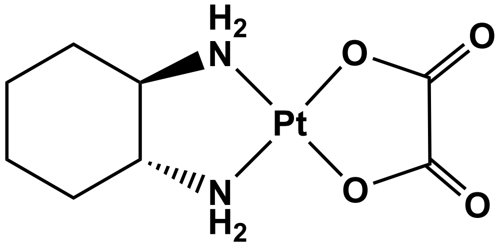	(Oxaliplatin)	
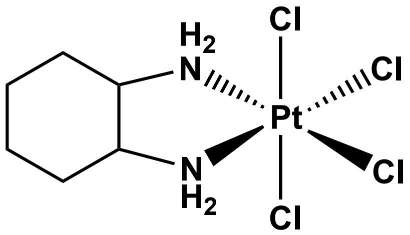	**Pt4**<br>(Tetraplatin)	**2**
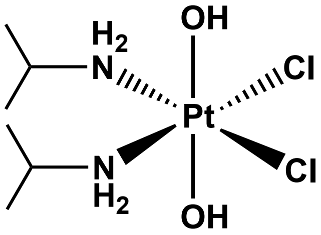	**Pt5**<br>(Iproplatin)	**2**
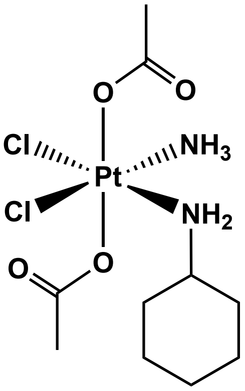	**Pt6**<br>(Satraplatin)	**2,3**
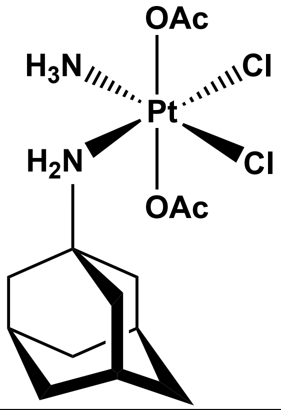	**Pt7**<br>(LA-12)	**2**
	**Pt8**	**2**

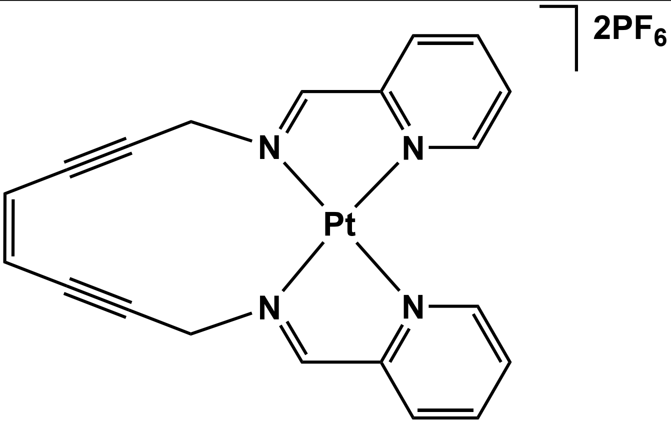	(Pt-pyEd)	
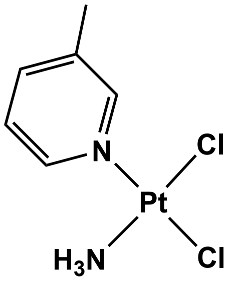	**Pt9** (Picoplatin)	**3**
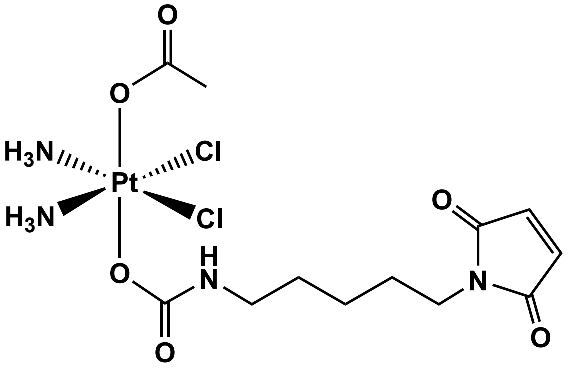	**Pt10** (BTP-114)	**3**
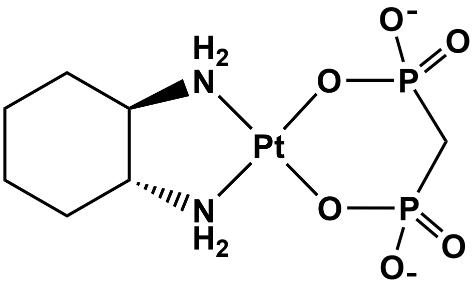	**Pt11** (PT-112)	**3**
	**Pt12** (ProLindac)	**3**

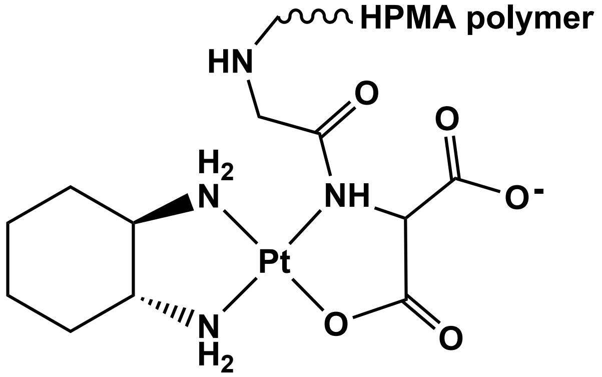		
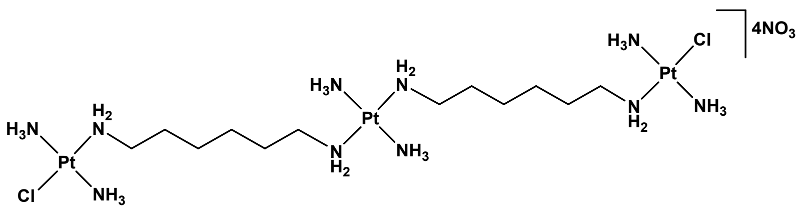	**Pt13** (Triplatin)	**3**
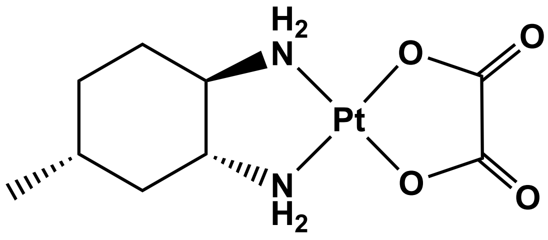	**Pt14** (KP1537)	**3**
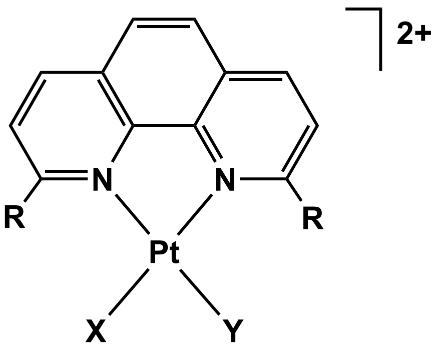 **R = H or Me** **X/Y = acy or pen**	**Pt15**	**3**

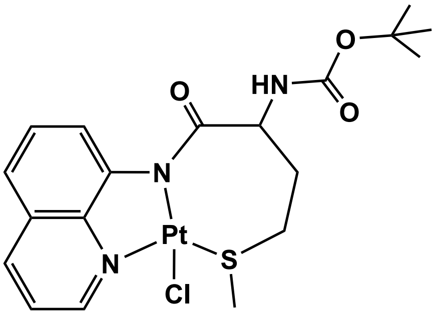	**Pt16**<br>(Mono-Pt)	**4**
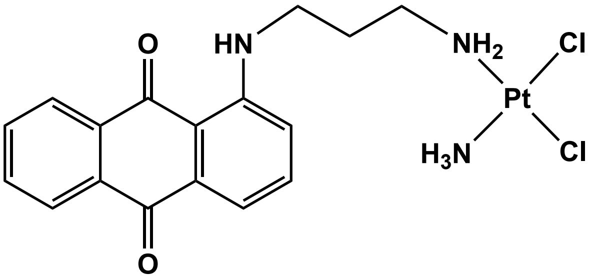	**Pt17**	**5**
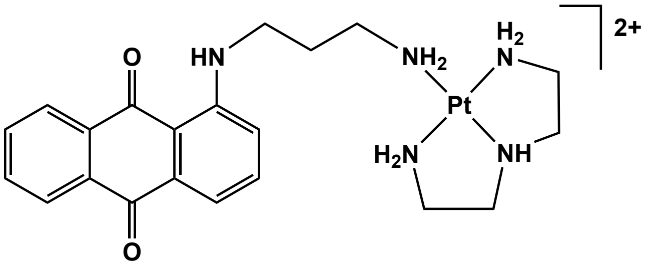	**Pt18**	**5**
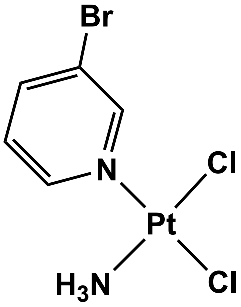	**Pt19**	**5**

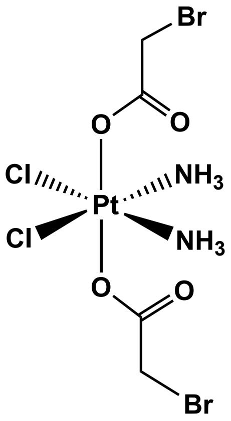	**Pt20**	**5**
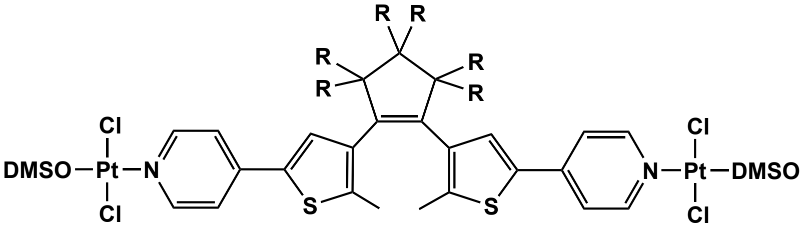 **R = H or F**	**Pt-Pt1**	**2**
Gold		
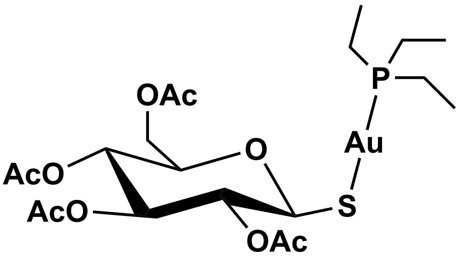	**Au1** (Auranofin)	**1,3**
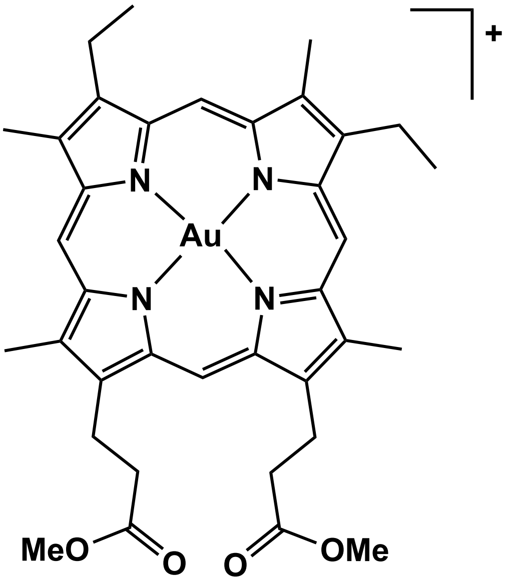	**Au2** (AuMesoIX)	**2,5**

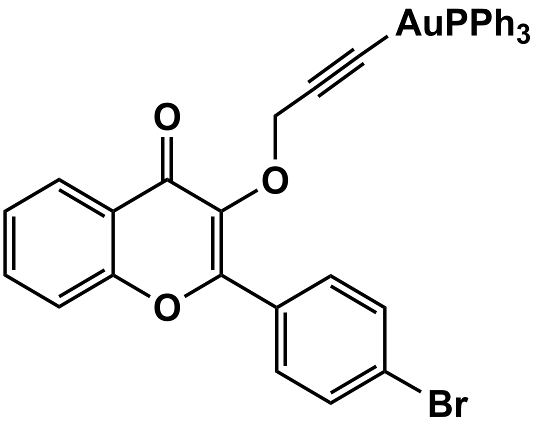	**Au3**	**4**
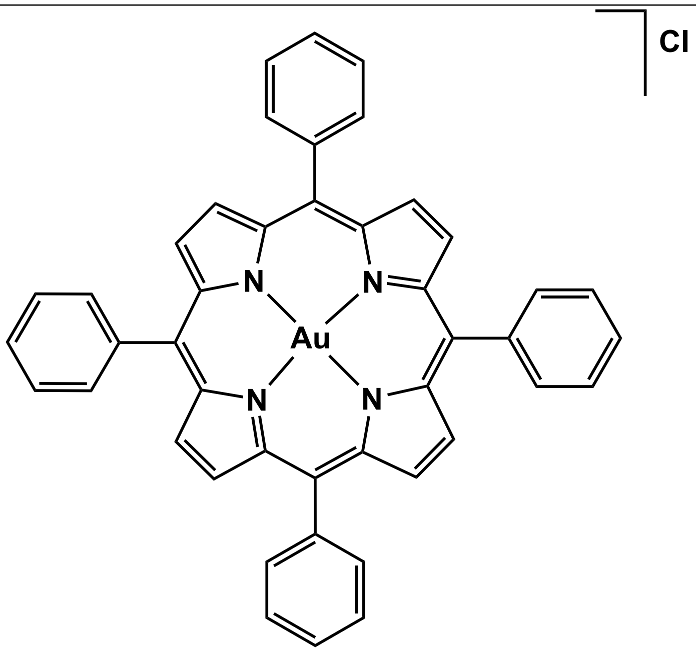	**Au4**	**4**
Bismuth		
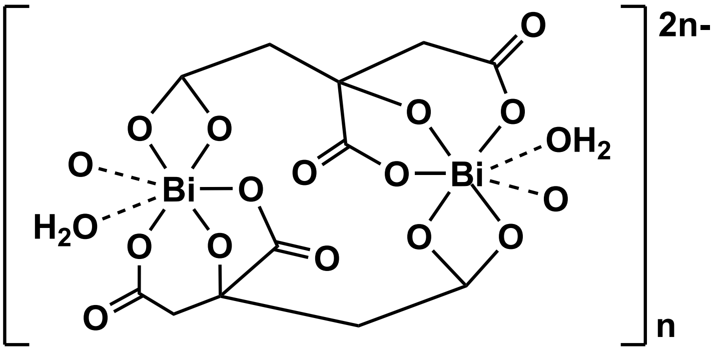	**Bi1**<br>(Bismuth citrate)	**3**
Lanthanum		

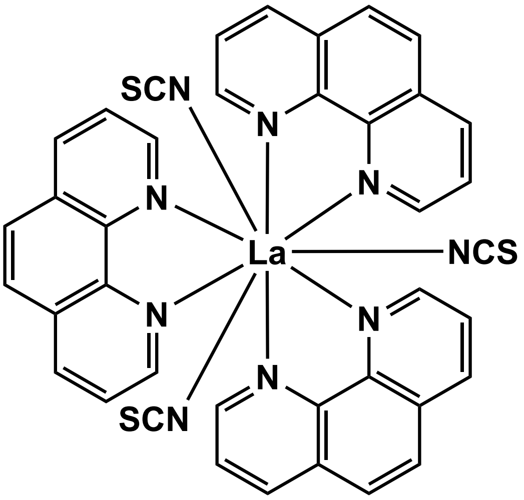	**La1** (KP772)	**3**
Europium		
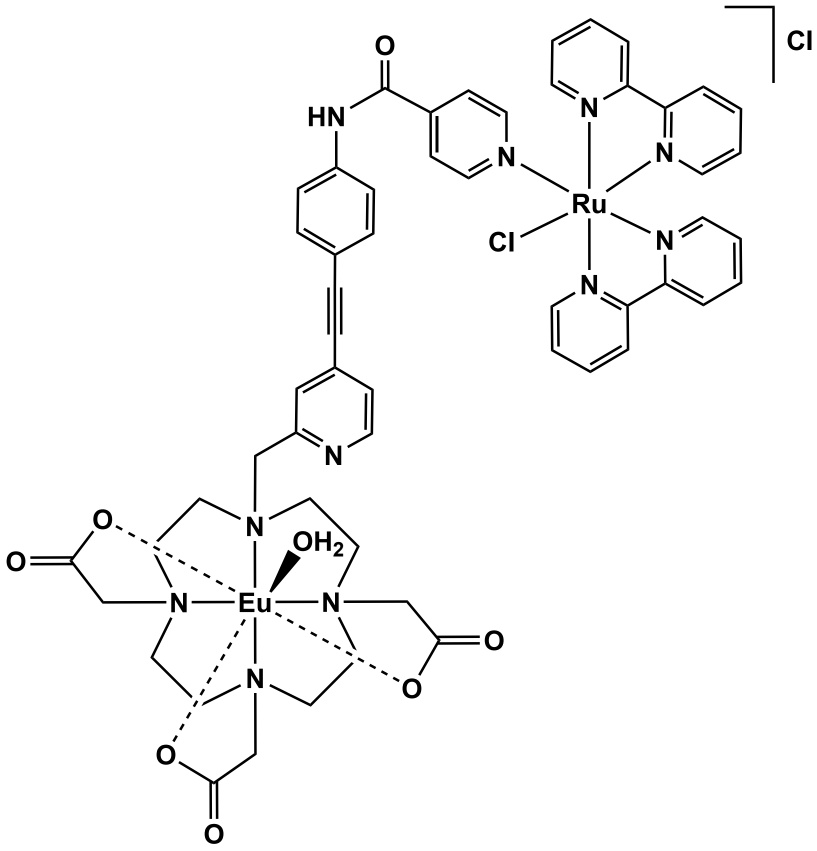	**Eu-Ru1**	**5**
Lutetium		

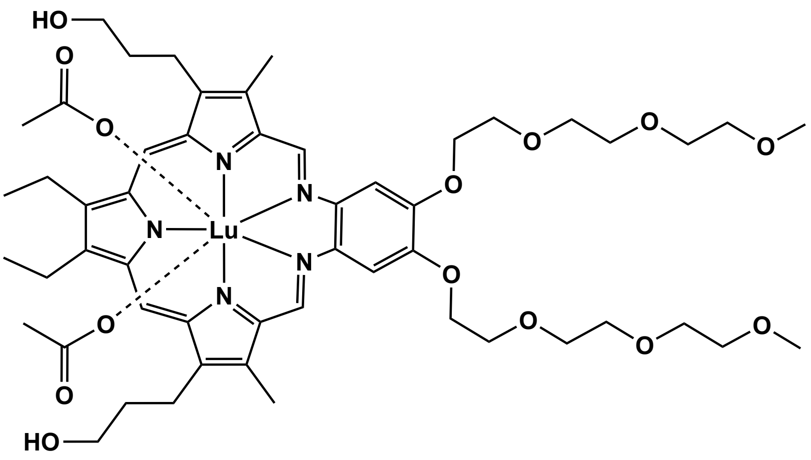	**Lu1** (Lutrin)	**2**
Complexes with various metal centres		
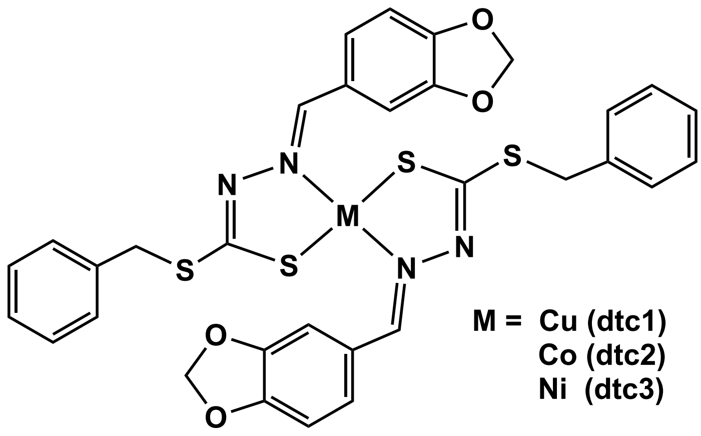	**M1**	**3**
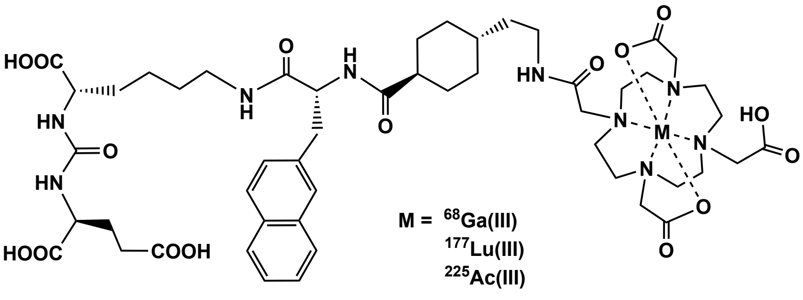	**M2** (PSMA-617 chelator)	**5**
Organic Drug		
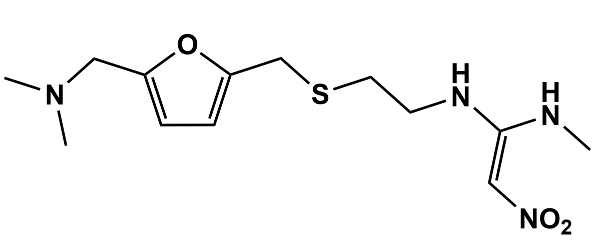	**L1** (Ranitidine)	**3**
